# A Concise Synthetic Strategy Towards the Novel Calcium-dependent Lipopeptide Antibiotic, Malacidin A and Analogues

**DOI:** 10.3389/fchem.2021.687875

**Published:** 2021-08-04

**Authors:** Nadiia Kovalenko, Georgina K. Howard, Jonathan A. Swain, Yann Hermant, Alan J. Cameron, Gregory M. Cook, Scott A. Ferguson, Louise A. Stubbing, Paul W. R. Harris, Margaret A. Brimble

**Affiliations:** ^1^ School of Chemical Sciences, The University of Auckland, Auckland, New Zealand; ^2^ School of Biological Sciences, The University of Auckland, Auckland, New Zealand; ^3^ Maurice Wilkins Centre for Molecular Biodiscovery, The University of Auckland, Auckland, New Zealand; ^4^ Department of Microbiology and Immunology, School of Biomedical Sciences, University of Otago, Dunedin, New Zealand

**Keywords:** calcium-dependent, lipopeptide, antibiotic, antimicrobial, solid-phase peptide synthesis

## Abstract

Malacidin A is a novel calcium-dependent lipopeptide antibiotic with excellent activity against Gram-positive pathogens. Herein, a concise and robust synthetic route toward malacidin A is reported, employing 9-fluorenylmethoxycarbonyl solid-phase peptide synthesis of a linear precursor, including late-stage incorporation of the lipid tail, followed by solution-phase cyclization. The versatility of this synthetic strategy was further demonstrated by synthesis of a diastereomeric variant of malacidin A and a small library of simplified analogues with variation of the lipid moiety.

## Introduction

The present COVID-19 pandemic shows how vulnerable society is to an infectious disease without access to an immediate effective treatment. Somewhat overshadowed by the current situation but equally as urgent, antimicrobial resistance (AMR) represents another ongoing global health crisis. According to World Health Organization (WHO) reports, as many as 2.8 million people contract infections caused by AMR pathogens in the U.S. alone, leading to more than 35,000 deaths annually (Centers for Disease Control and Prevention (U.S.), 2019; World Health Organization, 2019). Similar statistics are observed for Europe (Cassini et al., 2019). Although new stewardship programs and policies to increase AMR awareness and limit the use of existing antimicrobials are being introduced around the world, the existing clinical pipeline does not meet the demand to effectively combat increasing rates of AMR infections (World Health Organization, 2015). Thus, novel antimicrobial agents that can be developed into potential drug candidates are critically needed. Over the last 20 years antimicrobial peptides (AMPs) emerged as a rich yet underexplored source of such compounds. Development of alternative platforms for AMP discovery and methods for synthetic optimizations of natural scaffolds are yielding many promising examples of clinically relevant AMPs (Jin, 2020; Liu et al., 2021).

In 2018, Brady et al. reported the isolation of malacidin A (**1**) as part of an extensive metagenomic mining study of bacterial DNA obtained from soil samples in search of novel bioactive natural products (Owen et al., 2013; Hover et al., 2018). This novel AMP possesses potent bioactivity against a range of Gram-positive strains including multi-drug resistant pathogens such as methicillin-resistant *Staphylococcus aureus* (MRSA) (minimum inhibitory concentrations (MIC) 0.2–0.8 μg ml^−1^) and vancomycin-resistant *Enterococcus faecium* (VRE) (MIC 0.8–2.0 μg ml^−1^) (Hover et al., 2018). Malacidin A (**1)** belongs to a family of calcium-dependent lipopeptide antibiotics (CDLAs) that exhibit their activity upon binding to calcium ions. The CDLA family is represented by several sub-groups of potent antibiotics: A21978C complex, which includes the antibiotic of last resort, daptomycin; A54145 complex; calcium dependent antibiotics (CDAs); friulimicins, of which friulimicin B reached Phase I clinical trials; amphomycins, of which, MX-2401 (a semi-synthetic analogue), progressed to late-stage preclinical development; glycinocins and, recently, cadasides (Wood and Martin, 2019; Wu et al., 2019). Malacidin A (**1**) is structurally unique compared to other common CDLA members in that the canonical Asp-AA-Asp-Gly (AA = Gly or d-amino acid) calcium-binding motif lacks the spacer residue, AA, and the first Asp residue is replaced by an unusual 3-hydroxy aspartic acid (3-HyAsp) (Hover et al., 2018). Preliminary mechanistic studies of malacidin A revealed binding to lipid II, a different target compared to other CDLAs. Malacidin A (**1**) was also found to be non-cytotoxic and did not induce resistance after repeated exposure to *S. aureus* (Hover et al., 2018). These features render **1** an exciting target for development as a novel antibiotic. The key step toward this goal is design of robust synthetic routes that would enable facile access to the lead compound and analogues thereof to establish structure activity relationships (SARs).

Herein, we report a concise synthetic strategy toward malacidin A (**1**) as demonstrated by the synthesis of a diastereomeric variant and simplified analogues thereof. The key steps involve preparation of the key linear precursor by 9-fluorenylmethoxycarbonyl (Fmoc)-solid-phase peptide synthesis (SPPS), followed by tail-to-side chain solution-phase cyclisation.

**FIGURE 1:**
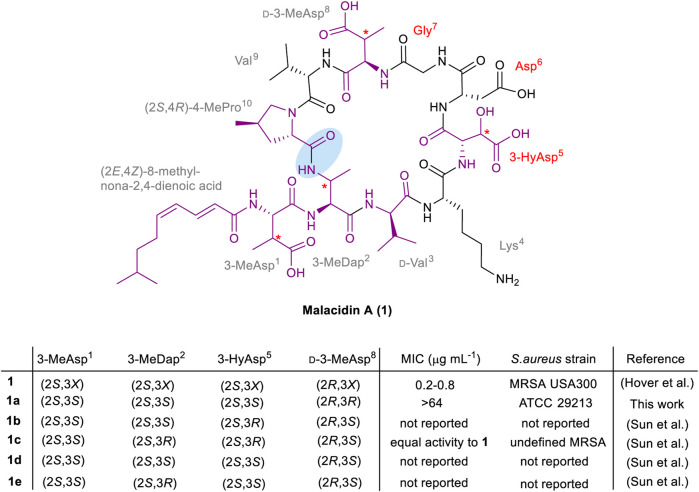
Structure of malacidin A (**1**); non-canonical amino acids and the lipid tail are highlighted in purple; the undefined stereochemistry of β-carbon centers is indicated with an asterisk; the calcium-binding region residues are specified in red; the macrolactam bond is highlighted with blue shading. The stereochemistry and MIC values of the isolated malacidin A (**1**) (Hover et al., 2018) and synthesized diastereomers [this work **1a**, the Li group **1b**–**1e** (Sun et al., 2020)] are shown above; *X* in **1** represents undefined stereochemistry.

Structurally, malacidin A (**1**) consists of a 9-mer cyclic core and a single exocyclic amino acid, 3-methylaspartic acid (3-MeAsp^1^). The lipopeptide is acylated at the *N*-terminus with an unusual polyunsaturated lipid tail, (2*E*,4*Z*)-8-methylnona-2,4-dienoic acid (Figure 1). The macrolactam bond is formed between the side chain of 3-methyldiaminopropinoic acid (3-MeDap^2^) and the *C*-terminal carboxyl group of (4*R*)-4-methylproline ((4*R*)-4-MePro^10^). The sequence of malacidin A (**1**) is unusually rich in non-canonical amino acids that include the aforementioned 3-MeAsp^1^, 3-MeDap^2^, 3-HyAsp^5^, (4*R*)-4-MePro^10^, as well as d-Val^3^ and d-3-MeAsp^8^. Upon discovery of malacidin A (**1**), the exact configuration of the β-carbon centers in 3-MeAsp^1^, 3-MeDap^2^, 3-HyAsp^5^ and d-3MeAsp^8^ could not be determined, thus giving rise to 16 possible diastereomers (Hover et al., 2018). Therefore, it was decided to concentrate synthetic efforts on a diastereomer of malacidin A (**1a**) that contained (2*S*,3*S*)-3-MeAsp^1^, (2*S*,3*S*)-3-MeDap^2^, (2*S*,3*S*)-3-HyAsp^5^ and (2*R*,3*R*)-d-3-MeAsp^8^. This choice was based on structural and biosynthetic gene cluster similarities of malacidin A (**1**) and friulimicin B (Müller et al., 2007; Hover et al., 2018).

During the course of this work, the first total synthesis of malacidin A (**1c**) and three diastereomers (**1b**, **1d** and **1e**) was reported by Sun *et al.*, establishing the exact stereoconfiguration of the natural product (Sun et al., 2020). This involved Fmoc-SPPS of a branched precursor followed by solution-phase β-hydroxy-mediated cyclization between 3-HyAsp^5^ and a salicylaldehyde ester of Lys^4^, employing the Ser/Thr ligation approach developed by the Li group (Li et al., 2010). Peptides **1b**-**1e** were prepared in 2–6% overall yields with variation at residue positions 2 and 5, employing appropriately protected derivatives of (2*S*,3*S*)- or (2*S*,3*R*)-3-MeDap, and (2*S*,3*S*)- or (2*S*,3*R*)-3-HyAsp, respectively. A close match between the NMR spectra of synthetic **1c**, bearing (2*S*,3*S*)-3-MeAsp^1^, (2*S*,3*R*)-3-MeDap^2^, (2*S*,3*R*)-3-HyAsp^5^ and (2*R*,3*S*)-3-MeAsp^8^ residues, and isolated malacidin A (**1**), established the exact stereochemistry of the natural product (Sun et al., 2020). Along with the observed bioactivity, advanced Marfey’s analysis of the synthetic amino acids used to prepare **1c**, compared to those obtained by hydrolysis of isolated malacidin A (**1**) further confirmed the stereochemical assignment. The MIC values were not reported for the other three diastereomers of malacidin A (**1b**, **1d** and **1e**), presumably due to their inactivity; however, this information would be required to establish an SAR. As the synthesis of the diastereomer **1a** described herein commenced before the total synthesis and determination of the actual configuration of malacidin A (**1**) was published, our original synthetic strategy was still pursued to assess the significance of the opposite β-stereocenters of the three non-canonical residues (3-MeDap^2^, 3-HyAsp^5^ and d-3-MeAsp^8^) on the activity of the antibiotic.

## Results and Discussion

### Synthesis of Unnatural Amino Acid Building Blocks

Before synthesis of the diastereomer of malacidin A (**1a**) could commence, appropriately protected building blocks of (2*S*,3*S*)-3-MeAsp^1^, (2*S*,3*S*)-3-MeDap^2^, (2*S*,3*S*)-3-HyAsp^5^, (2*R*,3*R*)-3-MeAsp^8^ and (2*S*,4*R*)-4-MePro^10^ and (2*E*,4*Z*)-8-methylnona-2,4-dienoic acid were required to facilitate incorporation using solid-phase synthesis.

#### Synthesis of (2*S*,3*S*)-Fmoc-3-MeAsp(O*t*Bu)-OH (2) and (2*R*,3*R*)-Fmoc-3-MeAsp(O*t*Bu)-OH (7)

**SCHEME 1:**
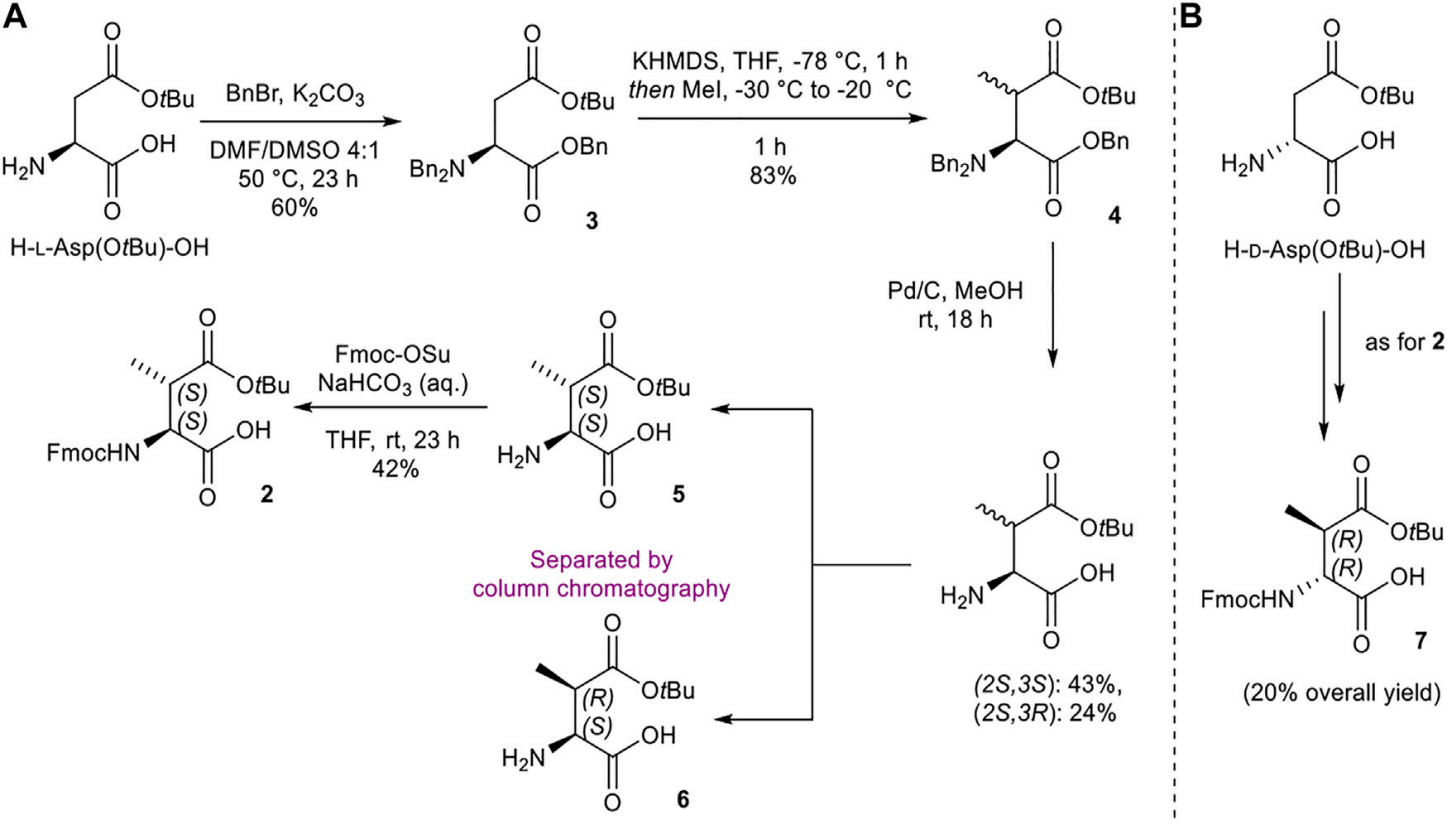
Synthesis of (2*S*,3*S*)-Fmoc-3-MeAsp(O*t*Bu)-OH (**2**) and (2*R*,3*R*)-Fmoc-3-MeAsp(O*t*Bu)-OH (**7**) building blocks.

The initial synthetic strategy of (2*S*,3*S*)-Fmoc-3-MeAsp(O*t*Bu)-OH (**2**) from *H*-l-Asp(O*t*Bu)-OH took inspiration from similar work reported by Giltrap et al. (2017), with the preparation of methylated l-Asp **4** adapted from procedures outlined by Xue et al. (2002) (Scheme 1A). Tri-benzylation, methylation, and global benzyl deprotection of *H*-l-Asp(O*t*Bu)-OH afforded the (2*S*,3*S*) and (2*S*,3*R*) diastereomers (**5** and **6**, respectively) as a 1:1 mixture, which were separated by silica gel flash chromatography. Fmoc protection of **5** delivered the desired (2*S*,3*S*) building block **2**. The enantiomeric (2*R*,3*R*) analogue **7** was prepared in an analogous fashion from *H*-d-Asp(O*t*Bu)-OH (Scheme 1B).

#### Synthesis of (2*S*,3*S*)-Fmoc-3-MeDap(Dde)-OH (8)

**SCHEME 2:**
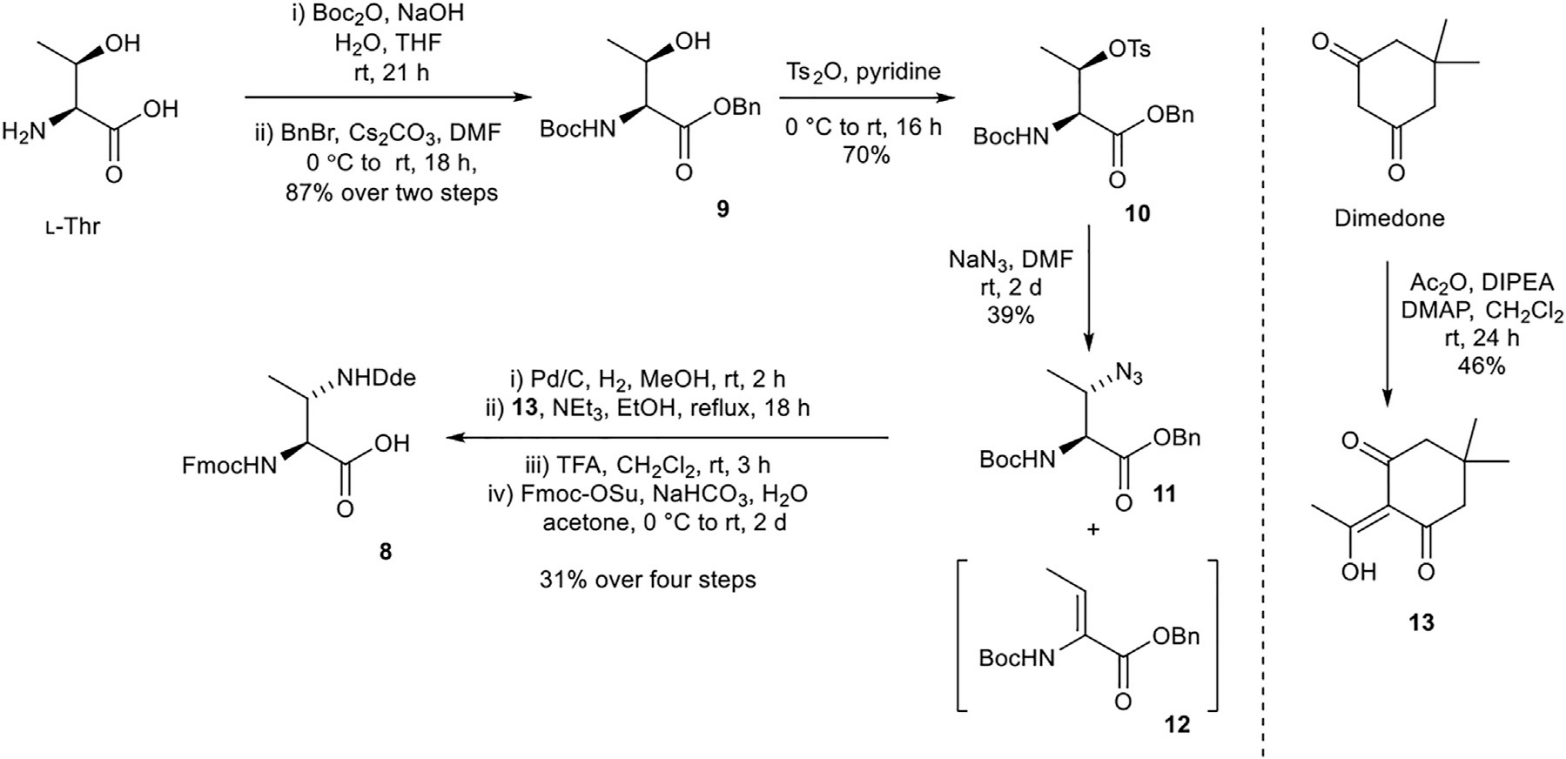
Synthesis of (2*S*,3*S*)-Fmoc-3-MeDap(Dde)-OH (**8**).

(2*S*,3*S*)-Fmoc-3-MeDap(Dde)-OH (**8**) was prepared from procedures adapted from Martín *et al.*, utilizing a *N*-(1-(4,4-dimethyl-2,6-dioxocyclohexylidene)ethyl) (Dde) protecting group (Scheme 2) (Martín et al., 2014). *tert*-Butyloxycarbonyl (Boc) and benzyl protections of l-threonine afforded **9**. Sequential tosylation and nucleophilic substitution with sodium azide yielded azide **11** with the correct (3*S*) stereochemistry, along with a considerable amount of undesired elimination byproduct **12**. Hydrogenation and Dde protection of the resultant amine with **13** (prepared from dimedone) (Armaly et al., 2017), followed by exchange of the *N* ^α^-Boc protecting group for Fmoc protecting group afforded the desired building block **8**.

#### Synthesis of (2*S*,3*S*)- Fmoc-3-Hy(TBS)Asp(O*t*Bu)-OH (14)

**SCHEME 3:**
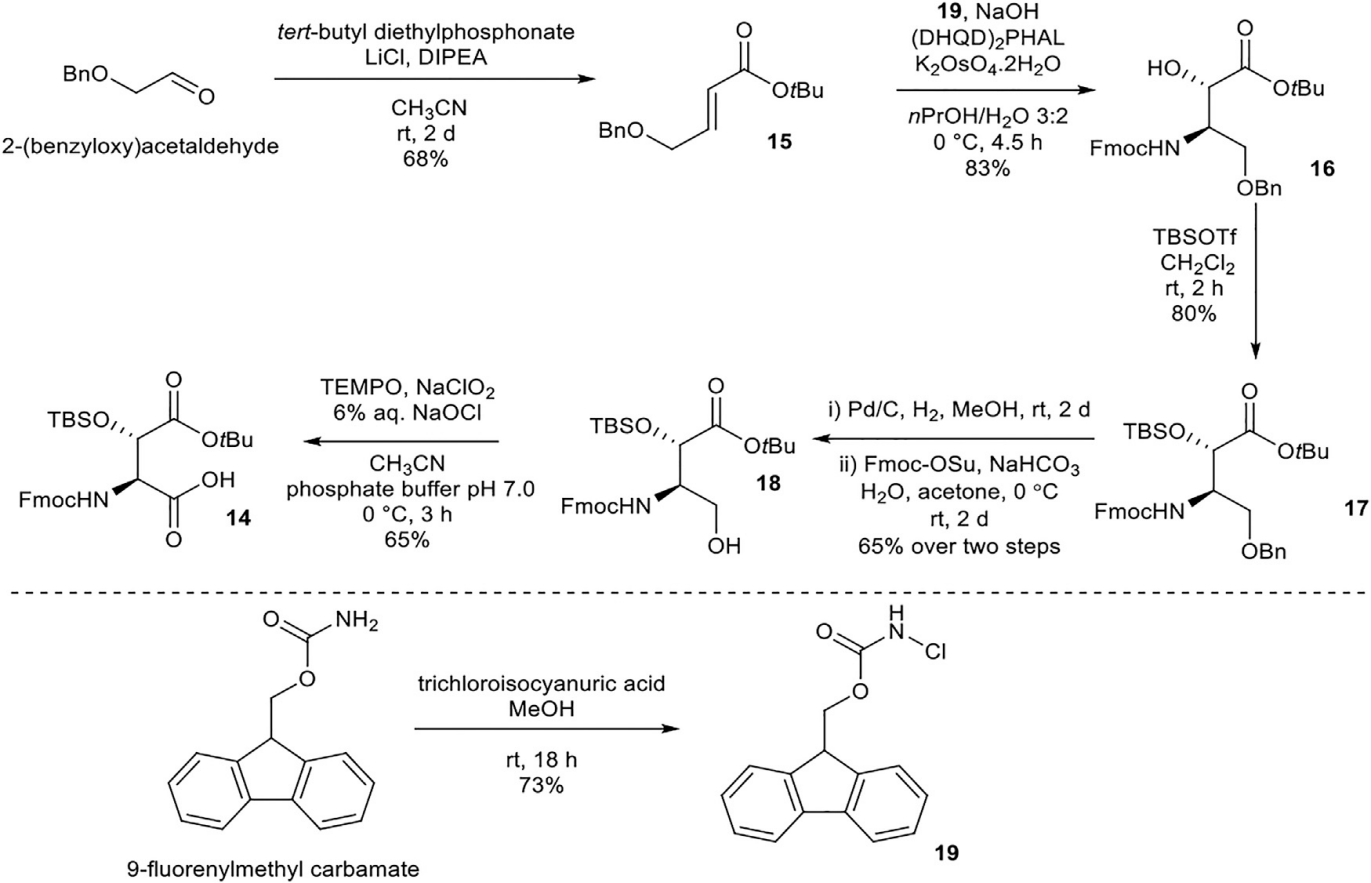
Synthesis of (2*S*,3*S*)-Fmoc-3-Hy(TBS)Asp(O*t*Bu)-OH (**14**) building block.

(2*S*,3*S*)-Fmoc-3-Hy(TBS)Asp(O*t*Bu)-OH (**14**) was prepared following literature precedent (Scheme 3) (Moreira and Taylor, 2018). α,β-Unsaturated ester **15** was synthesized from 2-(benzyloxy)acetaldehyde *via* a Horner–Wadsworth–Emmons reaction under Masamune–Roush conditions. Sharpless asymmetric aminohydroxylation using FmocNHCl (**19**, readily available from 9-fluorenylmethyl carbamate) (Gwon et al., 2015) as a nitrogen source afforded **16**. *tert*-Butyldimethylsilyl (TBS) protection of the hydroxyl group using TBSOTf yielded **17**. Attempted benzyl deprotection using Bobbit’s salt (4-acetamido-2,2,6,6-tetramethylpiperidine·BF_4_) returned only starting material, while deprotection using BCl_3_ and pentamethylbenzene resulted in concomitant loss of the *t*Bu ester. Hydrogenation of **17** removed the benzyl and Fmoc protecting groups concurrently, and the free amine was reprotected using Fmoc-OSu to afford **18**. Finally, oxidation of alcohol **18** using TEMPO/NaClO_2_/NaOCl afforded the desired building block **14**.

#### Synthesis of (2*S*,4*R*)-Fmoc-4-MePro-OH (20)

**SCHEME 4:**
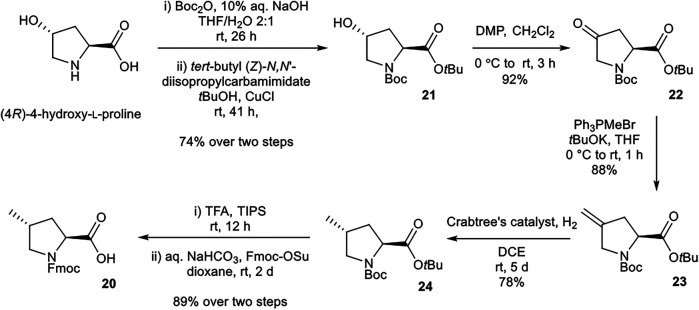
Synthesis of (2*S*,4*R*)-Fmoc-4-MePro-OH (**20**) building block.

(2*S*,4*R*)-Fmoc-4-MePro-OH (**20**) was prepared according to procedures adapted from Murphy *et al.* (Murphy et al., 2008) (Scheme 4). *N* ^α^-Boc protection and *t*Bu esterification of (4*R*)-4-hydroxy-l-proline afforded alcohol **21**. Dess-Martin periodinane oxidation of **21** to ketone **22**, followed by Wittig reaction with Ph_3_PMeBr afforded alkene **23**. Selective reduction of **23** using Crabtree’s catalyst installed the desired (4*R*) stereochemistry. Global deprotection and *N* ^*α*^-Fmoc protection afforded the desired building block **20.**

#### Synthesis of (2*E*,4*Z*)-8-methylnona-2,4-dienoic acid (25)

**SCHEME 5:**
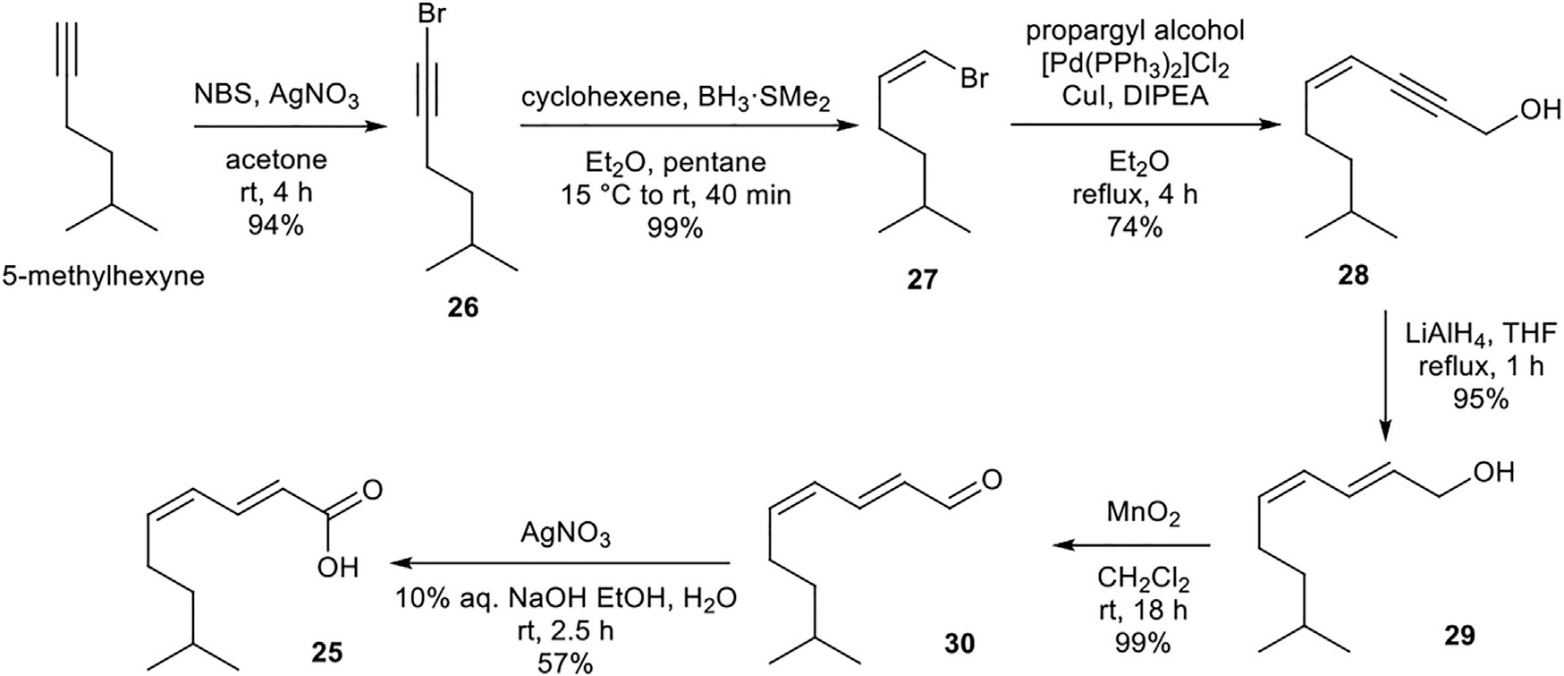
Synthesis of (2*E*,4*Z*)-8-methylnona-2,4-dienoic acid (**25**) building block.

The lipid building block **25** was prepared from readily available 5-methylhexyne (Scheme 5). Sequential bromination, boron-mediated reduction, and Sonogashira cross-coupling with propargyl alcohol provided enyne **28**. Alkyne reduction and two-step oxidation afforded desired (2*E*,4*Z*) polyunsaturated carboxylic acid **25**. The acidic nature of silica gel resulted in partial isomerization of the (4*Z*)-alkene upon attempted purification of **25**. Instead, purification and resulting undesired isomerization could be avoided by assuring complete purity of precursor **30** which upon oxidation yielded (2*E*,4*Z*)-**25** in excellent purity.

### Synthesis of Analogues of Malacidin A

**FIGURE 2:**
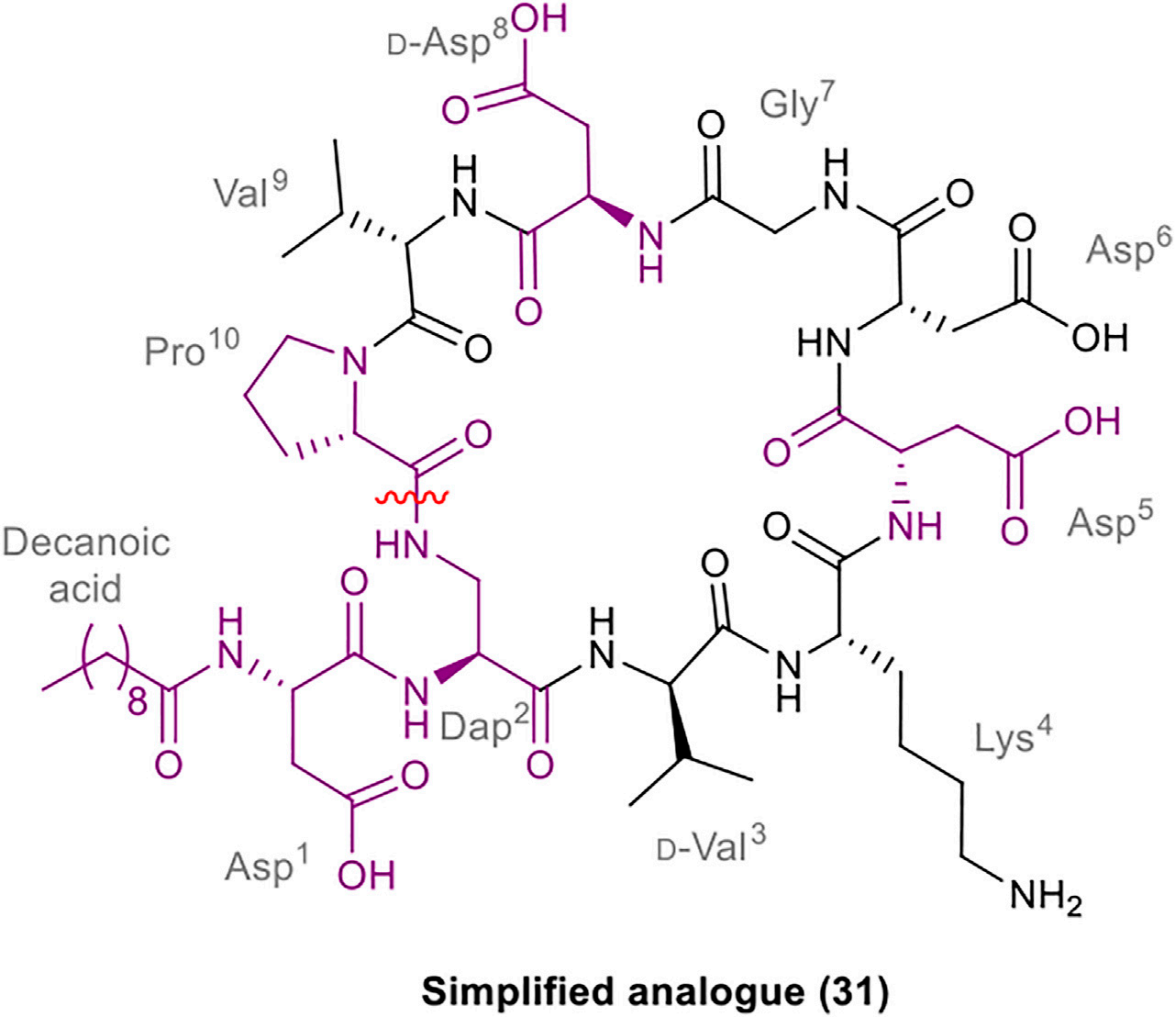
Structure of the simplified analogue of malacidin A (**31**); The substituted residues are highlighted in purple; the disconnection site is shown with a red wavy line.

**SCHEME 6:**
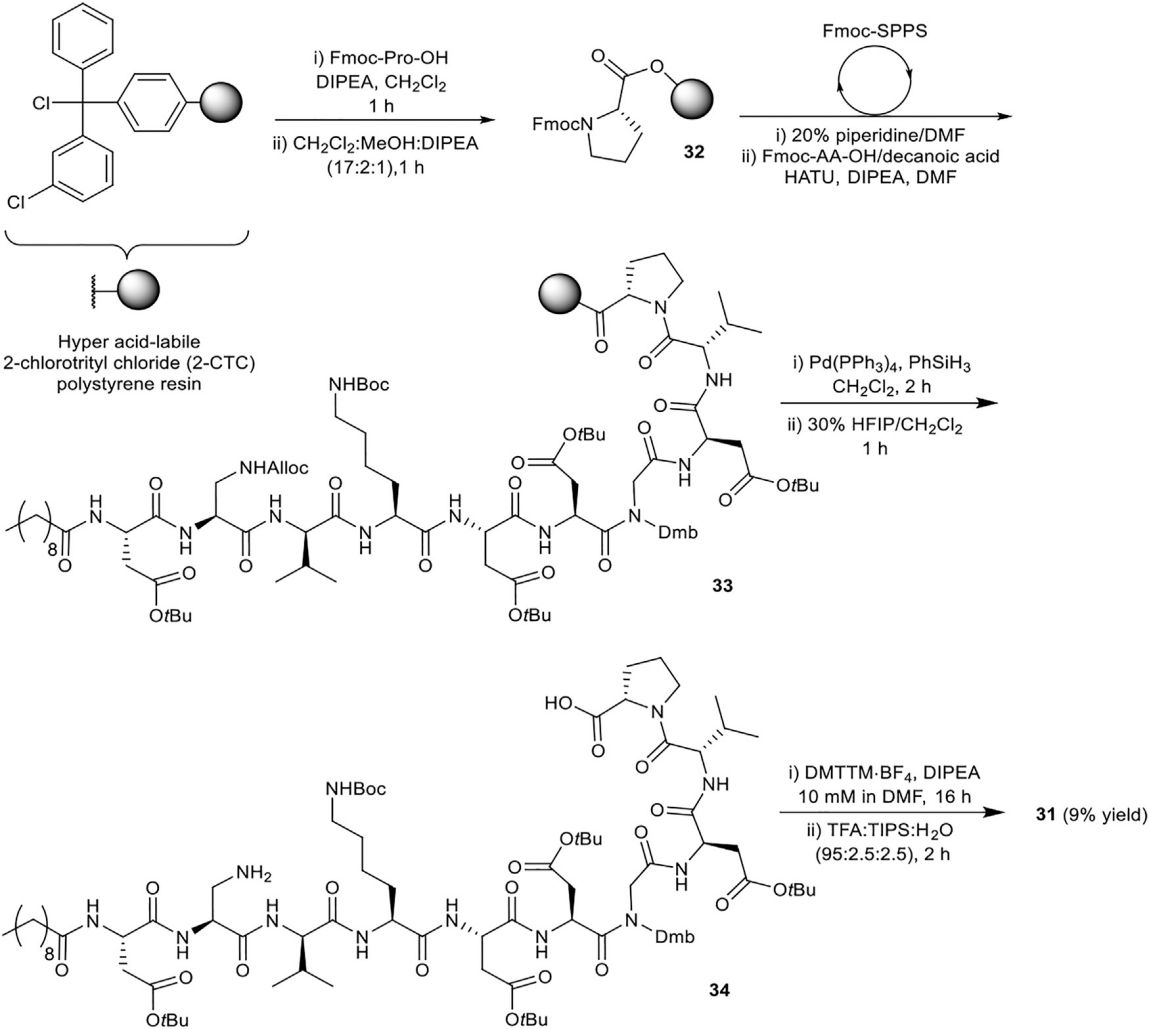
Synthesis of simplified analogue **31**.

A robust synthetic strategy toward diastereomer **1a** was established by first preparing a simplified analogue (**31**), wherein all non-canonical amino acids and the unsaturated lipid tail were substituted for the corresponding canonical/commercially available amino acids and decanoic acid, respectively (Figure 2). The synthetic route was inspired by the synthesis of glycinocins A-C reported by the Payne group (Corcilius et al., 2017). It was envisioned that macrocyclization would take place at the same site as the native peptide’s macrolactam bond, i.e. between the β-NH_2_ of the Dap^2^ derivative and α-CO_2_H of Pro^10^. The protected linear precursor was assembled *via* Fmoc-SPPS on a hyper acid-labile 2-chlorotrityl chloride (2-CTC) polystyrene (PS) resin (Scheme 6). The use of 2-CTC PS resin was also required to prevent diketopiperazine formation upon incorporation of Pro as the *C*-terminal residue. This synthetic approach required an orthogonally protected Dap^2^ building block, thus, commercially available Fmoc-Dap(Alloc)-OH was used. As the peptide sequence contains an aspartamide-prone Asp^6^Gly^7^ region, dimethoxybenzyl (Dmb)*N* ^α^-protected Gly^10^ was used.

The synthesis of **31** commenced with attachment of Fmoc-Pro-OH to 2-CTC resin to give resin-bound **32** (Scheme 6). After capping of unreacted chloride sites with methanol and *N*,*N*-diisopropylethylamine (DIPEA), the assembly of orthogonally protected linear precursor **33** was undertaken. Fmoc removal was achieved by treatment with 20% piperidine in *N*,*N*-dimethyl formamide (DMF) (*v/v*). l-Amino acids were coupled using 1-[bis(dimethylamino)methylene]-1*H*-1,2,3-triazolo[4,5-*b*]pyridinium 3-oxide hexafluorophosphate (HATU) and DIPEA in DMF for 30 min, and for 2 h when using d-amino acids, Dap^2^, Gly^7^ and decanoic acid. Double coupling was found to be necessary for Fmoc-Dap(Alloc)-OH. Next, Alloc removal from Dap^2^ was carried out using tetrakis(triphenylphosphine)palladium(0) (Pd(PPh_3_)_4_) and phenylsilane (PhSiH_3_). Next the assembled fully protected linear peptide was cleaved from the resin with 1,1,1,3,3,3-hexafluoroisopropanol (HFIP) in CH_2_Cl_2_ (3:7, *v/v*) for 1 h. Following solvent evaporation the peptidic residue was dissolved in H_2_O:CH_3_CN (1:4, *v/v*) and lyophilized. The obtained side chain protected linear precursor **34** was subjected to macrocyclization conditions (peptide concentration 10 mM) using 4-(4,6-dimethoxy-1,3,5-triazin-2-yl)-4-methylmorpholinium tetrafluoroborate (DMTTM·BF_4_) and DIPEA in DMF overnight. No difficulties were observed during the handling and dissolution of the maximally protected peptide. The final side chain deprotection was performed using trifluoroacetic acid (TFA), triisopropylsilane (TIPS), and H_2_O (95:2.5:2.5, *v/v/v*) for 2 h, followed by purification by reverse-phase high pressure liquid chromatography (RP-HPLC) to give simplified analogue **31** in 9% overall yield.

**SCHEME 7:**
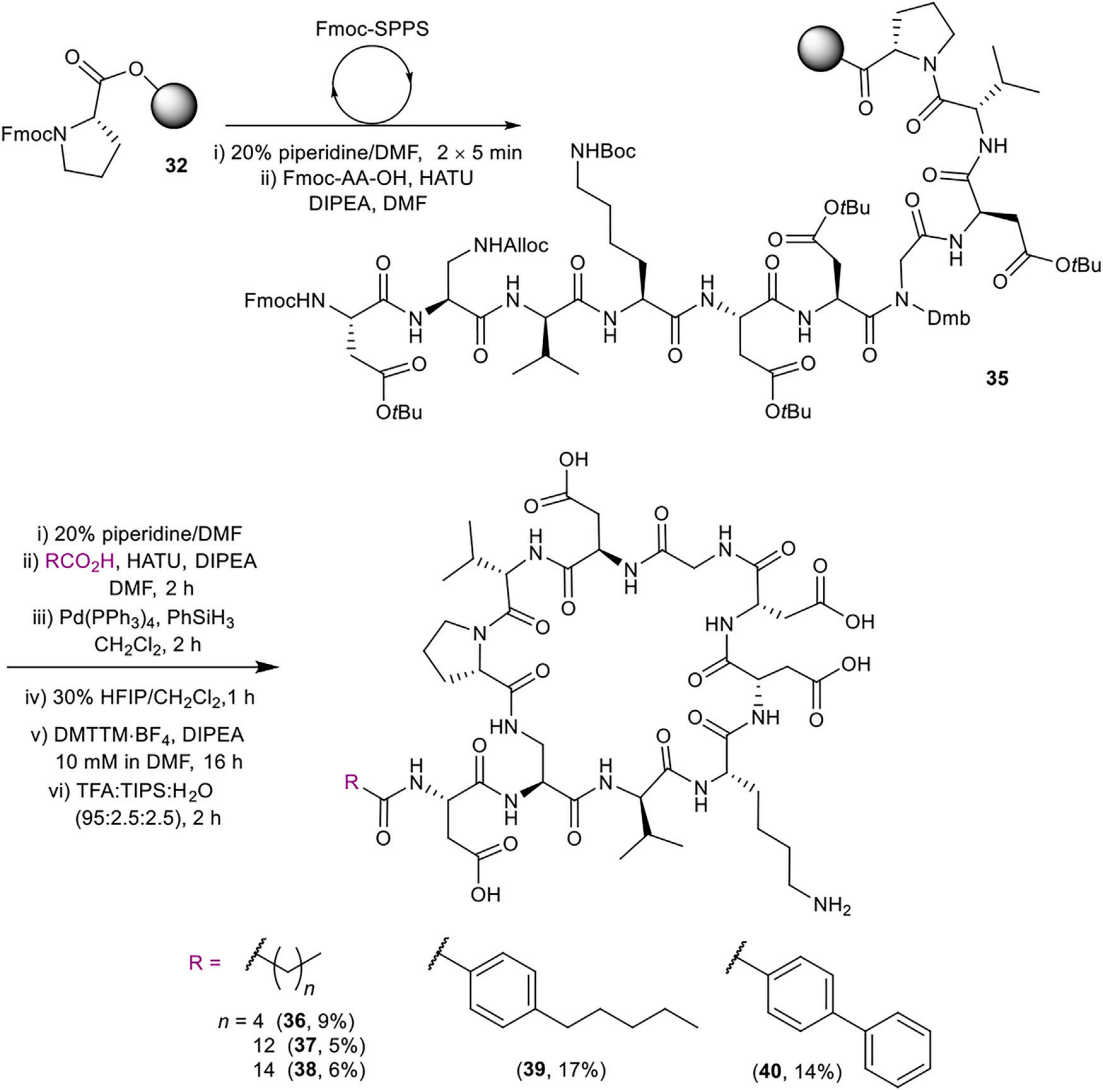
Synthesis of simplified analogues (**36–40**) with variation in the lipid moiety. Overall yields are given in brackets.

Late-stage incorporation of the lipid enabled the preparation of five additional lipid tail analogues based on the simplified peptidic core of **31**, namely hexanoyl, tetradecanoyl, hexadecanoyl, 4-pentylbenzoyl and 4-phenylbenzoyl analogues **36–40** (Scheme 7). The previously detailed SPPS protocol was used to prepare the common Fmoc protected linear sequence **35**, followed by division of the resin for coupling to the various lipid tails. The remaining steps of the synthesis for each analogue were carried out separately using the same conditions as for **31**. Analogues **36**–**40** were thus obtained in 5–17% yield in >96% purity after RP-HPLC purification.

Attention next focused on synthesis of the selected diastereomer of malacidin A **1a**. While the same overall strategy could be used, some modification of the synthesis was required to incorporate the (2*S*,3*S*)-Fmoc-3-MeDap(Dde)-OH (**8**), (2*S*,3*S*)-Fmoc-3-Hy(TBS)Asp(O*t*Bu)-OH (**14**) and (2*E*,4*Z*)-8-methylnona-2,4-dienoic acid (**25**) building blocks. The unsaturated nature of the lipid tail precluded use of Alloc as a protecting group on the 3-MeDap^2^ residue sidechain (Keinan and Greenspoon, 1986). (2*S*,3*S*)-Fmoc-3-MeDap(Dde)-OH (**8**) was therefore prepared, as the Dde group could be removed from the fully assembled linear precursor under mild conditions using hydroxylamine hydrochloride/imidazole without affecting the unsaturation of the lipid tail (Díaz-Mochón et al., 2004). Lipid stability investigations (see Supplementary Material Section 5) revealed that partial *cis-trans* isomerization of the (4Z)-π-bond of the lipid could be minimized by performing the final side chain deprotection in 50% TFA for 30 min. However, removal of the TBS protecting group was incomplete under these conditions and a separate deprotection step was required.

**SCHEME 8:**
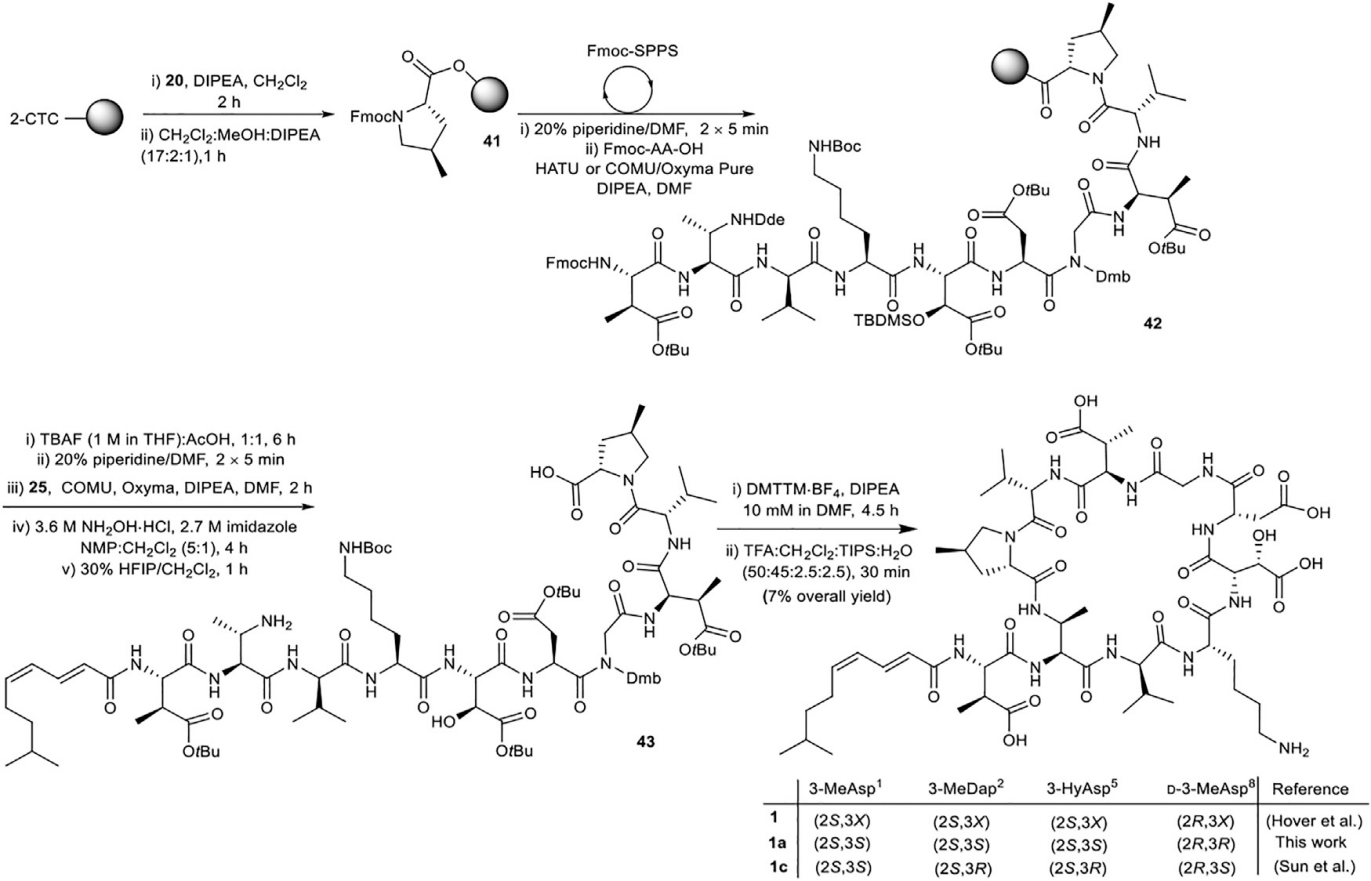
Synthesis of diastereomer of malacidin A, **1a**, employing chemically synthesized building blocks. *X* represents undefined β-carbon-stereochemistry in the isolated malacidin A (Hover et al., 2018), **1c** represents the correct stereochemistry of malacidin A (Sun et al., 2020).

The synthesis of malacidin analogue **1a** began with loading of (2*S*,4*R*)-Fmoc-4-MePro-OH (**20**) onto 2-CTC resin followed by iterative Fmoc-SPPS (Scheme 8). Commercially available amino acids were incorporated using the previously established conditions. Custom building blocks (**2**, **7**, **8**, **14**, **25**) were coupled using (1-cyano-2-ethoxy-2-oxoethylidenaminooxy)dimethylamino-morpholino-carbenium hexafluorophosphate (COMU)/ethyl cyano(hydroxyimino)acetate (Oxyma Pure)/DIPEA as the coupling reagents to maximize the coupling efficiency and reduce the number of molecular equivalents required for complete coupling (1.2 equiv.). Next, TBS removal from the side chain hydroxy group of 3-HyAsp^5^ residue using tetrabutylammonium fluoride (TBAF) buffered with AcOH (1:1, 15 eq.) was performed on peptidyl-resin **42** followed by Fmoc removal and coupling of the polyunsaturated lipid **25**. *N* ^β^ *-*Dde removal from the 3-MeDap^2^ residue was carried out under mild conditions using 3.6 M NH_2_OH·HCl and 2.7 M imidazole in NMP/CH_2_Cl_2_ (5:1, *v/v*) for 4 h. Following cleavage of the resulting peptide sequence from the resin with HFIP:CH_2_Cl_2_ (3:7, *v/v*) and solvent evaporation the crude peptide was dissolved in H_2_O:CH_3_CN (1:4, *v/v*) and lyophilized. The obtained protected linear peptide **43** was subjected to macrocyclization with DMTTM·BF_4_ and DIPEA at 10 mM dilution. As above, no difficulties were experienced during manipulations of the protected peptide. Gratifyingly, the reaction proceeded smoothly with complete consumption of the starting material in 4.5 h. Finally, side chain removal was carried out using the optimized deprotection cocktail of TFA:CH_2_Cl_2_:H_2_O:TIPS (50:45:2.5:2.5, *v/v/v/v*) for 30 min with no detectable isomerization of the polyunsaturated lipid. To reduce exposure of the acid-sensitive polyunsaturated lipid to highly acidic TFA during HPLC purification, an eluent system of H_2_O and CH_3_CN containing 0.1% formic acid was employed, providing **1a** in 7% overall yield.

Evaluation of the antibacterial activity of the synthesized analogues **1a**, **31** and **36**–**40** was then undertaken. Unfortunately, no activity toward *S. aureus* was observed for these analogues using media supplemented with 1.25–1.5 mM CaCl_2_, as recommended for biotesting of daptomycin (Wiegand et al., 2008) (Supplementary Tables S2, S3). As native malacidin A (**1**) showed maximum activity at 15 mM CaCl_2_ (Hover et al., 2018), diastereomer **1a** was also tested at this concentration but no activity was observed (Supplementary Table S2). This observation indicates that both the presence and absolute configuration of the β-substituents of the non-canonical amino acids are important for the activity of the antibiotic, most likely through their interaction with Ca^2+^ ions to form the active antibiotic-Ca^2+^ complex. This observation is not unusual, as previous reports of SAR studies of A54145 D and daptomycin CDLAs showed that removal or reversal of configuration at even one stereocenter may result in a significant reduction or complete loss of bioactivity (Kralt et al., 2019; Xu et al., 2019). It is likely that orientation of the β-OH of 3-HyAsp^5^, that forms part of the calcium binding motif, is highly important to provide efficient coordination to Ca^2+^ ions. Further SAR studies are required to assess the contribution of the individual non-canonical residues to the antimicrobial activity of malacidin A.

Comparison of the ^1^H and ^13^C NMR spectra of diastereomer **1a** to the reported spectra of malacidin A (under similar conditions, see **SI**), showed significant differences in the α-proton region (Hover et al., 2018) (Supplementary Figure S12). The ^1^H spectrum of daptomycin is known to change upon Ca^2+^ binding, demonstrating significant line broadening that is characteristic of aggregation (Ball et al., 2004). To investigate the Ca^2+^ binding capability of **1a**, the ^1^H NMR spectra of **1a** was recorded in the presence of CaCl_2_ at 1.5 mM and 15 mM (Supplementary Figure S16), concentrations similar to those used in MIC assays for daptomycin (typically 1.25 mM) and malacidin (**1**) respectively. Disappointingly, no detectable signal shifts or line broadening were observed (Supplementary Figure S14), indicating that **1a** fails to interact with Ca^2+^ ions, hence the lack of antibacterial activity. Despite these differences, it was observed that the lipid sp^2^ proton signals closely matched that of the natural product and no peaks arising from *cis-trans* isomerization were observed, indicating the tolerance of acid-sensitive lipid **25** to the optimized TFA side chain deprotection conditions and HPLC purification protocols.

In summary, seven novel analogues of malacidin A (**1**) were synthesized using primarily an Fmoc-SPPS-based strategy followed by late stage solution-phase macrolactamization and subsequent side chain deprotection. One diastereomeric analogue of the native sequence, **1a**, and six simplified analogues containing all canonical/commercially available amino acids with variations in the lipid tail (**31**, **36**–**40**) were obtained in good overall yields. Despite the lack of activity observed for these analogues, the concise and versatile synthetic strategy reported herein lays a foundation for further SAR studies of malacidin A. In contrast to the reported synthesis of malacidin A, the synthetic route described herein has improved yields, requires no additional amino acids bearing auxiliary groups to aid cyclization, and involves minimal solution-phase manipulations. The mostly solid-phase strategy also permits a single, final purification step. Additionally, a late stage incorporation of the lipid moiety on resin enables facile preparation of lipid analogues to probe the role of the lipid unsaturation and branching on antibacterial activity.

## Data Availability

The original contributions presented in the study are included in the article/Supplementary Material, further inquiries can be directed to the corresponding authors.
