# Supplementary material for: A Concise Synthetic Strategy Towards the Novel Calcium-dependent Lipopeptide Antibiotic, Malacidin A and Analogues
Source: Front Chem. 2021 Aug 4;9:687875. doi: 10.3389/fchem.2021.687875 (PMC8372822; doi:10.3389/fchem.2021.687875)
Supplement: Supplementary file 1 [file DataSheet1.docx]

Supplementary Material

Contents

[1 General information 2](#_Toc66696217)

[1.1 General information for synthesis of building blocks 2](#_Toc66696218)

[1.2 General information for peptide synthesis 3](#_Toc66696219)

[2 Synthesis of building blocks 6](#_Toc66696220)

[2.1 Synthesis of (2*S*,3*S*)-Fmoc-3-MeAsp(O*t*Bu)-OH (2) 6](#_Toc66696221)

[2.2 Synthesis of (2*R*,3*R*)-Fmoc-3-MeAsp(O*t*Bu)-OH (7) 10](#_Toc66696222)

[2.3 Synthesis of (2*S*,3*S*)-Fmoc-3-MeDap(Dde)-OH (8) 13](#_Toc66696223)

[2.4 Synthesis of (2*S*,3*S*)-Fmoc-3-Hy(TBS)Asp(O*t*Bu)-OH (14) 18](#_Toc66696224)

[2.5 Synthesis of (2*S*,4*R*)-Fmoc-3-MePro-OH (20) 22](#_Toc66696225)

[2.6 Synthesis of (2*S*,4*R*)-Fmoc-3-MePro-OH (25) 26](#_Toc66696226)

[3 General Procedures for Peptide Synthesis 31](#_Toc66696227)

[4 Experimental for synthesis of analogues of malacidin A 34](#_Toc66696228)

[4.1 Synthesis of simplified analogue 31 34](#_Toc66696229)

[4.2 Synthesis of the common peptide intermediate 35 35](#_Toc66696230)

[4.3 Synthesis of simplified analogue 36 36](#_Toc66696231)

[4.4 Synthesis of simplified analogue 37 38](#_Toc66696232)

[4.5 Synthesis of simplified analogue 38 39](#_Toc66696233)

[4.6 Synthesis of simplified analogue 39 40](#_Toc66696234)

[4.7 Synthesis of simplified analogue 40 42](#_Toc66696235)

[4.8 Synthesis of diastereomer 1a 43](#_Toc66696236)

[5 Stability evaluation to TFA of (2*E*,4*Z*)-8-methylnona-1,2-dienoic acid (25) bound to a model peptide. 45](#_Toc66696237)

[5.1 Synthesis and stability evaluation of (2*E*,4*Z*)-8-methylnona-1,2-dienoyl-Ala-Ala-OH (51) 45](#_Toc66696238)

[5.2 Synthesis of (2*E*,4*Z*)-8-methylnona-1,2-dienoyl-Ala-Lys-Asp-OH (53) and testing conditions for TFA-mediated cleavage/side chain deprotection 50](#_Toc66696239)

[6 Antibacterial susceptibility testing 52](#_Toc66696240)

[6.1 Minimum inhibitory concentration (MIC) assay of malacidin diastereomer 1a 52](#_Toc66696241)

[6.2 Minimum inhibitory concentration (MIC) assay of simplified malacidin analogues 31, 36-40 53](#_Toc66696242)

[7 1H and 13C NMR spectra of diastereomer 1a 54](#_Toc66696243)

[8 1H and 13C NMR spectra of building blocks 57](#_Toc66696244)

[9 References 88](#_Toc66696245)

# General information

## General information for synthesis of building blocks

Unless otherwise stated, all reactions were performed under an oxygen-free atmosphere of nitrogen or argon in oven dried glassware. Anhydrous solvents were either freshly distilled over drying agents or dried using an LC Technical SP-1 solvent purification system. Tetrahydrofuran (THF), diethyl ether (Et2O) and 1,4-dioxane were freshly distilled over sodium/benzophenone ketyl. Dichloromethane (CH2Cl2), acetonitrile (CH3CN), methanol (MeOH) and ethanol (EtOH) were freshly distilled from calcium hydride. Toluene was freshly distilled over sodium. Diisopropylethylamine and triethylamine were distilled from calcium hydride and stored over potassium hydroxide pellets under N2. *N,N*-Dimethylformamide (DMF) and dimethylsulfoxide (DMSO) were freshly distilled from molecular sieves (Linde type 4 Å). Commercially available starting materials and all other reagents were used as received unless otherwise noted.

Yields refer to chromatographically and spectroscopically (1H NMR) homogeneous materials, unless otherwise stated. Reactions performed at low temperature were cooled either with an acetone/dry ice bath to reach −78 °C, a brine/ice bath to reach −10 °C or a water/ice bath to reach 0 °C. Reactions were monitored by thin-layer chromatography (TLC) carried out on E. Merck silica gel plates using UV light as visualizing agent, and an ethanolic solution of vanillin or potassium permanganate solution and heat as developing agents. Kieselgel S 63-100 μm (Riedel-de-Hahn) silica gel was used for flash chromatography. Preparatory TLC was carried out on 500 μm, 20 × 20 cm UniplateTM (Analtech) silica gel thin layer chromatography plates.

NMR spectra were recorded at room temperature in CDCl3, CD3OD, DMSO-*d*6, or D2O solutions on either Bruker DRX400 spectrometers operating at 400 MHz for 1H nuclei and 100 MHz for 13C nuclei or using a Bruker Ascend 500 spectrometer operating at 500 MHz for 1H nuclei and 125 MHz for 13C nuclei. Chemical shifts were reported in parts per million (ppm) and were measured relative to the solvent in which the sample was analyzed (CDCl3: *δ*H 7.26 ppm or *δ*H 0.00 ppm (TMS), *δ*C 77.2 ppm; CD3OD: *δ*H 3.31 ppm, *δ*C 49.0 ppm; DMSO-*d*6: *δ*H 2.50 ppm, *δ*C 39.5 ppm; D2O: *δ*H 4.79 ppm). 1H NMR data were reported as position (*δ*), multiplicity (s = singlet, br s = broad singlet, d = doublet, dd = doublet of doublets, ddd = doublet of doublets of doublets, dt = doublet of triplets, t = triplet, td = triplet of doublets, q = quartet, ABq = AB quartet, p = pentet, m = multiplet), coupling constant (*J*, Hz), and relative integral. 13C NMR data were reported as position (*δ*). Where distinguishable from those due to a major isomer or diastereomer, resonances due to the minor isomer or diastereomer are denoted by an asterisk (*).

Melting points were determined on a Kofler hot-stage apparatus. Optical rotations were measured with an Autopol® IV automatic polarimeter, using the sodium-D line (589 nm), with the concentration measured in grams per 100 mL. Infrared (IR) spectra were recorded on a Perkin Elmer Spectrum 100 FT-IR spectrometer using a diamond ATR sampling accessory. High-resolution mass spectra (HRMS) were obtained using a VG70SE spectrometer or on a micrOTOF-Q II mass spectrometer.

## General information for peptide synthesis

All reagents were purchased as reagent grade and used without further purification. Solid phase peptide synthesis was carried out manually in a glass-fritted vessel at room temperature. Solvents for peptide synthesis and reverse phase high-pressure liquid chromatography (RP-HPLC) were purchased as synthesis grade and HPLC grade, respectively.

*O*-(7-Azabenzo-triazol-1-yl)-1,1,3,3*-*tetramethyluronium hexafluorophosphate (HATU), Fmoc-Pro-OH, Fmoc-Val-OH, Fmoc-Asp(O*t*Bu)-OH, Fmoc-D-Asp(O*t*Bu)-OH Fmoc-Lys(Boc)-OH, Fmoc-Dap(Alloc)-OH and 2-chlorotrityl chloride (2-CTC) polystyrene resin were purchased from ChemPep (Wellington, FL, USA). Fmoc-(Dmb)Gly-OH was purchased from AAPPTec. (Louisville, KY, USA). Fmoc-D-Val-OH, decanoic acid, 1-cyano-2-ethoxy-2-oxoethylidenaminooxy)dimethylamino-morpholino-carbenium hexafluorophosphate (COMU), tetrakis(triphenylphosphine) palladium(0) (Pd(PPh3)4) and 4-(4,6-dimethoxy-1,3,5-triazin-2-yl)-4-methylmorpholinium tetrafluoroborate (DMTMM.BF4) were purchased from AK Scientific (Union City, CA, USA). Dichloromethane (CH2Cl2), methanol (MeOH) and diethyl ether (Et2O) were purchased from ECP (Auckland, New Zealand). Trifluoroacetic acid (TFA) was purchased form Oakwood Chemicals (Estill, NC, USA). *N,N*-Diisopropylethylamine (DIPEA), piperidine, triisopropylsilane (TIPS), triphenylsilane (PhSiH3), hexanoic acid, myristic acid, palmitic acid, biphenyl-4-carboxylic acid, hydroxylamine hydrochloride (NH2OH.HCl), *N*-Methyl-2-pyrrolidone (NMP) and tetrabutylammonium fluoride (TBAF) (1M in THF) were purchased from Sigma-Aldrich (St. Louis, MO, USA). Ethyl cyano(hydroxyimino)acetate (Oxyma Pure) and imidazole were purchased from Merck (Darmstadt, Germany). Acetonitrile (CH3CN) (HPLC grade), *N,N*-dimethylformamide (DMF) (synthesis grade) were purchased from Thermo Fisher Scientific (Hampton, NH, USA). H2O was purified using a Sartorius (Göttingen, Germany) arium® pro ultrapure water system.

Dibenzofulvene measurements for the Fmoc-release assay was performed using a Shimadzu (Kyoto, Japan) UV-1280 spectrometer at 290 nm. Analytical RP-HPLC was performed on a Waters Alliance system using a Phenomenex (Torrance, CA, USA) Luna C18 column (5 μm, 110 Å, 4.60 × 250 mm) or Dionex (Sunnyvale, CA, USA) UltiMate 3000 system using a Phenomenex (Torrance, CA, USA) Gemini NX C18 column (5 μm, 110 Å, 4.60 × 250 mm). Buffer A: 0.1% (*v/v*) TFA in H2O; buffer B: 0.1% (*v/v*) TFA in CH3CN. Analytical liquid chromatography-mass spectrometry (LCMS) was performed on an Agilent (Santa Clara, CA, USA) 1260 Infinity equipped with an Agilent 6120 Quadrupole LC-MS using an Agilent Zorbax 300SB-C3 column (3.5 μm, 3.0 x 150 mm) with a linear gradient of 5-95% B (ca. 3% B/min) and a flow rate of 0.3 mL min-1. Buffer A: 0.1% (*v/v*) formic acid in H2O; buffer B: 0.1% (*v/v*) formic acid in CH3CN. Crude peptides were purified on Dionex UltiMate 3000 preparative HPLC. Buffer A: 0.1% (*v/v*) TFA or 0.1% (*v/v*) formic acid in H2O; buffer B: 0.1% (*v/v*) TFA or 0.1% (*v/v*) formic acid in CH3CN. Low resolution mass spectra were obtained using a Waters (Waltham, MA, USA) Quattro micro API Mass Spectrometer in electrospray ionisation (ESI) positive mode.

NMR spectra of **1a** (approx. 5.6 mM) were recorded at 298 K in D2O with Et3N(1.88 mM), in a Shigemi tube using a Bruker Ascend 500 spectrometer operating at 500 MHz for 1H nuclei and 125 MHz for 13C nuclei. In accordance with the reported spectra of isolated and synthetic malacidin A, the spectra were calibrated to the CH3 signal of Et3N, *δ*H 1.292 ppm and *δ*C 8.189 ppm1,2. The 1H spectrum was additionally recorded in the presence of either 1.5 mM or 15 mM CaCl2 to investigate calcium binding. The solvent signal was suppressed for all spectra using excitation sculpting.3

# Synthesis of building blocks

## Synthesis of (2*S*,3*S*)-Fmoc-3-MeAsp(O*t*Bu)-OH (2)

**1-Benzyl 4-(*tert*-butyl) *N,N*-dibenzyl-L-aspartate (3)**

To a stirred solution of (*S*)-2-amino-4-(*tert*-butoxy)-4-oxobutanoic acid (5.00 g, 26.4 mmol) in DMF (100 mL) and DMSO (20 mL) was added benzyl bromide (13.9 mL, 92.5 mmol) and K2CO3 (11.0 g, 79.3 mmol). The mixture was heated to 50 °C and stirred for 23 h. Another portion of benzyl bromide (4.0 mL, 26.3 mmol) was added and the reaction was stirred for an additional 24 h then cooled to room temperature. The slurry was filtered through Celite® eluting with EtOAc and concentrated *in vacuo*. The resulting residue was diluted with H2O (150 mL) and extracted with EtOAc (3 × 150 mL). The combined organic extracts were washed with brine (400 mL), dried over anhydrous Na2SO4, filtered and concentrated *in vacuo*. The resulting residue was redissolved in Et2O (150 mL) and washed with H2O (2 × 150 mL). The organic layer was then dried over anhydrous Na2SO4, filtered and concentrated *in vacuo*. The crude residue was purified by flash chromatography (hexanes-EtOAc 99:1 → 19:1) to afford *title compound* **3** (7.30 g, 60%) as a yellow oil. **Rf**  0.35 (hexanes-EtOAc 19:1); $\boldsymbol{\alpha}_{\boldsymbol{D}}^{\mathbf{21}}=$ ‒ ­­86.6 (*c* 1.05, CHCl3); **IR** νmax (neat): 3067, 3034, 2980, 2932, 1721, 1498, 1455 cm-1; **1H NMR** (400 MHz, CDCl3): *δ* 7.44 – 7.18 (m, 15H), 5.22 (ABq, ∆*δ*AB = 0.10, *J* = 12.2 Hz, 2H), 3.90 (dd, *J* = 6.5, 8.5 Hz, 1H), 3.66 (ABq, ∆δAB = 0.23, *J* = 13.9 Hz, 4H), 2.82 (m, 1H), 2.58 (m, 1H), 1.37 (s, 9H); **13C NMR** (100 MHz, CDCl3): *δ* 171.7, 170.3, 139.2, 136.1, 129.0, 128.7, 128.6, 128.4, 128.3, 127.2, 80.9, 66.5, 58.3, 55.0, 36.3, 28.1; **HRMS** *m/z* (ESI/Q-TOF) [M + H]+ calcd for C29H34NO4, 460.2482; found, 460.2489. The spectroscopic data were in agreement with those reported in the literature but no optical rotation was reported.4

**1-Benzyl 4-(*tert*-butyl) (2*S*)-2-(dibenzylamino)-3-methylsuccinate (4)**

To a stirred solution of protected amino acid **3** (5.0 g, 10.9 mmol) in THF (45 mL) was added KHMDS (0.72 M in THF, 18.1 mL, 13.0 mmol) at ‒78 °C. The reaction was stirred at ‒78 °C for 1 h, then MeI (2.4 mL, 38.1 mmol) was added dropwise. The mixture was allowed to warm to between ‒30 °C and ‒20 °C then stirred for 2.5 h. The reaction was quenched with sat. aq. NH4Cl (50 mL), stirred for 30 min, then extracted with EtOAc (3 × 50 mL). The combined organic extracts were washed with brine (100 mL), dried over anhydrous Na2SO4, filtered and concentrated *in vacuo*. The crude reside was purified by flash chromatography (hexanes-EtOAc 49:1) to afford *title compound* **4** (4.27 g, 83%) as a colorless oil as a 1:1 mixture of diastereomers. **Rf** 0.74 (hexanes-EtOAc 19:1); **IR** νmax (neat): 3434, 3233, 2980, 2930, 1725, 1650, 1568 cm-1; **1H NMR** (400 MHz, CDCl3): *δ* 7.48 – 7.20 (m, 15H٭), 5.30 – 5.17 (m, 2H٭), 3.98 (d, *J* = 13.6 Hz, 1H*), 3.82 (d, *J* = 13.4 Hz, 1H), 3.62 (d, *J* = 11.1 Hz, 0.5H*), 3.44 (d, *J* = 10.9 Hz, 0.5H), 3.35 – 3.27 (m, 2H٭), 3.00 (dq, *J* = 6.9, 11.2 Hz, 0.5H*), 2.87 (dq, *J* = 11.1, 7.2 Hz, 0.5H), 1.45 (s, 4.5H), 1.36 (s, 4.5H*), 1.14 (d, *J* = 7.35 Hz, 1.5H), 1.00 (d, *J* = 7.5 Hz, 1.5H*); **13C NMR** (100 MHz, CDCl3): *δ* 174.7, 173.1*, 171.3, 170.3*, 138.95, 138.93, 136.2, 135.9, 129.5, 129.3, 128.84, 128.75, 128.7, 128.6, 128.4, 128.3, 128.1, 127.2, 127.1, 80.55٭, 80.50*, 66.2, 64.7*, 62.7, 55.1*, 54.9, 41.6*, 40.2, 28.1*, 28.0, 15.4, 15.1*; *denotes peaks of one diastereomer, ٭denotes peaks arising from both diastereomers. **HRMS** *m/z* (ESI/Q-TOF) [M + Na]+ calcd for C30H35NNaO4, 496.2458; found, 496.2457.

**(2*S*,3*S*)-2-Amino-4-(*tert*-butoxy)-3-methyl-4-oxobutanoic acid (5)** and **(2*S*,3*R*)-2-amino-4-(*tert*-butoxy)-3-methyl-4-oxobutanoic acid (6)**

A mixture of amino acid **4** (4.0 g, 8.45 mmol) and 10% *w/w* Pd/C (0.90 g, 8.45 mmol) in degassed MeOH (170 mL) was stirred under an atmosphere of H2 (balloon) at room temperature for 17 h. The mixture was filtered through Celite® and concentrated *in vacuo*. The crude residue was purified by flash chromatography (EtOAc-*i*PrOH-H2O 8:2:1) to give *title compound* **5** (0.75 g, 43%) and *title compound* **6** (0.42 g, 24%) as white solids.

Characterization of (2*S*,3*S*)-2-amino-4-(*tert*-butoxy)-3-methyl-4-oxobutanoic acid (**5**): **Rf** 0.12 (CH2Cl2-MeOH 19:1 with trace AcOH); $\boldsymbol{\alpha}_{\boldsymbol{D}}^{\mathbf{21}}=$ ‒ ­­7.4 (*c* 1.03, Dioxane-H2O 1:1) (lit.5 $\boldsymbol{\alpha}_{\boldsymbol{D}}^{\mathbf{23}}=$ ‒ ­­7.0 (*c* 1.00, Dioxane-H2O 1:1)); **IR** νmax (neat): 3440, 3222, 3182, 2980, 2932, 2367, 1727, 1652, 1570 cm-1; **1H NMR** (400 MHz, D2O): *δ* 4.02 – 4.00 (m, 1H), 3.14 – 3.07 (m, 1H), 1.50 (s, 9H), 1.27 (d, *J* = 7.7 Hz, 3H); **13C NMR** (100 MHz, D2O): *δ* 176.3*, 174.4, 172.5, 172.2*, 83.9, 83.6*, 60.6*, 55.8, 44.1*, 40.7, 27.3, 27.1*, 22.7*, 20.5*, 12.3; *denotes signals arising from minor rotamers; **HRMS** *m/z* (ESI/Q-TOF) [M + H]+ calcd for C9H18NO4, 204.1230; found, 204.1231.

Characterization of (2*S*,3*R*)-2-amino-4-(*tert*-butoxy)-3-methyl-4-oxobutanoic acid (**6**): **Rf** 0.10 (CH2Cl2-MeOH 19:1 with trace AcOH); $\boldsymbol{\alpha}_{\boldsymbol{D}}^{\mathbf{21}}=$ + ­­17.2 (*c* 1.08, Dioxane-H2O 1:1) (lit.5 $\boldsymbol{\alpha}_{\boldsymbol{D}}^{\mathbf{23}}=$ + 13.0 (*c* 1.00, Dioxane-H2O 1:1)); **IR** νmax (neat): 3303, 2982, 2525, 1734, 1634, 1577, 1523 cm-1; **1H NMR** (400 MHz, D2O): *δ* 3.94 (d, *J* = 4.3 Hz, 1H), 3.20 – 3.14 (m, 1H), 1.48 (s, 9H), 1.30 (d, *J* = 7.3 Hz, 3H);; **13C NMR** (100 MHz, D2O): *δ* 173.9, 172.3, 83.8, 56.3, 40.4, 27.1, 12.7; HRMS *m/z* (ESI/Q-TOF) [M + H]+ calcd for C9H18NO4, 204.1230; found, 204.1228.

The spectroscopic data of **5** and **6** were in agreement with those reported in the literature.5

**(2*S*,3*S*)-2-((((9*H*-Fluoren-9-yl)methoxy)carbonyl)amino)-4-(*tert*-butoxy)-3-methyl-4-oxobutanoic acid (2)**

To a stirred solution of amino acid **5** (0.40 g, 2.0 mmol) in THF (13.3 mL) was added sat. aq. NaHCO3 (6.6 mL) and Fmoc-OSu (0.70 g, 2.1 mmol) and the mixture was stirred at room temperature for 23 h. The reaction was poured over H2O (50 mL) and extracted with EtOAc (3 × 50 mL). The aqueous layer was acidified to pH 4 using 1 M aq. HCl then further extracted with EtOAc (3 × 50 mL). The combined organic extracts were dried over anhydrous Na2SO4, filtered and concentrated *in vacuo*. The crude residue was purified by flash chromatography (hexanes-EtOAc 4:1 with 0.1% AcOH) to afford *title compound* **2** (0.35 g, 42%) as a white fluffy solid. **Rf**  0.20 (CH2Cl2-MeOH 9:1); $\boldsymbol{\alpha}_{\boldsymbol{D}}^{\mathbf{21}}=$ + 19.2 (*c* 1.00, CHCl3); **IR** νmax (neat): 3405, 3282, 2980, 2936, 1725, 1695 cm-1; **1H NMR** (400 MHz, CDCl3): *δ* 7.75 (d, *J* = 7.6 Hz, 2H), 7.60 – 7.57 (m, 2H), 7.39 (dd, *J* = 7.5, 7.5 Hz, 2H), 7.32 – 7.28 (m, 2H), 5.60 (d, *J* = 9.0 Hz, 1H), 4.70 (dd, *J* = 4.7, 9.0 Hz, 1H), 4.40 (d, *J* = 7.1 Hz, 2H), 4.23 (t, *J* = 7.1 Hz, 1H), 2.99 – 2.93 (m, 1H), 1.44 (s, 9H), 1.26 (d, *J* = 7.2 Hz, 3H); **13C NMR** (100 MHz, CDCl3): *δ* 175.3, 172.3, 156.2, 143.8, 141.4, 127.9, 127.2, 125.2, 120.1, 82.0, 67.5, 55.8, 47.2, 42.9, 28.0, 13.2; **HRMS** *m/z* (ESI/Q-TOF) [M + Na]+ calcd for C24H27NaNO6, 448.1731; found, 448.1724. The spectroscopic data were in agreement with those reported in the literature but no optical rotation was reported.2

## Synthesis of (2*R*,3*R*)-Fmoc-3-MeAsp(O*t*Bu)-OH (7)

**1-Benzyl 4-(*tert*-butyl) *N*,*N*-dibenzyl-*D*-aspartate (44)**

To a stirred solution of (*R*)-2-amino-4-(*tert*-butoxy)-4-oxobutanoic acid (8.00 g, 42.3 mmol) in DMF (80 mL) and DMSO (20 mL) was added benzyl bromide (22 mL, 148 mmol) and K2CO3 (17.5 g, 127 mmol). The mixture was heated to 50 °C and stirred for 17 h. Another portion of benzyl bromide (9.5 mL, 63.4 mmol) was added and the reaction was stirred for an additional 3 h then cooled to room temperature. The slurry was filtered through Celite® eluting with EtOAc and concentrated *in vacuo*. The resulting residue was diluted with H2O (150 mL), extracted with EtOAc (3 × 150 mL) and washed with brine (400 mL). The combined organic extracts were dried over anhydrous Na2SO4, filtered and concentrated *in vacuo*. The resulting residue was redissolved in Et2O (150 mL) and washed with H2O (2 × 150 mL). The organic layer was dried over anhydrous Na2SO4, filtered and concentrated *in vacuo*. The crude residue was purified by flash chromatography (hexanes-EtOAc 99:1 → 19:1) to afford *title compound* **44** (12.3 g, 63%) as a yellow oil. **Rf**  0.32 (hexanes-EtOAc 19:1); $\boldsymbol{\alpha}_{\boldsymbol{D}}^{\mathbf{20}}=$ + ­79.4 (*c* 1.07, CHCl3); **IR νmax** (neat): 3065, 3030, 2978, 2933, 2845, 1726, 1603 cm-1; **1H NMR** (400 MHz, CDCl3): *δ* 7.43 – 7.18 (m, 15H), 5.28 (ABq, ∆*δ*AB = 0.10, *J* = 12.1 Hz, 2H), 3.90 (dd, *J* = 6.5, 8.5 Hz, 1H), 3.78 (d, *J* = 13.8 Hz, 2H), 3.54 (d, *J* = 13.8 Hz, 2H), 2.85 – 2.79 (m, 1H), 2.58 (m, 1H), 1.37 (s, 9H); **13C NMR** (100 MHz, CDCl3): *δ* 171.7, 170.3, 139.2, 136.1, 129.0, 128.7, 128.6, 128.5, 128.3, 127.2, 80.9, 66.5, 58.3, 55.0, 36.3, 28.1; **HRMS** *m/z* (ESI/Q-TOF) [M + H]+ calcd for C29H34NO4, 460.2482; found, 460.2472.

**1-Benzyl 4-(*tert*-butyl) (2*R*)-2-(dibenzylamino)-3-methylsuccinate (45)**

To a stirred solution of protected amino acid **44** (7.0 g, 15.2 mmol) in THF (70 mL) was added KHMDS (0.95 M in THF, 19.2 mL, 18.3 mmol) at ‒78 °C. The reaction was stirred at ‒78 °C for 1 h, then MeI (3.3 mL, 53.3 mmol) was added to the mixture dropwise. The mixture was allowed to warm between ‒30 °C and ‒20 °C then stirred for 3.5 h. The reaction was quenched with sat. aq. NH4Cl (50 mL), stirred for 30 min, then extracted with EtOAc (3 × 50 mL). The combined organic extracts were washed with brine (100 mL), dried over anhydrous Na2SO4, filtered and concentrated *in vacuo*. The crude reside was purified by flash chromatography (hexanes-EtOAc 99:1→ 49:1) to afford *title compound* **45** (6.51 g, 90%) as a colorless oil as a 1:1 mixture of diastereomers. **Rf**  0.41 (hexanes/EtOAc 19:1); **IR νmax** (neat): 3066, 3031, 2976, 2936, 2841, 1725, 1603 cm-1; **1H NMR** (400 MHz, CDCl3): *δ* 7.48 – 7.19 (m, 15H٭), 5.30 – 5.16 (m, 2H٭), 3.98 (d, *J* = 13.6 Hz, 1H*), 3.82 (d, *J* = 13.6 Hz, 1H), 3.62 (d, *J* = 11.1 Hz, 0.5H*), 3.45 (d, *J* = 11.3 Hz, 0.5H), 3.35 – 3.27 (m, 2H٭), 3.01 (dq, *J* = 6.7, 11.1 Hz, 0.5H*), 2.88 (dq, *J* = 7.2, 11.3 Hz, 0.5H), 1.41 (s, 4.5H), 1.32 (s, 4.5H*), 1.15 (d, *J* = 7.2 Hz, 1.5H), 1.00 (d, *J* = 7.0 Hz, 1.5H)*; **13C NMR** (100 MHz, CDCl3): *δ* 174.7, 173.1*, 171.3, 170.4*, 139.0, 138.9, 136.2, 135.9, 129.5, 129.3, 128.9, 128.77, 128.7, 128.6, 128.41, 128.36, 128.1, 127.2, 127.1, 80.55, 80.50*, 66.2٭, 64.7*, 62.7, 55.1*, 54.9, 41.6*, 40.2, 28.1*, 28.0, 15.4, 15.1*; *denotes peaks of one diastereomer,٭denotes peaks arising from both diastereomers; **HRMS** *m/z* (ESI/Q-TOF) [M + H]+ calcd for C29H34NO4, 496.2458; found, 496.2461.

**(2*R*,3*R*)-2-Amino-4-(*tert*-butoxy)-3-methyl-4-oxobutanoic acid (46)** and **(2*R*,3*S*)-2-amino-4-(*tert*-butoxy)-3-methyl-4-oxobutanoic acid (47)**

A mixture of amino acid **45** (4.0 g, 8.45 mmol) and 10% *w/w* Pd/C (0.90 g, 8.45 mmol) in degassed MeOH (170 mL) was stirred under an atmosphere of H2 (balloon) at room temperature for 17 h. The mixture was filtered through Celite® and concentrated *in vacuo*. The crude residue was purified by flash chromatography (EtOAc-iPrOH-H2O 8:2:1) to afford *title compound* **46** (0.76 g, 44%) and *title compound* **47** (0.77 g, 45%) as white solids.

Characterization of (2*R*,3*R*)-2-amino-4-(*tert*-butoxy)-3-methyl-4-oxobutanoic acid (**46**): **Rf** 0.12 (CH2Cl2-MeOH 19:1 with trace AcOH); $\boldsymbol{\alpha}_{\boldsymbol{D}}^{20}=$ + ­6.6 (*c* 1.00, Dioxane-H2O 1:1); **IR** νmax (neat): 3435, 3231, 2981, 2930, 2367, 1726, 1651, 1567 cm-1; **1H NMR** (400 MHz, D2O): *δ* 4.02 – 4.00 (m, 1H), 3.14 – 3.07 (m, 1H), 1.50 (s, 9H), 1.27 (d, *J* = 7.7 Hz, 3H); **13C NMR** (100 MHz, D2O): *δ* 176.3*. 174.4, 172.5, 172.2*, 83.9, 83.6* 55.9, 44.1*, 40.8, 27.3, 27.1*, 22.7*, 22.7*, 12.0; *denotes signals arising from minor rotamers; **HRMS** *m/z* (ESI/Q-TOF) [M + H]+ calcd for C9H18NO4, 204.1230; found, 204.1234.

Characterization of (2*R*,3*S*)-2-amino-4-(*tert*-butoxy)-3-methyl-4-oxobutanoic acid (**47**): **Rf**  0.10 (CH2Cl2-MeOH 95:5 with trace AcOH); $\boldsymbol{\alpha}_{\boldsymbol{D}}^{\mathbf{20}}=$– 17.0 (*c* 1.00, Dioxane-H2O 1:1); **IR** νmax (neat): 3253, 2978, 2932, 1733, 1630, 1577, 1592 cm-1; **1H NMR** (400 MHz, D2O): *δ* 3.94 (d, *J* = 4.3 Hz, 1H), 3.21 – 3.14 (m, 1H), 1.48 (s, 9H), 1.30 (d, *J* = 7.3 Hz, 3H); **13C NMR** (100 MHz, D2O): *δ* 173.9, 172.3, 83.9, 56.3, 40.4, 27.1, 12.7; **HRMS** *m/z* (ESI/Q-TOF) [M + H]+ calcd for C9H18NO4, 204.1230; found, 204.1231.

The spectroscopic data were in agreement with those reported in the literature but no optical rotation was reported.2

**(2*R*,3*R*)-2-((((9*H*-Fluoren-9-yl)methoxy)carbonyl)amino)-4-(*tert*-butoxy)-3-methyl-4-oxobutanoic acid (7)**

To a stirred solution of amino acid **47** (0.45 g, 2.21 mmol) in THF (13.3 mL) was added sat. aq. NaHCO3 (7.4 mL) and Fmoc-OSu (0.78 g, 2.32 mmol) and the mixture was stirred at room temperature for 22 h. The reaction was poured over H2O (50 mL) and extracted with EtOAc (3 × 50 mL). The aqueous layer was acidified to pH 4 using 1 M aq. HCl then further extracted with EtOAc (3 × 50mL). The combined organic extracts were dried over anhydrous Na2SO4, filtered and concentrated *in vacuo*. The crude residue was purified by flash chromatography (hexanes-EtOAc 4:1 with 0.1% AcOH) to afford *title compound* **7** (0.76 g, 81%) as a white fluffy solid. **Rf**  0.69 (hexanes-EtOAc 3:7 with trace AcOH); $\boldsymbol{\alpha}_{\boldsymbol{D}}^{\mathbf{22}}=$‒ 17.2 (*c* 1.00, CHCl3); **IR** νmax (neat): 3254, 3007, 2979, 2930, 2010, 1734, 1710, 1630 cm-1; **1H NMR** (400 MHz, CDCl3): *δ* 7.76 (d, *J* = 7.6 Hz, 2H), 7.60 – 7.57 (m, 2H), 7.39 (dd, *J* = 7.5, 7.5 Hz, 2H), 7.32 – 7.28 (m, 2H), 5.59 (d, *J* = 9.0 Hz, 1H), 4.71 (dd, *J* = 4.7, 9.0 Hz, 1H), 4.40 (d, *J* = 7.1 Hz, 2H), 4.23 (t, *J* = 7.1 Hz, 1H), 3.00 – 2.93 (m, 1H), 1.44 (s, 9H), 1.26 (d, *J* = 7.2 Hz, 3H); **13C NMR** (100 MHz, CDCl3): *δ* 175.6, 172.3, 156.2, 143.8, 141.4, 127.9, 127.2, 125.2, 120.1, 82.0, 67.5, 55.8, 47.2, 43.0, 28.0, 13.2; **HRMS** *m/z* (ESI/Q-TOF) [M + Na]+ calcd for C24H27NaNO6, 448.1731; found, 448.1722.

## Synthesis of (2*S*,3*S*)-Fmoc-3-MeDap(Dde)-OH (8)

**Benzyl (*tert*-butoxycarbonyl)-*L*-threoninate (9)**

To a stirred solution of L-threonine (10.0 g, 84.0 mmol) in H­2O (200 mL) was added NaOH (10.1 g, 252 mmol) followed by Boc2O (44.0 g, 202 mmol) in THF (100 mL). The reaction was stirred at room temperature for 21 h then concentrated *in vacuo* to remove the THF. The resulting aqueous solution was washed with Et2O (250 mL) to remove excess Boc2O, then acidified to pH 2 with 1 M aq. H2SO4 and extracted with EtOAc (5 × 200 mL). The combined organic extracts were washed with brine (500 mL), dried over anhydrous Na­2SO4, filtered and concentrated *in vacuo* to afford crude (*tert*-butoxycarbonyl)-*L*-threonine, which was submitted directly to the next step without further purification.

To a stirred solution of crude (*tert*-butoxycarbonyl)-L-threonine (18.4 g, 83.9 mmol) in DMF(560 mL) at 0 °C was added Cs2CO3 (32.8 g, 101 mmol) and benzyl bromide (12.0 mL, 101 mmol). The mixture was allowed to warm to room temperature and stirred for 18 h, then diluted with H2O (500 mL) and extracted with EtOAc (3 × 300 mL). The combined organic extracts were dried over anhydrous Na2SO4, filtered and concentrated *in vacuo*. The crude residue was purified by flash chromatography (hexanes-EtOAc 9:1 → 7:3) to afford *title compound* **9** (22.5 g, 87% over two steps) as a colorless oil. **Rf**  0.14 (hexanes-EtOAc 3:2); $\boldsymbol{\alpha}_{\boldsymbol{D}}^{\mathbf{21}}=$ ‒ ­­13.9 (*c* 1.03, CHCl3) (lit.6 $\boldsymbol{\alpha}_{\boldsymbol{D}}^{\mathbf{20}}=$ ‒ 14.4 (*c* 1.05, CHCl3)); **IR** νmax (neat): 3473, 3349, 2979, 2969, 1709, 1685, 1520, 1501 cm-1; **1H NMR** (400 MHz, CDCl3): *δ* 7.37 – 7.35 (m, 5H), 5.29 – 5.17 (m, 3H), 4.31 – 4.29 (m, 2H), 1.90 – 1.81 (m, 1H), 1.45 (s, 9H), 1.24 (d, *J* = 6.4 Hz, 3H); **13C NMR** (100 MHz, CDCl3): *δ* 171.5, 156.3, 135.4, 128.7, 128.5, 128.3, 80.2, 68.2, 67.3, 59.0, 28.4, 20.1; **HRMS** *m/z* (ESI/Q-TOF) [M + Na]+ calcd for C16H23NaNO5, 332.1468; found, 332.1474. The spectroscopic data were in agreement with those reported in the literature.6

**Benzyl *N*-(*tert*-butoxycarbonyl)-*O*-tosyl-L-threoninate (10)**

To a stirred solution of ester **9** (12.0 g, 38.8 mmol) in pyridine (190 mL) at 0 °C was added Ts2O
(16.5 g, 50.4 mmol) in 3 portions over 5 min. The reaction was stirred at 0 °C for 1 h then allowed to warm to room temperature and stirred for 16 h. The pyridine was removed by a stream of N2 and the remaining oil was resuspended in EtOAc (300 mL), washed with 0.2 M aq. HCl (5 × 200 mL) and brine (200 mL). The organic layer was dried over anhydrous Na2SO4, filtered and concentrated *in vacuo*. The crude residue was purified by flash chromatography (hexanes-EtOAc 7:3) to afford *title compound* **10** (12.6 g, 70%) as a white solid. **Rf**  0.43 (hexanes-EtOAc 4:1); $\boldsymbol{\alpha}_{\boldsymbol{D}}^{\mathbf{20}}=$ + 44.6(*c* 1.00, CHCl3) (lit.7 $\boldsymbol{\alpha}_{\boldsymbol{D}}^{\mathbf{23}}=$ + 40.3(*c* 1.70, CHCl3)); **IR** νmax (neat): 3413, 3336, 2978, 2931, 1751, 1719, 1695, 1510, 1499 cm-1; **1H NMR** (400 MHz, CDCl3): *δ* 7.74 – 7.72 (m, 2H), 7.40 – 7.29 (m, 7H), 5.25 (d, *J* = 9.4 Hz, 1H), 5.19 (dq, *J* = 1.8, 6.5 Hz, 1H), 5.11 (d, *J* = 12.1 Hz, 1H), 4.89 (d, *J* = 12.1 Hz, 1H), 4.44 (dd, *J* = 1.7, 9.7 Hz, 1H), 2.42 (s, 3H), 1.43 (s, 9H), 1.33 (d, *J* = 6.5 Hz, 3H); **13C NMR** (100 MHz, CDCl3): *δ* 169.1, 155.8, 145.1, 135.0, 133.8, 129.9, 128.7, 128.6, 128.5, 128.0, 80.5, 78.7, 67.9, 57.8, 28.3, 21.7, 18.2; **HRMS** *m/z* (ESI/Q-TOF) [M + Na]+ calcd for C23H29NNaO7S, 486.1557; found, 486.1569. The spectroscopic data were in agreement with those reported in the literature 7

**Benzyl (2*S*,3*S*)-3-azido-2-((*tert*-butoxycarbonyl)amino)butanoate (11)**

To a stirred solution of tosylate **10** (8.0 g, 17.3 mmol) in DMF (120 mL) was added NaN3 (3.70 g, 57.0 mmol) in 3 portions over 10 min. The reaction was stirred at room temperature for 2 days then diluted with H2O (200 mL) and extracted with EtOAc (3 × 250 mL). The combined organic extracts were dried over anhydrous Na2SO4, filtered and concentrated *in vacuo*. The crude residue was purified by flash chromatography (hexanes-EtOAc 9:1) to afford *title compound* **11** (2.26 g, 39%) as a colorless oil. **Rf**  0.69 (hexanes-EtOAc 4:1); $\boldsymbol{\alpha}_{\boldsymbol{D}}^{\mathbf{21}}=$ + 40.8 (*c* 1.00, CHCl3); **IR** νmax (neat): 3364, 3035, 2980, 2936, 2112, 1740, 1714, 1499, 1456 cm-1; **1H NMR** (400 MHz, CDCl3): *δ* 7.40 – 7.34 (m, 5H), 5.33 (d, *J* = 7.5 Hz, 1H), 5.21 (ABq, Δδ = 0.05, *JAB* = 12.2 Hz, 2H), 4.47 – 4.45 (m, 1H), 3.85 – 3.82 (m, 1H), 1.45 (s, 9H), 1.29 (d, *J* = 6.9 Hz, 3H); **13C NMR** (100 MHz, CDCl3): *δ* 169.6, 155.2, 135.0, 128.81, 128.78, 128.6, 80.6, 67.7, 58.9, 57.6, 28.4, 15.6; **HRMS** *m/z* (ESI/Q-TOF) [M + Na]+ calcd for C16H22NaN4O4, 357.1533; found, 357.1522. The spectroscopic data were in agreement with those reported in the literature but no optical rotation was reported.7

**2-(1'-Hydroxyethylidene)-5,5-dimethylcyclohexane-1,3-dione (13)**

To a stirred solution of dimedone (4.0 g, 28.5 mmol) in CH2Cl2 (180 mL) was added DMAP (0.17 g, 1.43 mmol), DIPEA (5.2 mL, 30.0 mmol) and Ac2O (2.8 mL, 30.0 mmol). The reaction was stirred at room temperature for 24 h then diluted with CH2Cl2 (70 mL) and sequentially washed with 1 M aq. HCl (100 mL), sat. aq. NaHCO3 (100 mL) and brine (100 mL). The organic layer was dried over anhydrous Na2SO4, filtered and concentrated *in vacuo*. The crude residue was purified by flash chromatography (hexanes-EtOAc 7:3) to afford *title compound* **13** (2.40 g, 46%) as a pale yellow solid. **Rf**  0.74 (hexanes-EtOAc 7:3); **IR** νmax (neat): 2958, 2872, 1660, 1545, 1441, 1420, 1407, 1389 cm-1; **1H NMR** (400 MHz, CDCl3): *δ* 2.61 (s, 3H), 2.53 (s, 2H), 2.36 (s, 2H), 1.08 (s, 6H); **13C NMR** (100 MHz, CDCl3): *δ* 202.5, 198.0, 195.3, 112.5, 52.6, 47.0, 30.7, 28.6, 26.3; **HRMS** *m/z* (ESI/Q-TOF) [M + Na]+ calcd for C10H14NaO3, 205.0835; found, 205.0828. The spectroscopic data were in agreement with those reported in the literature.8

**(2*S*,3*S*)-2-((((9*H*-Fluoren-9-yl)methoxy)carbonyl)amino)-3-((1'-(4'',4''-dimethyl-2'',6''-dioxocyclohexylidene) ethyl)amino)butanoic acid** **(8)**

A mixture of azide **11** (0.50 g, 1.50 mmol) and 10% *w/w* Pd/C (0.10 g, 0.90 mmol) in degassed MeOH (80 mL) was stirred under an atmosphere of H2 (balloon) at room temperature for 2 h. The mixture was filtered through Celite® and concentrated *in vacuo* to afford crude amine **48**, which was submitted directly to the next step without further purification.

To a stirred solution of crude amine **48** (0.33 g, 1.50 mmol) in EtOH(7 mL) was added Dde-OH (**13**) (0.37 g, 1.94 mmol) and NEt3 (0.31 mL, 2.24 mmol). The reaction was heated to reflux for 18 h. The solution was allowed to cool to room temperature then concentrated *in vacuo* to afford crude acid **49**, which was submitted directly to the next step without further purification.

To a stirred solution of Boc-amino acid **49** (0.57 g, 1.49 mmol) in CH2Cl2 (5.2 mL) was added TFA (1.8 mL) and the reaction was stirred at room temperature for 3 h. The solution was concentrated under a stream of nitrogen to give crude amine **50**, which was submitted directly to the next step without further purification.

To a stirred solution of crude amine **50** (0.42 g, 1.49 mmol) in H2O (12 mL) at 0 °C was added NaHCO3 until pH 8, followed by the dropwise addition of Fmoc-OSu (0.50 g, 1.49 mmol) in acetone (4 mL) over 10 min. The reaction mixture was allowed to warm to room temperature, stirred for 2 d then concentrated *in vacuo* to remove the acetone. The mixture was acidified to pH 5 with 1 M aq. HCl then extracted with EtOAc (3 × 30 mL). The aqueous layer was further acidified to pH 2 then extracted with EtOAc (3 × 30 mL). The combined organic extracts were dried over anhydrous Na2SO4, filtered and concentrated *in vacuo*. The resulting crude **8** (0.23 g, 31% over four steps) was used without further purification. **HRMS** *m/z* (ESI/Q-TOF) [M + Na]+ calcd for C29H33NaN2O6, 505.2333; found, 505.2317.

## Synthesis of (2*S*,3*S*)-Fmoc-3-Hy(TBS)Asp(O*t*Bu)-OH (14)

***tert*-Butyl (*E*)-4-(benzyloxy)but-2-enoate (15)**

To a stirred solution of 2-(benzyloxy)acetaldehyde (2.00 g, 13.3 mmol) in CH3CN (57 mL) was added *tert*-butyl diethylphosphonate (3.3 mL, 14.0 mmol), LiCl (3.78 g, 89.2 mmol), and DIPEA (1.7 mL, 9.85 mmol). The reaction was stirred at room temperature for 2 d then quenched with H2O (30 mL) and extracted with EtOAc (3 × 50 mL). The combined organic extracts were dried over anhydrous Na2SO4, filtered and concentrated *in vacuo*. The crude residue was purified by flash chromatography (hexanes-EtOAc 9:1) to afford *title compound* **15** (2.26 g, 68%) as a colorless oil. **Rf**  0.30 (hexanes-EtOAc 9:1); **IR** νmax (neat): 2979, 1712, 1305, 1150, 1118, 736, 697 cm-1; **1H NMR** (400 MHz, CDCl3): *δ* 7.37 – 7.27 (m, 5H), 6.88 (dt, *J* = 4.5, 15.6, Hz, 1H), 6.04 (dt, *J* = 1.9, 15.7 Hz, 1H), 4.56 (s, 2H), 4.16 (dd, *J* = 2.0, 4.5 Hz, 2H), 1.48 (s, 9H); **13C NMR** (100 MHz, CDCl3): *δ* 165.6, 142.9, 137.8, 128.5, 127.8, 127.7, 123.4, 80.4, 72.8, 68.7, 28.1; **HRMS** *m/z* (ESI/Q-TOF) [M + Na]+ calcd for C15H20NaO3, 271.1305; found, 271.1309. The spectroscopic data were in agreement with those reported in the literature.9

***tert*-Butyl (2*S*,3*R*)-3-((((9*H*-fluoren-9-yl)methoxy)carbonyl)amino)-4-(benzyloxy)-2-hydroxy butanoate(16)**

To a stirred slurry of FmocNHCl (**19**) (2.22 g, 8.11 mmol) in *n*-propanol (18.5 mL) at 0 °C was added a solution of NaOH (244 mg, 6.09 mmol) in H2O (30 mL), (DHQD)2PHAL (380 mg, 0.49 mmol) in *n*-propanol (30 mL) and ester **15** (1.00 g, 4.06 mmol) in *n*-propanol (15 mL). The reaction was allowed to cool to 0 °C then K2OsO4·2H2O (120 mg, 0.32 mmol) in H2O (13 mL) and NaOH (30.9 mg, 0.77 mmol) were added. The reaction was stirred for 4.5 h then quenched with 1 M aq. HCl (30 mL) and H2O (100 mL) then extracted with EtOAc (2 × 100 mL). The combined organic extracts were washed with sat. aq. NaHCO3 (100 mL), brine (100 mL), dried over anhydrous Na2SO4, filtered and concentrated *in vacuo*. The crude residue was purified by flash chromatography (hexanes-EtOAc 4:1) to afford *title compound* **16** (1.68 g, 83%) as a colorless oil. **Rf**  0.32 (hexanes-EtOAc 4:1); $\boldsymbol{\alpha}_{\boldsymbol{D}}^{\mathbf{21}}=$ + 2.1(*c* 1.00, CHCl3); **IR** νmax (neat): 3432, 2980, 1722, 1511, 1247, 1123, 728 cm-1; **1H NMR** (400 MHz, CDCl3): *δ* 7.78 (d, *J* = 7.5 Hz, 2H), 7.59 (t, *J* = 7.5 Hz, 2H), 7.41 (t, *J* = 7.5 Hz, 2H), 7.36 – 7.28 (m, 7H), 5.29 (d, *J* = 10.0 Hz, 1H), 4.59 (ABq, Δδ = 0.03, *JAB* = 12.0 Hz, 2H), 4.45 – 4.28 (m, 4H), 4.22 (t, *J* = 7.0 Hz, 1H), 3.66 – 3.54 (m, 2H), 3.41 (d, *J* = 3.0 Hz, 1H), 1.48 (s, 9H); **13C NMR** (100 MHz, CDCl3): *δ* 172.6, 155.8, 143.8, 141.3, 137.9, 128.5, 128.0, 127.8, 127.7, 127.1, 125.1, 120.0, 83.6, 73.4, 69.6, 69.0, 67.0, 52.3, 47.2, 27.9; **HRMS** *m/z* (ESI/Q-TOF) [M + Na]+ calcd for C30H33NaNO6, 526.2200; found, 526.2193. The spectroscopic data were in agreement with those reported in the literature but no optical rotation was reported.9

***tert*-Butyl (2*S*,3*R*)-3-((((9*H*-fluoren-9-yl)methoxy)carbonyl)amino)-4-(benzyloxy)-2-((*tert*-butyl dimethylsilyl)oxy)butanoate (17)**

To a stirred solution of amino alcohol **16** (1.50 g, 2.98 mmol) and 2,6-lutidine (0.69 mL, 5.96 mmol) in dry CH2Cl2 (9 mL) was added TBSOTf (1.0 mL, 4.47 mmol) and the reaction was stirred for 2 h. The reaction was quenched with H2O (10 mL) and extracted with CH2Cl2 (3 × 20 mL). The combined organic extracts were dried over anhydrous Na2SO4, filtered and concentrated *in vacuo*. The crude residue was purified by flash chromatography (hexanes-EtOAc 9:1) to afford *title compound* **17** (1.46 g, 80%) as a colorless oil. **Rf**  0.28 (hexanes-EtOAc 9:1); $\boldsymbol{\alpha}_{\boldsymbol{D}}^{\mathbf{21}}=$ ‒ 4.8(*c* 1.01, CHCl3); **IR** νmax (neat): 3443, 2930, 2858, 1728, 1504, 1128, 836 cm-1; **1H NMR** (400 MHz, CDCl3):*δ* 7.78 (d, *J* = 7.6 Hz, 2H), 7.64 – 7.60 (m, 2H), 7.44 – 7.30 (m, 9H), 5.28 (d, *J* = 9.3 Hz, 1H), 4.59 (ABq, Δδ = 0.03, *JAB* = 11.8 Hz, 2H), 4.53 – 4.26 (m, 6H), 3.62 (dd, *J* = 5.5, 9.1 Hz, 1H), 3.55 (dd, *J* = 9.0, 9.0 Hz, 1H), 1.47 (s, 9H), 1.00 (s, 9H), 0.19 (s, 3H), 0.11 (s, 3H); **13C NMR** (100 MHz, CDCl3): *δ* 171.0, 155.9, 144.1*, 143.9, 141.3, 137.9, 128.4, 127.8, 127.72, 127.67, 127.1, 125.3*, 125.2, 120.1*, 120.0, 81.8, 73.2, 70.5, 68.4, 67.1, 53.4, 47.3, 28.0, 25.9, 18.4, -4.6, -5.5; *denotes signals arising from minor rotamers. **HRMS** *m/z* (ESI/Q-TOF) [M + Na]+ calcd for C36H47NaNO6Si, 640.3065; found, 640.3045.

***tert*-Butyl (2*S*,3*R*)-3-((((9*H*-fluoren-9-yl)methoxy)carbonyl)amino)-2-((*tert*-butyldimethylsilyl) oxy)-4-hydroxybutanoate (18)**

A mixture of benzyl alcohol **17** (500 mg, 0.81 mmol) and 10% *w/w* Pd/C (50 mg, 0.81 mmol) in degassed MeOH (40 mL) was stirred under an atmosphere of H2 (balloon) at room temperature for
24 h. Additional 10% *w/w* Pd/C (50 mg, 0.81 mmol) was added and the reaction was stirred under an atmosphere of H2 (balloon) at room temperature for a further 20 h. The mixture was filtered through Celite® and concentrated *in vacuo* to afford crude amino alcohol, which was submitted directly to the next step without further purification.

To a solution of the crude residue (250 mg) in H2O (6.5 mL) at 0 °C was added NaHCO3 until pH 8, followed by the dropwise addition of Fmoc-OSu (273 mg, 0.81 mmol) in acetone (2.2 mL) over 10 min. The reaction mixture was allowed to warm to room temperature, stirred for 2 d then concentrated *in vacuo* to remove the acetone. The mixture was acidified to pH 5 with 1 M aq. HCl then extracted with EtOAc (3 × 10 mL). The aqueous layer was further acidified to pH 2 then extracted with EtOAc (3 × 10mL). The combined organic extracts were dried over anhydrous Na2SO4, filtered and concentrated *in vacuo*. The crude residue was purified by flash chromatography (hexanes-EtOAc
9:1 → 4:1) to afford *title compound* **18** (250 mg, 65% over two steps) as a white solid. **Rf**  0.42 (hexanes-EtOAc 4:1); $\boldsymbol{\alpha}_{\boldsymbol{D}}^{\mathbf{20}}=$ ‒ 6.9(*c* 0.90, CHCl3); **IR** νmax (neat): 3451, 2928, 2859, 1731, 1471, 1130, 1124, 780 cm-1; **1H NMR** (400 MHz, CDCl3): *δ* 7.75 (d, *J* = 7.6 Hz, 2H), 7.60 (d, *J* = 7.5 Hz, 2H), 7.39 (d, *J* = 7.5 Hz, 2H), 7.30 (d, *J* = 7.5 Hz, 2H), 5.44 (d, *J* = 9.3 Hz, 1H), 4.48 – 4.14 (m, 6H), 3.80 (dd, *J* = 5.5, 9.1 Hz, 1H), 3.64 (dd, *J* = 9.0, 9.0 Hz, 1H), 1.46 (s, 9H), 0.99 (s, 9H), 0.19 (s, 3H), 0.12 (s, 3H); **13C NMR** (100 MHz, CDCl3): *δ* 171.2, 156.5, 144.0, 143.8*, 141.3, 127.7, 127.1, 125.3*, 125.2, 120.0, 82.0, 70.9, 67.2, 61.8, 55.7, 47.2, 27.9, 25.8, 18.4, -4.7, -5.5; *denotes signals arising from minor rotamers; **HRMS** *m/z* (ESI/Q-TOF) [M + Na]+ calcd for C29H41NaNO6Si, 550.2595; found, 550.2605.

**(2*S*,3*S*)-2-((((9*H*-Fluoren-9-yl)methoxy)carbonyl)amino)-4-(*tert*-butoxy)-3-((*tert*-butyldimethyl silyl)oxy)-4-oxobutanoic acid (14)**

To a solution of alcohol **18** (150 mg, 0.28 mmol) in CH3CN (4.0 mL) at 0 °C was added TEMPO (66.6 mg, 0.423 mmol). NaClO2 (72.0 mg, 0.80 mmol) in ice cold phosphate buffer (3.5 mL, pH ca. 7.0) was added dropwise, followed by addition of a 6% aq. of NaOCl (0.26 mL) over 30 min. The reaction was stirred for 3 h at 0 °C then quenched with sat. aq. Na3SO3 and allowed to warm to room temperature. The solution was acidified to pH 2 with 1 M aq. HCl then extracted with CH2Cl2 (3 × 10 mL). The combined organic extracts were dried over anhydrous Na2SO4, filtered and concentrated *in vacuo*. The crude residue was purified by flash chromatography (hexanes-EtOAc 3:2 with 1% AcOH) to afford *title compound* **14** (100 mg, 65%) as a white solid. **Rf**  0.21 (hexanes-EtOAc 4:1); $\boldsymbol{\alpha}_{\boldsymbol{D}}^{\mathbf{18}}=$ ‒ 5.3(*c* 1.00, CHCl3); **IR** νmax (neat): 3443, 2953, 2930, 2858, 1722, 1132, 838, 738 cm-1; **1H NMR** (400 MHz, CDCl3): *δ* 8.00 (s, 1H), 7.76 (d, *J* = 7.5 Hz, 2H), 7.61 (d, *J* = 7.4 Hz, 2H), 7.40 (t, *J* = 7.5 Hz, 2H), 7.30 (d, *J* = 7.5 Hz, 2H), 5.59 (d, *J* = 9.5 Hz, 1H), 4.90 – 4.86 (m, 1H), 4.74 (d, *J* = 1.9 Hz, 1H), 4.39 – 4.33 (m, 2H), 4.25 (t, *J* = 7.3 Hz, 1H), 1.45 (s, 9H), 0.92 (s, 9H), 0.16 (s, 3H), 0.07 (s, 3H); **13C NMR** (100 MHz, CDCl3): *δ* 174.5, 168.9, 156.2, 143.83, 143.76*, 141.3, 127.7, 127.1, 124.2, 120.0, 82.7, 72.4, 67.6, 57.5, 47.1, 27.9, 25.6, 18.2, -4.7, -5.7; *denotes signals arising from minor rotamers; **HRMS** *m/z* (ESI/Q-TOF) [M ‒ H]+ calcd for C29H38NO7Si, 540.2423; found, 540.2419.

**(9*H*-Fluoren-9-yl)methyl chlorocarbamate (19)**

A solution of 9-fluorenylmethyl carbamate (5.00 g, 20.9 mmol) in MeOH (313 mL) was heated until the solution became clear, then allowed to cool to room temperature. Trichloroisocyanuric acid
(1.62 g, 6.97 mmol) was added and the reaction was stirred at room temperature for 18 h then concentrated *in vacuo*. The crude residue was purified by flash chromatography (hexanes-EtOAc 17:3) to afford *title compound* **19** (4.15 g, 73%) as a white solid. **Rf**  0.35 (hexanes-EtOAc 4:1); **mp** 142.2 – 144.5 °C; **IR** νmax (neat): 3199, 1700, 1468, 1451, 1444, 1247, 1109, 734 cm-1; **1H NMR** (400 MHz, CDCl3): *δ* 7.78 (d, *J* = 7.6 Hz, 2H), 7.61 (dd, *J* = 0.9, 7.5 2H), 7.42 (td, *J* = 0.9, 7.6 Hz, 2H), 7.33 (td, *J* = 1.0, 7.4 Hz, 2H), 5.67 (s, 1H), 4.50 (d, *J* = 7.3 Hz, 2H), 4.27 (t, *J* = 7.1 Hz, 1H); **13C NMR** (100 MHz, CDCl3): *δ* 156.4, 143.2, 141.4, 128.0, 127.2, 125.1, 120.1, 69.3, 46.9; **HRMS** *m/z* (ESI/Q-TOF) [M + Na]+ calcd for C15H12ClNaO2, 296.0449; found, 296.0449. The spectroscopic data were in agreement with those reported in the literature.10

## Synthesis of (2*S*,4*R*)-Fmoc-3-MePro-OH (20)

**Di-*tert*-butyl (2*S*,4*R*)-4-hydroxypyrrolidine-1,2-dicarboxylate (21)**

To a stirred solution of *trans*-4-hydroxy-L-proline (10.0 g, 51.1 mmol) in THF (70 mL) and H2O
(35 mL) was added 10% aq. NaOH (21.5 mL) followed by Boc2O (16.7 g, 76.7 mmol). The biphasic solution was vigorously stirred at room temperature for 26 h then concentrated *in vacuo*. The resulting mixture was acidified to pH 2 using 10% aq. KHSO4, extracted with EtOAc (3 × 80 mL), then washed with H2O (250 mL) and brine (250 mL). The combined organic extracts were dried over anhydrous Na­2SO4, filtered and concentrated *in vacuo* to afford the crude acid, which was submitted directly to the next step without further purification.

To a stirred solution of the crude acid(11.7 g, 51.1 mmol) in CH2Cl2 (120 mL) was added *tert*-butyl (*Z*)-*N,N'*-diisopropylcarbamimidate (0.8 M in CH2Cl2, 53.5 mL, 42.8 mmol) and the mixture was stirred at room temperature for 17 h. Another portion of *tert*-butyl (*Z*)-*N,N'*-diisopropylcarbamimidate (0.8 M in CH2Cl2, 12 mL, 9.60 mmol) was added and the mixture was stirred for an additional 24 h. The mixture was filtered through Celite® and concentrated *in vacuo*. The crude residue was purified by flash chromatography (hexanes-EtOAc 4:1) to afford *title compound* **21** (8.27 g, 74% over two steps) as a colorless oil. **Rf**  0.14 (hexanes-EtOAc 3:2); $\boldsymbol{\alpha}_{\boldsymbol{D}}^{\mathbf{22}}=$ ‒ 57.0 (*c* 1.03, CHCl3) (lit.11 $\boldsymbol{\alpha}_{\boldsymbol{D}}^{\mathbf{23}}=$ ‒ 57.3 (*c* 1.01, CHCl3)); **IR** νmax (neat): 3458, 2975, 2932, 1718, 1685 cm-1; **1H NMR** (400 MHz, CDCl3): *δ* 4.50 – 4.40 (m, 1H), 4.31 – 4.24 (m, 1H), 3.62 – 3.39 (m, 2H), 2.31 – 2.20 (m, 2H), 2.09 – 1.99 (m, 1H), 1.45 – 1.42 (m, 18H); **13C NMR** (100 MHz, CDCl3): *δ* 172.3, 172.2*, 154.6, 154.4*, 81.3, 81.2*, 80.3, 80.0*, 70.3*, 69.4, 58.7, 58.6*, 54.7*, 54.7, 39.3, 38.6*, 28.5, 28.1, 28.0*; *denotes signals arising from minor rotamers; **HRMS** *m/z* (ESI/Q-TOF) [M + Na]+ calcd for C14H25NNaO5, 310.1625; found, 310.1631. The spectroscopic data were in agreement with those reported in the literature.11

**Di-*tert*-butyl (*S*)-4-oxopyrrolidine-1,2-dicarboxylate (22)**

To a stirred solution of protected amino acid **21** (5.00 g, 17.4 mmol) in CH2Cl2 (70 mL) was added Dess-Martin periodinane (11.1 g, 26.1 mmol) at 0 °C. The mixture was allowed to warm to room temperature then stirred for 4 h. The reaction was quenched with sat. aq. NaHCO3 (100 mL) and H2O (100 mL), extracted with CH2Cl2 (3 × 80 mL), then washed with sat. aq. Na2S2O3 (120 mL) and brine (120 mL). The combined organic extracts were dried over anhydrous Na2SO4, filtered and concentrated *in vacuo*. The crude residue was purified by flash chromatography (hexanes-EtOAc 17:3) to afford *title compound* **22** (4.63 g, 92%) as a white solid. **Rf** 0.39 (hexanes-EtOAc 9:1); $\boldsymbol{\alpha}_{\boldsymbol{D}}^{\mathbf{22}}=$+ 9.6 (*c* 1.00, CHCl3) (lit.12 $\boldsymbol{\alpha}_{\boldsymbol{D}}^{\mathbf{22}}=$+ 6.5 (*c* 0.77, CHCl3)); **IR** νmax (neat): 2980, 2939, 2896, 1762, 1732, 1704, 1688 cm-1; **1H NMR** (400 MHz, CDCl3): *δ* 4.66 – 4.65 (m, 1H), 3.88 – 3.83 (m, 2H), 2.95 – 2.86 (m, 1H), 2.53 – 2.47 (m, 1H), 1.46 – 1.44 (m, 18H); **13C NMR** (100 MHz, CDCl3): *δ* 209.0*, 208.2, 170.9, 154.4, 153.7*, 82.4, 81.1*, 57.1, 56.7*, 53.0*, 52.6, 41.5, 41.0*, 28.3, 28.0; *denotes signals arising from minor rotamers; **HRMS** *m/z* (ESI/Q-TOF) [M + Na]+ calcd for C14H23NNaO5, 308.1468; found, 308.1461. The spectroscopic data were in agreement with those reported in the literature.12

**Di-*tert*-butyl (*S*)-4-methylenepyrrolidine-1,2-dicarboxylate (23)**

To a stirred mixture of recrystallized methyltriphenylphosphonium bromide (8.26 g, 23.1 mmol) in THF (80 mL) was added freshly sublimed *t*BuOK (2.51 g, 22.4 mmol) at 0 °C. The mixture was allowed to warm to room temperature and stirred for 2 h. The reaction was cooled to 0 °C and ketone **22** (2.2 g, 7.71 mmol) in THF (9 mL) was added dropwise over 2 min. The mixture was allowed to warm to room temperature, stirred for 1 h then quenched with sat. aq. NH4Cl (60 mL). The reaction mixture was extracted with EtOAc (3 × 50 mL) and washed with brine (100 mL). The combined organic extracts were dried over anhydrous Na2SO4, filtered and concentrated *in vacuo*. The crude residue was purified by flash chromatography (hexanes-EtOAc 9:1) to give *title compound* **23** (1.93 g, 88%) as a pale yellow oil. **Rf**  0.29 (hexanes-EtOAc 19:1); $\boldsymbol{\alpha}_{\boldsymbol{D}}^{\mathbf{22}}=$– 22.4 (*c* 1.00, CHCl3) (lit.13 $\boldsymbol{\alpha}_{\boldsymbol{D}}^{\mathbf{25}}=$– 20.9 (*c* 1.01, CHCl3)) ; **IR** νmax (neat): 2977, 2933, 2870, 1741, 1702 cm-1; **1H NMR** (400 MHz, CDCl3): *δ* 4.98 – 4.96 (m, 2H),
4.36 – 4.24 (m, 1H), 4.05 – 4.00 (m, 2H), 2.98 – 2.85 (m, 1H), 2.56 – 5.52 (m, 1H), 1.45 – 1.42 (m, 18H); **13C NMR** (100 MHz, CDCl3): *δ* 171.9, 171.8*, 154.5*, 154.0, 144.1*, 142.9, 107.7, 107.5*, 81.3, 80.1, 79.9*, 59.8, 59.6*, 51.0*, 50.7, 37.1, 36.6*, 28.5, 28.1; *denotes signals arising from minor rotamers; **HRMS** *m/z* (ESI/Q-TOF) [M + Na]+ calcd for C15H25NNaO4, 306.1676; found, 306.1681. The spectroscopic data were in agreement with those reported in the literature.13

**Di-*tert*-butyl (2*S*,4*R*)-4-methylpyrrolidine-1,2-dicarboxylate (24)**

A mixture of amino acid **23** (100 mg, 0.35 mmol) and Crabtree’s catalyst (10.0 mg, 0.014 mmol) in degassed 1,2-dichloroethane (5 mL) was stirred under an atmosphere of H2 (balloon) at room temperature for 5 d. The mixture was concentrated *in vacuo* and the crude residue was purified by flash chromatography (hexanes-EtOAc 9:1) to afford *title compound* **24** (76 mg, 78%) as a colorless oil. **Rf** 0.36 (hexanes-EtOAc 9:1); $\boldsymbol{\alpha}_{\boldsymbol{D}}^{\mathbf{23}}=$– 38.8 (*c* 1.00, CHCl3); **IR** νmax (neat): 2976, 2932, 2876, 1742, 1699, 1479, 1457, 1393, 1365 cm-1; **1H NMR** (400 MHz, CDCl3): *δ* 4.22 – 4.10 (m, 1H), 3.70 – 3.59 (m, 1H), 2.95 – 2.84 (m, 1H), 2.42 – 2.29 (m, 1H), 2.03 – 1.98 (m, 1H), 1.83 – 1.69 (m, 1H), 1.44 – 1.41 (m, 18H), 1.02 – 1.00 (m, 3H); **13C NMR** (100 MHz, CDCl3): *δ* 172.4, 172.3*, 154.4*, 154.0, 80.9, 79.6, 79.5*, 60.1, 60.0*, 53.6*, 53.2, 38.7, 37.8*, 32.0* , 31.1, 28.5, 28.1, 17.6; *denotes signals arising from minor rotamers; **HRMS** *m/z* (ESI/Q-TOF) [M + Na]+ calcd for C15H27NNaO4, 308.1832; found, 308.1829.

**(2*S*,4*R*)-1-(((9*H*-Fluoren-9-yl)methoxy)carbonyl)-4-methylpyrrolidine-2-carboxylic acid (20)**

To a stirred solution of ester **24** (0.70 g, 2.45 mmol) in TFA (17.5 mL) was added triisopropylsilane (0.9 mL) and the reaction was stirred for 12 h. The solution was concentrated under a stream of N2 gas to give the crude amine, which was submitted directly to the next step without further purification.

To a stirred solution of the crude amine (0.56 g, 2.45 mmol) in sat. aq. NaHCO3 (14 mL) was added Fmoc-OSu (0.90 g, 2.68 mmol) in dioxane (14 mL). The reaction was stirred for 2 d then diluted withH2O (15 mL) and extracted with EtOAc (3 × 20 mL). The aqueous layer was acidified to pH 3 with 1 M aq. HCl then further extracted with EtOAc (3 × 20 mL). The combined organic extracts were dried over anhydrous Na2SO4, filtered and concentrated *in vacuo*. The resulting crude was purified by flash chromatography (CH2Cl2-MeOH-AcOH 99:0.5:0.5) to afford *title compound* **20** (0.80 g, 89% over two steps) as a fluffy white solid. **Rf**  0.29 (hexanes-EtOAc 7:3 with trace AcOH); $\boldsymbol{\alpha}_{\boldsymbol{D}}^{\mathbf{20}}=$– 49.4 (*c* 1.00, CHCl3) (lit.14 $\boldsymbol{\alpha}_{\boldsymbol{D}}^{\mathbf{20}}=$– 49.3 (*c* 0.3, CHCl3)); **IR** νmax (neat): 2958, 2875, 1747, 1704, 1662, 1653, 1478, 1451, 1419, 1353, 1241, 1170, 1148 cm-1; **1H NMR** (400 MHz, CDCl3): *δ* 9.52 (bs, 1H), 7.78 – 7.70 (m, 2H), 7.62 – 7.53 (m, 2H), 7.42 – 7.27 (m, 4H), 4.49 – 4.37 (m, 3H), 4.28 – 4.13 (m, 1H), 3.78 – 3.72 (m, 1H), 3.04 – 2.99 (m, 1H), 2.51 – 2.35 (m, 1H), 2.31 – 2.14 (m, 1H), 1.93 – 1.78 (m, 1H), 1.09 – 1.03 (m, 3H); **13C NMR** (100 MHz, CDCl3): *δ* 178.1*, 176.3, 155.9, 154.4*, 144.1*, 143.9, 143.81*, 143.77, 141.34, 141.28*, 127.8, 127.6*, 127.12, 127.07*, 125.14, 125.07, 125.0*, 124.9*, 120.0, 119.9*, 67.9, 67.5*, 59.6, 58.9*, 53.7*, 53.4, 47.2⁕, 38.6*, 36.9, 32.2, 31.1*, 17.2⁕; *denotes signals arising from minor rotamers, ⁕denotes signals arising from all rotamers; **HRMS** *m/z* (ESI/Q-TOF) [M + Na]+ calcd for C21H21NNaO4, 374.1363; found, 374.1363. The spectroscopic data were in agreement with those reported in the literature.15

## Synthesis of (2*S*,4*R*)-Fmoc-3-MePro-OH (25)

**1-Bromo-5-methylhexyne (26)**

To a stirred solution of 5-methylhexyne (12.0 g, 125 mmol) in acetone (400 mL) was added NBS
(27.8 g, 156 mmol) and AgNO3 (2.12 g, 12.5 mmol) and the mixture was stirred at room temperature for 4 h. The reaction was poured over ice water (400 mL) then extracted with pentane (3 × 150 mL). The combined organic extracts were dried over anhydrous Na2SO4, filtered and concentrated *in vacuo* (30°C at 200 mbar minimum pressure) to afford *title compound* **26** (20.5 g, 94%) as a yellow oil. **Rf** 0.41 (hexanes-EtOAc 19:1); **IR** νmax (neat): 2960, 2931, 2872, 1715 cm-1; **1H NMR** (400 MHz, CDCl3,): *δ* 2.21 (t, *J* = 7.3 Hz, 2H), 1.73 – 1.61 (m, 1H), 1.45 – 1.37 (d, *J* = 7.2, 7.3 Hz, 2H), 0.89 (d, *J* = 6.5 Hz, 6H); **13C NMR** (100 MHz, CDCl3) *δ* 80.6, 37.4, 37.3, 27.3, 22.2, 17.8; It was not possible to obtain a HRMS for this compound.

**1-Bromo-5-methylhexene (27)**

To a stirred solution of cyclohexene (21 mL, 206 mmol) in Et2O (75 mL) at 10 °C was added borane dimethyl sulfide complex (9.4 mL, 98.5 mmol) and the mixture was stirred until the appearance of precipitate. The reaction was allowed to warm to room temperature and stirred for a further 20 min. The slurry was cooled to 15 °C and 1-bromo-5-methylhexyne (**26**) (15.0 g, 175 mmol) in pentane (105 mL) was added dropwise over 30 min. The reaction was allowed to warm to room temperature and stirred for 40 min. The solution was cooled to 20 °C and AcOH (5.4 mL, 94.3 mmol) was addedand stirred for 15 min. Ethanolamine (12.0 mL, 197 mmol) was then added and stirred for a further 30 min. The mixture was filtered through a pad of silica, eluting with pentane-Et2O (4:1), then concentrated *in vacuo* (30 °C at 250 mbar minimum pressure) to afford *title compound* **27** (15.0 g, 99%) as a yellow oil. **Rf** 0.41 (hexanes-EtOAc 49:1); **IR** νmax (neat): 2956, 2932, 2870, 2856, 1623, 1448, 672, 662 cm-1; **1H NMR** (400 MHz, CDCl3): *δ* 6.13 (dt, *J* = 1.5, 6.8 Hz, 1H), 6.07 (dt, *J* = 6.8, 6.8 Hz, 1H), 2.16 – 2.24 (m, 2H), 1.52 – 1.64 (m, 1H), 1.34 – 1.27 (m, 2H), 0.91 (d, *J* = 6.7 Hz, 6H); **13C NMR** (100 MHz, CDCl3): *δ* 135.3, 107.5, 37.3, 27.9, 27.8, 22.6; It was not possible to obtain a HRMS for this compound.

**(*Z*)-8-Methylnon-4-en-2-yn-1-ol (28)**

To stirred solution of bis(triphenylphosphine)palladium (II) chloride (0.14 g, 0.20 mmol), copper (I) iodide (0.23 g, 1.2 mmol), triphenylphosphine (0.10 g 0.40 mmol) and propylamine (13.7 mL, 166 mmol) in Et2O (250 mL) was added 1-bromo-5-methylhexene (**27)** (14.0 g, 79.1 mmol), followed by dropwise addition of propargyl alcohol (5.5 mL, 94.9 mmol) over 5 min. The reaction mixture was heated to reflux and stirred for 24 h. The mixture was allowed to cool to room temperature then concentrated *in vacuo*. The remaining slurry was diluted with Et2O (150 mL) and washed with sat. aq. NaHCO3 (3 × 100 mL) then H2O until pH 7. The combined organic extracts were dried over anhydrous Na2SO4, filtered and concentrated *in vacuo*. The crude residue was purified by flash chromatography (hexanes-Et2O 9:1) to afford *title compound* **28** (8.86 g, 74%) as a pale yellow oil. **Rf**  0.33 (hexanes-EtOAc 9:1); **IR** νmax (neat): 3325, 2956, 2910, 2868, 2204, 1617 cm-1; **1H NMR** (400 MHz, CDCl3): *δ* 5.92 (dt, *J* = 7.6, 10.8 Hz, 1H), 5.51 – 5.43 (m, 1H), 4.42 (dd, *J* = 2.0, 6.0 Hz, 2H), 2.31 (dt, *J* = 7.6, 7.6 Hz, 2H), 2.01 (bs, 1H), 1.67 – 1.48 (m, 1H), 1.29 (dt, *J* = 7.6, 7.6 Hz, 2H), 0.90 (d, *J* = 6.7 Hz, 6H); **13C NMR** (100 MHz, CDCl3): *δ* 145.1, 108.0, 91.4, 82.6, 51.7, 38.0, 28.4, 27.7, 22.6; **HRMS** *m/z* (ESI/Q-TOF) [M + Na]+ calcd for C10H16NaO, 175.1093; found, 175.1090.

**(2*E*,4*Z*)-8-Methylnona-2,4-dien-1-ol (29)**

To a stirred mixture of LiAlH4 (2.19 g, 57.8 mmol) in THF (185 mL) was added alkyne **28** (8.00 g, 52.6 mmol) dropwise over 10 min. The reaction was heated to reflux and stirred for 4 h, then cooled to ‒10 °C and quenched with sat. aq. Rochelle’s salt (175 mL) and diluted with Et2O (150 mL). The biphasic mixture allowed to warm to room temperature and stirred for 1.5 h. The solution was left to separate, and the organic layer was decanted. The aqueous layer was extracted with Et2O (3 × 80 mL) and washed with brine (250 mL). The combined organic extracts were dried over anhydrous Na2SO4, filtered and concentrated *in vacuo*. The crude residue was purified by flash chromatography (hexanes-Et2O 4:1) to afford *title compound* **29** (7.74 g, 95%) as a colorless oil. **Rf :** 0.63 (hexanes-EtOAc 4:1); **IR** νmax (neat): 3305, 2955, 2906, 2870, 1655 cm-1; **1H NMR** (400 MHz, CDCl3): *δ* 6.61 – 6.47 (m, 1H), 5.98 (dd, *J* = 10.8, 10.8 Hz, 1H), 5.80 (dt, *J* = 6.0, 15.2 Hz, 1H), 5.44 (dt, *J* = 7.7, 10.8 Hz, 1H), 4.20 (t, *J* = 6.0 Hz, 2H), 2.23 – 2.14 (m, 2H), 1.67 – 1.49 (m, 1H), 1.31 – 1.22 (m, 2H), 0.88 (d, *J* = 6.6 Hz, 6H); **13C NMR** (100 MHz, CDCl3): *δ* 133.4, 131.6, 127.4, 126.9, 63.6, 38.8, 27.7, 25.7, 22.6; **HRMS** *m/z* (ESI/Q-TOF) [M + Na]+ calcd for C10H18NaO, 177.1250; found, 177.1249.

**(2*E*,4*Z*)-8-Methylnona-2,4-dienal (30)**

To a stirred solution of alcohol **29** (1.50 g, 9.72 mmol) in CH2Cl2 (40 mL) was added manganese (IV) oxide (16.9 g, 194 mmol) and the reaction was stirred at room temperature for 18 h. The mixture was filtered through a pad of Celite® and concentrated *in vacuo*. The crude residue was purified by flash chromatography (hexanes-Et2O 19:1) to afford *title compound* **30** (1.47 g, 99%) as a pale yellow oil. **Rf**  0.57 (hexanes-EtOAc 19:1); **IR** νmax (neat): 3018, 2956, 2929, 2870, 2717, 1679, 1630 cm-1; **1H NMR** (400 MHz, CDCl3): *δ* 9.61 (d, *J* = 7.9 Hz, 1H), 7.45 (ddd, *J* = 1.1, 11.5, 15.2 Hz, 1H), 6.31 – 6.19 (m, 1H), 6.14 (dd, *J* = 7.9, 15.2 Hz, 1H), 6.00 (dt, *J* = 7.8, 10.8 Hz, 1H), 2.39 – 2.28 (m, 2H), 1.68 – 1.50 (m, 1H), 1.39 – 1.28 (m, 2H), 0.92 (d, *J* = 6.6 Hz, 6H); **13C NMR** (100 MHz, CDCl3): *δ* 194.2, 147.0, 144.4, 131.8, 126.6, 38.4, 27.8, 26.5, 22.5; **HRMS** *m/z* (ESI/Q-TOF) [M + Na]+ calcd for C10H16NaO, 175.1093; found, 175.1090.

**(2*E*,4*Z*)-8-Methylnona-2,4-dienoic acid (25)**

To a stirred solution of aldehyde **30** (1.0 g, 6.57 mmol) in EtOH (132 mL) was added AgNO3 (2.45 g, 14.5 mmol) dissolved in H2O (4.6 mL) followed by the dropwise addition of 10% aq. NaOH solution (58 mL) over 15 min. The mixture was stirred at room temperature for 2.5 h, then filtered through a pad of Celite® and concentrated *in vacuo*. The resulting residue was diluted with H2O (40 mL) and extracted with Et2O (3 × 40 mL). The aqueous layer was acidified to pH 6 with 1 M aq. HCl and further extracted with Et2O (3 × 40 mL). The combined organic extracts were dried with anhydrous Na2SO4, filtered and concentrated *in vacuo* to give *title compound* **25** (0.63 g, 57%) as a pale yellow oil. **Rf**  0.46 (hexanes-EtOAc 3:2); **IR** νmax (neat): 3010, 2956, 2871, 2668, 2575, 2024, 1685, 1627, 1605 cm-1; **1H NMR** (400 MHz, CDCl3): *δ* 8.71 (bs, 1H), 7.70 (dd, *J* = 11.9, 15.0 Hz, 1H), 6.14 (dd, *J* = 11.3, 11.3 Hz, 1H), 5.93 – 5.85 (m, 2H), 2.34 – 2.28 (m, 2H), 1.63 – 1.53 (m, 1H), 1.33 – 1.28 (m, 2H), 0.91 (d, *J* = 6.4 Hz, 6H); **13C NMR** (100 MHz, CDCl3): *δ* 172.8, 143.4, 141.9, 126.3, 120.4, 38.6, 27.8, 26.5, 22.6; **HRMS** *m/z* (ESI/Q-TOF) [M + Na]+ calcd for C10H16NaO2, 191.1043; found, 191.1037. The spectroscopic data were in agreement with those reported in the literature.2

**General Procedures for Peptide Synthesis**

***General procedure 1* - Loading of 2-CTC resin:** 2-CTC resin (0.1 mmol, 1 eq.) was swollen in CH2Cl2 (5 mL) for 30 min. The resin was drained and a solution of Fmoc-AA-OH (0.4 mmol, 4 eq.) and DIPEA (0.8 mmol, 8 eq.) in CH2Cl2 (5 mL) was added. The resin was agitated for 2 h, filtered and washed with CH2Cl2 (3 × 5 mL). A capping solution consisting of CH2Cl2:MeOH:DIPEA (17:2:1, *v/v/v*, 5 mL) was added to the resin and agitated for 1 h. The resin was filtered, washed with CH2Cl2 (3 × 5 mL) and dried in air.

***General procedure 2* - Loading calculation**16**:** Two samples of air-dried peptidyl-resin (*ca.* 1.3 mg each) were treated with 20% piperidine in DMF (*v/v*, 3 mL) for 30 min with frequent agitation. A cuvette containing 20% piperidine in DMF (*v/v*, 3 mL) was used as a reference. The absorbances were measured at 290 nm with absorbance value of the reference cuvette set to zero. The resin-containing cuvettes were placed into the spectrometer, in turn. The measured absorbance values were used in **Equation 3.1** to determine *Experimental loading* for each sample. The *Loading yield* was then calculated using **Equations 3.2** and **3.3**.

$\boldsymbol{Experimental loading}\left( \frac{\boldsymbol{mmol}}{\boldsymbol{g}} \right)\boldsymbol{=}\frac{\boldsymbol{measured Absorbance}}{\boldsymbol{mg of sample\times1.75}}$ (**Equation 3.1**)

$\boldsymbol{Theoretical loading}\left( \frac{\boldsymbol{mmol}}{\boldsymbol{g}} \right)\boldsymbol{=}\frac{\boldsymbol{B\times1000}}{\boldsymbol{1000+}\left[ \boldsymbol{B\times}\left( \boldsymbol{M-X} \right) \right]}$ (**Equation 3.2**)

Where *B* is resin loading, *M* is the molecular weight of the added residue and *X* is the molecular weight of the removed residue (here 35.5).

$\boldsymbol{Loading yield=}\frac{\boldsymbol{Average Experimetal loading}}{\boldsymbol{Theoretical loading}}\boldsymbol{\times100}$ (**Equation 3.3**)

***General procedure 3* – Fmoc deprotection:** Peptidyl-resin was treated with 20% piperidine in DMF (*v/v*, 5 mL) for 5 min. The resin was filtered, and the procedure repeated once. The resin was filtered and washed with DMF (3 × 5 mL).

***General method 4* - Coupling of standard amino acids (Fmoc-Pro-OH, Fmoc-Val-OH, Fmoc-Asp(O*t*Bu)-OH, Fmoc-Lys(Boc)-OH):** To a solution of Fmoc-AA-OH (4 eq.) and HATU (3.8 eq.) dissolved in DMF (5 mL), DIPEA (8 eq.) was added and the resulting mixture was agitated for 30 s. The resulting activated coupling cocktail was added to peptidyl-resin and agitated for 30 min. The resin was filtered and washed with DMF (3 × 5 mL).

***General procedure 5* - Coupling of non-standard residues** **(Fmoc-D-Asp(O*t*Bu)-OH, Fmoc-(Dmb)Gly-OH, Fmoc-Dap(Alloc)-OH, Fmoc-D-Val-OH, decanoic acid, hexanoic acid, myristic acid, palmitic acid, 4-phenylbenzoic acid and biphenyl-4-carboxylic acid):** To a solution of Fmoc-AA-OH (2 eq.) and HCTU (1.8 eq.) in DMF (3 mL), DIPEA (4 eq.) was added and the resulting mixture was agitated for 30 s. The resulting activated coupling cocktail was added to peptidyl-resin and agitated for 1h. The resin was filtered and washed with DMF (3 × 5 mL).

***General procedure 6* - Coupling of synthesized building blocks ((2*R*,3*R*)-3-MeAsp(O*t*Bu)-OH (7), (2*S*,3*S*)-3-Hy(TBS)Asp(O*t*Bu)-OH (14), (2*S*,3*S*)-3-MeDap(Dde)-OH (8), (2*S*,3*S*)-3-MeAsp(O*t*Bu)-OH (2) and (2*E*,4*Z*)-8-methylnona-2,4-dienoic acid (25)):** To a solution of Fmoc-AA-OH (1.2 eq.), COMU (1.1 eq.) and Oxyma Pure (1.1 eq.) dissolved in DMF (2 mL), DIPEA (2.4 eq.) was added and the resulting mixture was agitated for 30 s. The resulting activated coupling cocktail was added to peptidyl-resin and agitated for 2 h. The resin was filtered and washed with DMF (3 × 5 mL).

***General procedure 7* - Alloc removal:** Peptidyl-resin was swollen in CH2Cl2 for 30 min, filtered and treated with a solution of Pd(PPh3)4 (1 eq.) and PhSiH3 (15 eq.) in CH2Cl2 (5 mL). The glass reaction vessel was wrapped in tin foil and agitated for 2 h. The resin was filtered and washed successively with CH2Cl2 (3 × 5 mL), 0.5 M solution of sodium diethyldithiocarbamate trihydrate in DMF (3 × 5 mL) and DMF (3 × 5 mL).

***General procedure 8* - TBS removal:** Peptidyl-resin was swollen in dry THF for 20 min, filtered and treated with a solution of TBAF (1 M in THF, 15 eq.) and AcOH (15 eq.) in dry THF (5 mL) and agitated for 6 h. The resin was filtered and washed with DMF (3 × 5 mL).

***General procedure 9* - Dde removal:** Peptidyl-resin was swollen in CH2Cl2 for 30 min, filtered and treated with a solution of 3.6 M NH2OH·HCl and 2.7 M imidazole in NMP:CH2Cl2 (5:1, *v/v*, 3 mL) and agitated for 4 h. The resin was filtered, washed with DMF (3 × 5 mL), CH2Cl2 (3 × 5 mL) and dried in air.

***General procedure 10* - Cleavage of side-chain protected peptide from the resin:** Peptidyl-resin was treated with a solution of 30% HFIP in CH2Cl2 (*v/v*, 5 mL) and agitated for 1 h. The filtrate was collected, and resin was washed with CH2Cl2 (3 × 1 mL). The combined filtrates were concentrated under a stream of N2 gas, re-dissolved in H2O:CH3CN (1:4, *v/v*, 20 mL) and lyophilized.

***General procedure 11* - Macrocyclization:** Lyophilized crude linear peptide was dissolved in DMF to make a 10 mM solution. To this solution DMTMM·BF4 (1.5 eq.) and DIPEA (2 eq.) were added and the mixture was agitated until starting material was consumed. The reaction mixture was diluted with H2O:CH3CN (1:1, *v/v*, such that for every 2 mL of the reaction mixture 38 mL of H2O:CH3CN were added) and lyophilized.

***General procedure 12* - Side chain deprotection of simplified analogues (31, 36-40):** Crude peptide was treated with a mixture of TFA:TIPS:H2O (95:2.5:2.5, *v/v/v*, 5 mL) and agitated for 2 h. TFA was removed under a stream of N2 gas, precipitated with cold Et2O (40 mL) and centrifuged. The Et2O layer was decanted and the procedure repeated once more before dissolving the obtained precipitate in H2O:CH3CN (0.1% TFA, 1:1, *v/v*, 20 mL) for lyophilization. The obtained crude peptide was purified by semi-preparative RP-HPLC.

***General procedure 13* - Side-chain deprotection of diastereomer (1a):** Crude peptide was treated with a mixture of TFA:CH2Cl2:TIPS:H2O (50:45:2.5:2.5, *v/v/v/v*, 3 mL) and agitated for 30 min. TFA was neutralized by addition of equal volume of sat. aq. NaHCO3 solution. CH2Cl2 were removed under a stream of N2 gas, and the resulting solution was extracted with 80% CH3CN in H2O (*v/v,* 5 × 3 mL) using a Waters Sep-Pak® C18 SPE cartridge and lyophilized. The obtained crude peptide was purified by semi-preparative RP-HPLC.

# Experimental for synthesis of analogues of malacidin A

## Synthesis of simplified analogue 31

Fmoc-Pro-OH was loaded onto 2-CTC resin (0.1 mmol, 0.81 mmol g-1) according to ***General Procedure 1***. The loading yield was calculated as 60% (0.49 mmol g-1) using ***General Procedure 2***. The linear peptide was assembled using iterative Fmoc deprotections as described in ***General Procedure 3***, standard amino acids were coupled according to ***General Procedure 4***, and non-standard amino acids and decanoic acids were coupled according to ***General Procedure 5***. A portion of the resin (0.048 mmol) was subjected to Alloc removal according to ***General Procedure 7*** and the obtained side chain protected linear peptide was cleaved from the resin as described in ***General Procedure 10***. The synthesis was proceeded at the 0.048 mmol scale. Crude peptide was subjected to overnight macrocyclization according to ***General Procedure 11*** followed by side chain deprotection using ***General Procedure 12***. Purification of crude cyclized peptide by RP-HPLC (Phenomenex Gemini C18 column (110 Å, 5 μm, 250 × 10 mm ) with gradient of 5-65% B at 1%B min-1 until 30% B then 0.5% B, and flow of 4 mL min-1) afforded purified simplified **31** (4.9 mg, 4.1 μmol, 9% yield based on 0.048 mmol scale, *ca*. 99% purity).


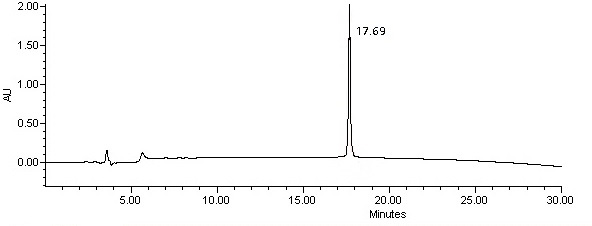

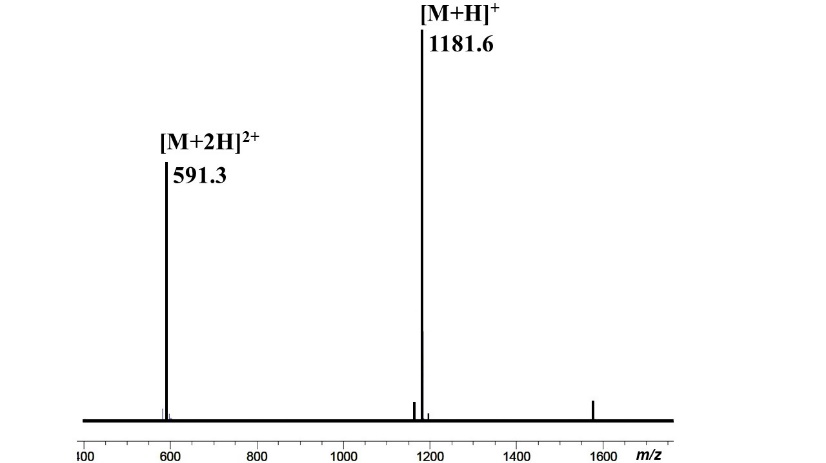


**Figure S1.** Analytical HPLC trace (214 nm) with ESI-MS spectrum of simplified analogue **31** (RT = 17.69 min, *ca.* 99% purity as judged by peak area of RP-HPLC at 214 nm); Pheneomenex Luna C18 (5 μm, 110 Å, 4.6 × 250 mm), linear gradient of 5% B to 95% B over 30 min, *ca.* 3% B per minute at 1 mL min-1; **MS** (ESI+) *m/z* 1181.6 (calcd for [C52H84N12O19+H]+, 1181.6), 591.3 (calcd for [C52H84N12O19+2H]2+, 591.3).

## Synthesis of the common peptide intermediate 35

Fmoc-Pro-OH was loaded onto 2-CTC resin (0.3 mmol, 0.81 mmol g-1) according to ***General Procedure 1***. The loading yield was calculated as 76% (0.228 mmol, 0.62 mmol g-1) using ***General Procedure 2***. Fmoc-protected linear peptide **6** was assembled using iterative Fmoc deprotections as described in ***General Procedure 3***, standard amino acids were coupled according to ***General Procedure 4***, and non-standard amino acids were coupled according to ***General Procedure 5***. A small portion of the resin was cleaved with TFA:TIPS:H2O (95:2.5:2.5, *v/v/v*, 0.5 mL) for 30 min to confirm the identity of the peptide. The resin was divided into 0.045 mmol or 0.038 mmol portions and the remaining synthesis of analogues **36**-**40** was proceeded at an adjusted scale.


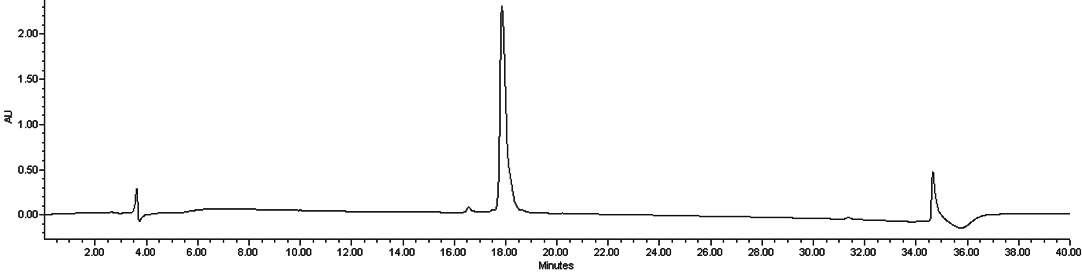


17.86


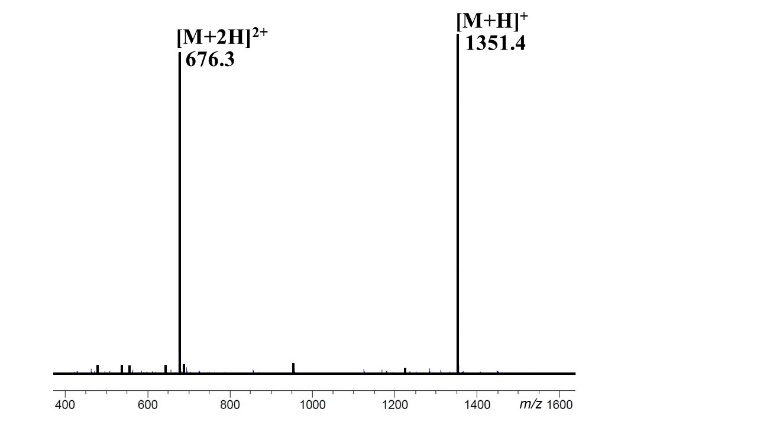


**Figure S2.** Analytical HPLC trace (214 nm) with ESI-MS spectrum of **35** (RT = 17.86 min); Pheneomenex Luna C18 (5 μm, 110 Å, 4.6 × 250 mm), linear gradient of 5%-95% B over 30 min, *ca.* 3% B per minute at 1 mL min-1; **MS** (ESI+) *m/z* 1351.4 (calcd for [C61H82N12O23+H]+, 1350.6), 676.3 (calcd for [C61H82N12O23+2H]2+, 676.3).

## Synthesis of simplified analogue 36

Peptidyl-resin **6** (0.045 mmol) was subjected to Fmoc deprotection according to ***General Procedure 2***. Hexanoic acid was coupled to the *N*-terminus as described in ***General Procedure 5***. Alloc was removed according to ***General Procedure 7*** and the obtained side chain protected linear peptide was cleaved from the resin as described in ***General Procedure 10***. Crude peptide was subjected to overnight macrocyclization according to ***General Procedure 11*** followed by side chain deprotection using ***General Procedure 12***. Purification of crude cyclized peptide by RP-HPLC (Phenomenex Gemini C18 column (110 Å, 5 μm, 250 × 10 mm ) with gradient of 5-65% B at 1% B min-1 until 19% B then 0.5% B, and flow of 4 mL min-1) afforded purified simplified **36** (4.6 mg, 4.1 μmol, 9% yield based on 0.045 mmol scale, *ca*. 99% purity).


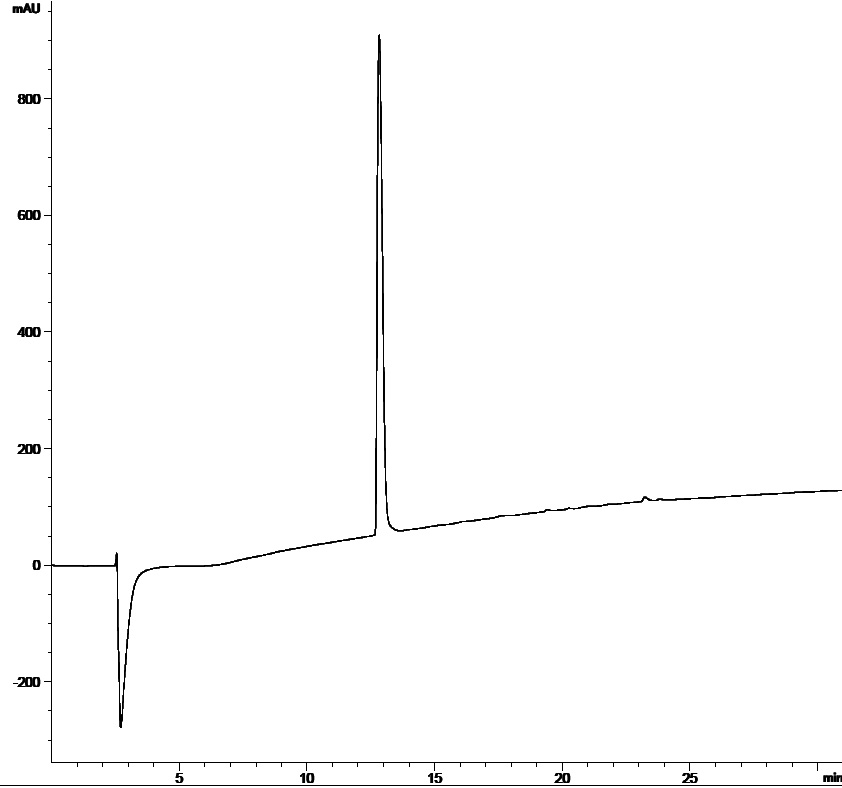

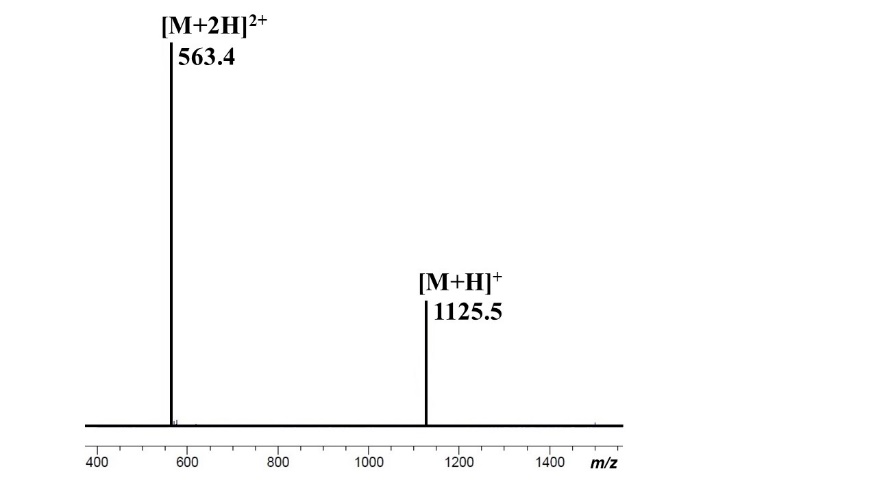


12.83

**Figure S3.** Analytical HPLC trace (210 nm) with ESI-MS spectrum of **36** (RT = 12.83 min); Agilent Zorbax 300SB-C3 column (3.5 μm, 3.0 × 150 mm), linear gradient of 5%-95% B over 30 min, *ca.* 3% B min-1, 0.3 mL min-1; **MS** (ESI+) *m/z* 1125.5 (calcd for [C48H76N12O19+H]+, 1125.5), 563.4 (calcd for [C61H82N12O19+2H]2+, 563.3).

## Synthesis of simplified analogue 37

Peptidyl-resin **35** (0.045 mmol) was subjected to Fmoc deprotection using ***General Procedure 2***. Tetradecanoic acid was coupled to the *N*-terminus as described in ***General Procedure 5***. Alloc was removed according to ***General Procedure 7*** and the obtained side chain protected linear peptide was cleaved from the resin as described in ***General Procedure 10***. Crude peptide was subjected to overnight macrocyclization according to ***General Procedure 11*** followed by side chain deprotection using ***General Procedure 12***. Purification of crude cyclized peptide by RP-HPLC (Phenomenex Gemini C18 column (110 Å, 5 μm, 250 × 10 mm ) with gradient of 5-65% B at 3% B min-1 until 30% B then 0.5% B, and flow of 4 mL min-1) afforded purified simplified **37** (2.7 mg, 2.2 μmol, 5% yield based on 0.045 mmol scale, *ca*. 99% purity).


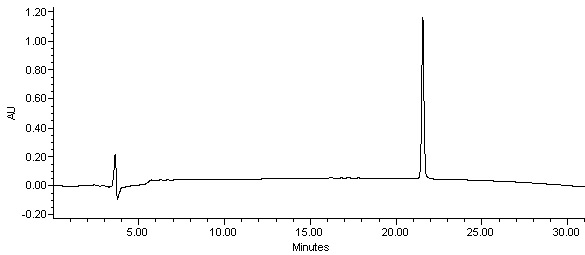

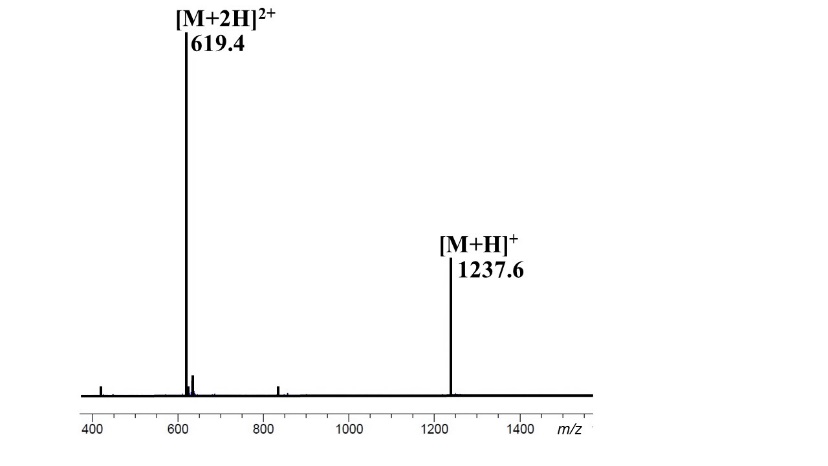


**Figure S4.** Analytical HPLC trace (214 nm) with ESI-MS spectrum of **37** (RT = 21.55 min); Pheneomenex Luna C18 (5 μm, 110 Å, 4.6 × 250 mm), linear gradient of 5%-95% B over 30 min, *ca.* 3% B per minute, 1 mL min-1; **MS** (ESI+) *m/z* 1237.6 (calcd for [C56H92N12O19+H]+, 1237.7), 619.4 (calcd for [C56H92N12O19+2H]2+, 619.3).

## Synthesis of simplified analogue 38

Peptidyl-resin **35** (0.045 mmol) was subjected to Fmoc deprotection using ***General Procedure 2***. Hexadecanoic acid was coupled to the *N*-terminus as described in ***General Procedure 5***. Alloc was removed according to ***General Procedure 7*** and the obtained side chain protected linear peptide was cleaved from the resin as described in ***General Procedure 10***. Crude peptide was subjected to overnight macrocyclization according to ***General Procedure 11*** followed by side chain deprotection using ***General Procedure 12***. Purification of crude cyclized peptide by RP-HPLC (Phenomenex Gemini C18 column (110 Å, 5 μm, 250 × 10 mm ) with gradient of 5-65% B at 3% B min-1 until 30% B then 0.5% B, and flow of 4 mL min-1) afforded purified simplified **38** (3.3 mg, 2.6 μmol, 6% yield based on 0.045 mmol scale, *ca*. 99% purity).


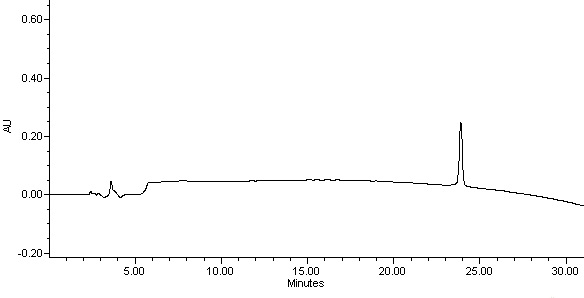


23.89


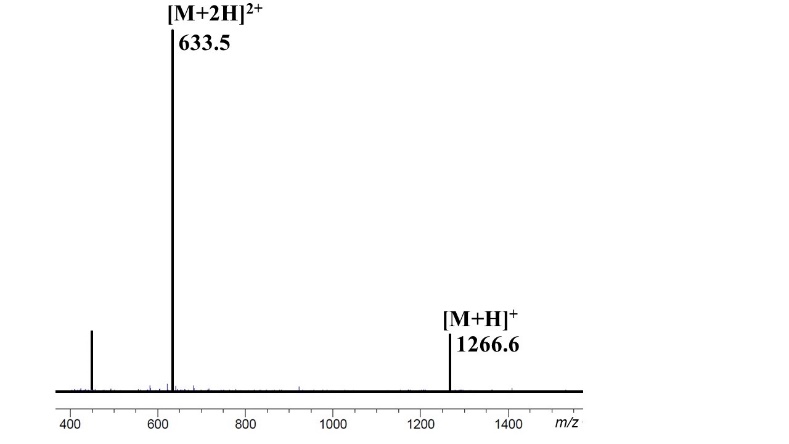


**Figure S5.** Analytical HPLC trace (214 nm) with ESI-MS spectrum of **38** (RT = 23.89 min); Pheneomenex Luna C18 (5 μm, 110 Å, 4.6 × 250 mm), linear gradient of 5%-95% B over 30 min, *ca.* 3% B per minute, 1 mL min-1; **MS** (ESI+) *m/z* 1266.6 (calcd for [C58H96N12O19+H]+, 1265.7), 633.5 (calcd for [C58H96N12O19+2H]2+, 633.4).

## Synthesis of simplified analogue 39

Peptidyl-resin **35** (0.038 mmol) was subjected to Fmoc deprotection using ***General Procedure 2***. 4-Pentylbenzoic acid was coupled to the *N*-terminus as described in ***General Procedure 5***. Alloc was removed according to ***General Procedure 7*** and the obtained side chain protected linear peptide was cleaved from the resin as described in ***General Procedure 10***. Crude peptide was subjected to overnight macrocyclization according to ***General Procedure 11*** followed by side chain deprotection using ***General Procedure 12***. Purification of crude cyclized peptide by RP-HPLC (Phenomenex Gemini C18 column (110 Å, 5 μm, 250 × 10 mm ) with gradient of 5-65% B at 3% B min-1 until 20% B then 0.5% B, and flow of 4 mL min-1) afforded purified simplified **39** (7.6 mg, 6.3 μmol, 17% yield based on 0.038 mmol scale, *ca*. 96% purity).


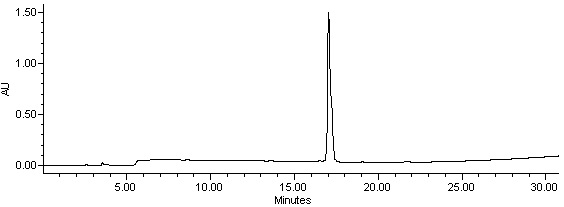

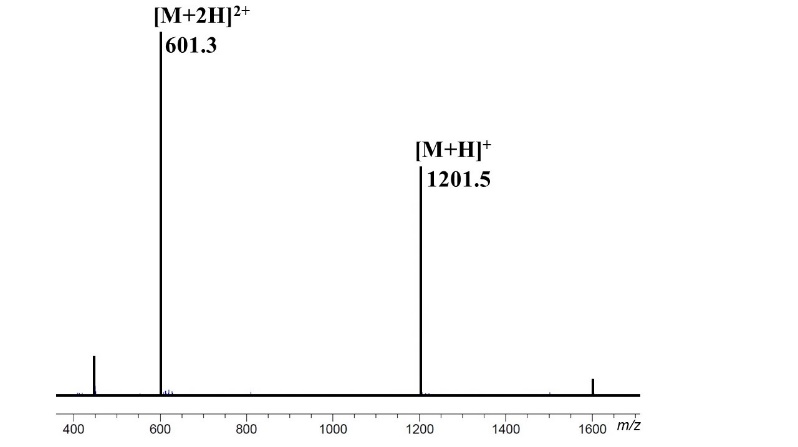


17.03

**Figure S6.** Analytical HPLC trace (214 nm) with ESI-MS spectrum of **39** (RT = 17.03 min); Pheneomenex Luna C18 (5 μm, 110 Å, 4.6 × 250 mm), linear gradient of 5%-95% B over 30 min, *ca.* 3% B per minute, 1 mL min-1; **MS** (ESI+) *m/z* 1201.5 (calcd for [C54H80N12O19+H]+, 1201.6), 601.3 (calcd for [C54H80N12O19+2H]2+, 601.3).

## Synthesis of simplified analogue 40


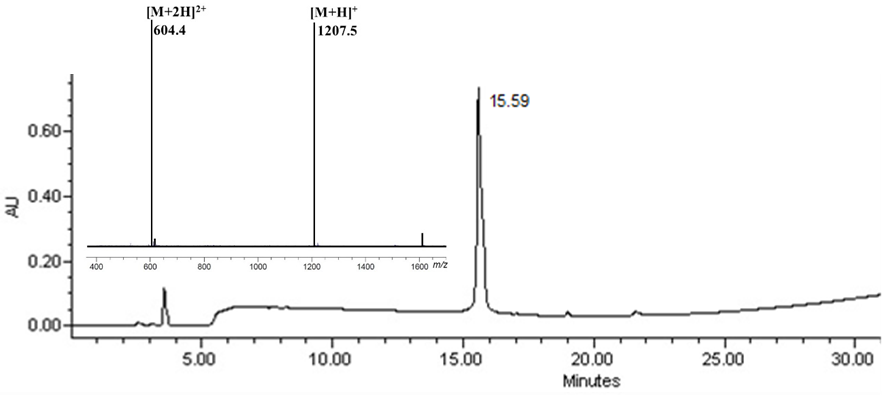
Peptidyl-resin **35** (0.038 mmol) was subjected to Fmoc deprotection using ***General Procedure 2***. Biphenyl-4-carboxylic acid was coupled to the *N*-terminus as described in ***General Procedure 5***. Alloc was removed according to ***General Procedure 7*** and the obtained side chain protected linear peptide was cleaved from the resin as described in ***General Procedure 10***. Crude peptide was subjected to overnight macrocyclization according to ***General Procedure 11*** followed by side chain deprotection using ***General Procedure 12***. Purification of crude cyclized peptide by RP-HPLC (Phenomenex Gemini C18 column (110 Å, 5 μm, 250 × 10 mm ) with gradient of 5-65% B at 1% B min-1 until 15% B then 0.5% B, and flow of 4 mL min-1) afforded purified simplified **40** (6.4 mg, 5.3 μmol, 14% yield based on 0.038 mmol scale, *ca*. 96% purity).

**Figure S7.** Analytical HPLC trace (214 nm) with ESI-MS spectrum of **40** (RT = 15.59 min); Pheneomenex Luna C18 (5 μm, 110 Å, 4.6 × 250 mm), linear gradient of 5%-95% B over 30 min, *ca.* 3% B per minute, 1 mL min-1; **MS** (ESI+) *m/z* 1207.5 (calcd for [C55H74N12O19+H]+, 1207.5), 604.4 (calcd for [C55H74N12O19+2H]2+, 604.3).

## Synthesis of diastereomer 1a

(2*S*,4*R*)-Fmoc-4-MePro-OH (**20**) was loaded onto 2-CTC resin (0.1 mmol, 0.89 mmol g-1) according to ***General Procedure 1***. The loading yield was calculated as 70% (0.07 mmol, 0.62 mmol g-1) using ***General Procedure 2***. The linear peptide was assembled using iterative Fmoc deprotections as described in ***General Procedure 3***, standard amino acids were coupled according to ***General Procedure 4***, non-standard amino acids were coupled according to ***General Procedure 5***, and synthesized amino acid building blocks were coupled according to ***General Procedure 6***. A portion of the resin (0.045 mmol) was subjected to TBS removal at β-OH of (2*S*,3*S*)-3-HyAsp5 residue according to ***General Procedure 8***. The obtained peptide intermediate was Fmoc-deprotected according to ***General Procedure 2*** and the lipid **25** was coupled as described in ***General Procedure 6***. The side chain β-NH(Dde) of (2*S*,3*S*)-3-MeDap2 was subjected to Dde deprotection using ***General Procedure 9*** followed by cleavage from the resin as described in ***General Procedure 10***. The synthesis was proceeded at the 0.045 mmol scale. Crude peptide was subjected to macrocyclization according to ***General Procedure 11*** for 4.5 h followed by side chain deprotection using ***General Procedure 13***. Purification of crude cyclized peptide by RP-HPLC (Phenomenex Gemini C18 column (110 Å, 5 μm, 250 × 10 mm) with gradient of 5%-65% B at 3% B min-1 until 19% B then 0.5% B, and flow of 4 mL min-1, Buffer A 0.1% formic acid in H2O, Buffer B 0.1% formic acid in CH3CN) afforded diastereomeric **1a** (3.9 mg, 3.1 μmol, 7% yield based on 0.045 mmol scale, *ca*. 98% purity).


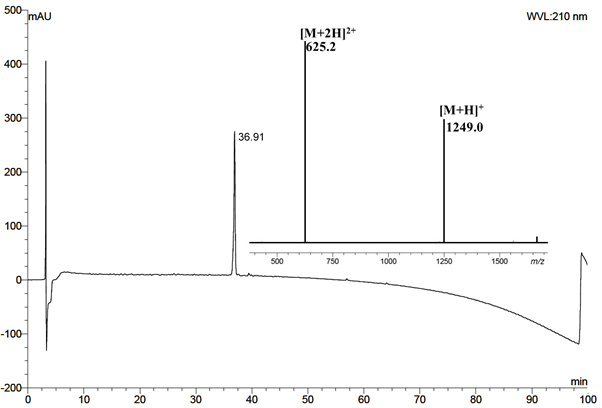


**Figure S8.** Analytical HPLC trace (210 nm) with ESI-MS spectrum of **1a** (RT = 36.91 min); Pheneomenex Gemini NX C18 (5 μm, 110 Å, 4.6 × 250 mm), linear gradient of 5%-95% B over 90 min, *ca.* 1% B per minute, 1 mL min-1; **MS** (ESI+) *m/z* 1249.0 (calcd for [C56H88N12O20+H]+, 1249.6), 625.2 (calcd for [C56H88N12O20+2H]2+, 625.3).

# Stability evaluation to TFA of (2*E*,4*Z*)-8-methylnona-1,2-dienoic acid (25) bound to a model peptide.

## Synthesis and stability evaluation of (2*E*,4*Z*)-8-methylnona-1,2-dienoyl-Ala-Ala-OH (51)

To evaluate stability of lipid **25** to TFA exposure under typical cleavage conditions, a model peptide Ala-Ala acylated with **25** at the *N*-terminus (**52**) was synthesized on pre-loaded Fmoc-Ala-Wang resin. The peptidyl-resin **52** was treated with TFA:TIPS:H2O (95:2.5:2.5, *v/v/v*, 5 mL) for 2 h. Analytical HPLC of the crude **51** showed two major peaks, **51a** and **51b** (**Figure S9a**) in *ca.* 2.4:1 ratio, both of the products showed identical ESI-MS profile suggesting partial *cis-trans* isomerization at the C4-C5 π-bond of the lipid moiety. To determine which peak represented isomerized **51**, the crude was purified by RP-HPLC to separate the two products and 1H NMR was obtained (**Figure S10**). The spectrum of **51a** showed the correct splitting and coupling constants as expected for (2*E*,4*Z*)-diene lipid regiochemistry, whereas the spectrum of **51b** showed splitting and coupling constants as expected for (2*E*,4*E*)-diene lipid regiochemistry. This result confirmed that (2*E*,4*Z*)-lipid moiety was partially unstable to standard TFA cleavage conditions.

Peptide **51** was synthesized using the following method. Fmoc-Ala-Wang resin (0.04 mmol, 55.0 mg, 0.73 mmol g-1) was pre-swollen in DMF (5 mL) for 30 min and Fmoc was removed using ***General procedure 3***. Fmoc-Ala-OH was coupled using ***General procedure 4*** followed by Fmoc removal using ***General procedure 3***. Lipid **25** was coupled according to ***General procedure 6***. Obtained lipidated peptide **52** was cleaved from the resin by treatment with TFA:TIPS:H2O (95:2.5:2.5, *v/v/v*, 5 mL) for 2 h. The filtrate was collected, and the resin was washed with TFA (3 × 1 mL). The filtrate was concentrated under a stream of N2 gas, dissolved in H2O:CH3CN (0.1% TFA, 1:1, *v/v*, 20 mL) and lyophilized. The resulting crude was purified using RP-HPLC (Phenomenex Gemini C18 column (110 Å, 5 µm, 250 × 10 mm) with gradient 5%-65% B at 3%B min-1 until 25% B then 0.5% B, flow 3 mL min-1) to obtain **51a** (3.2 mg, 10.3 µmol, 26% yield) and **51b** (1.5 mg, 4.8 µmol, 12% yield) as white powdery solids. **1H NMR** **51a** (400 MHz, DMSO-*d6*) *δ* 12.49 (br s, 1H, -CO2H), 8.20-8.18 (m, 2H, -NH(Ala1 and Ala2)), 7.30 (ddd, *J* = 15.2, 11.5, 1.0 Hz, 1H, -CH-3), 6.14-6.08 (m, 2H, -CH-4,2), 5.76 (dt, *J* = 10.7, 7.7 Hz,1H, CH-5), 4.41 (quint, *J* = 7.3 Hz, 1H, α-CH-Ala1 or α-CH-Ala2), 4.19 (quint, *J* = 7.3 Hz, 1H, α-CH-Ala1 or α-CH-Ala2), 2.27-2.21 (m, 2H, -CH2-6), 1.55 (sept, *J* = 6.7 Hz, 1H, -CH-8), 1.28-1.21 (m, 8H, -CH3-Ala1 and Ala2,-CH2-7), 0.88 (d, *J* = 6.6 Hz, 6H, -CH3-9) ppm.**1H NMR** **51b** (400 MHz, DMSO-*d6*) *δ* 12.49 (br s, 1H, -CO2H), 8.16 (d, *J* = 7.3 Hz, 1H, -NH(Ala2)), 8.11 (d, *J* = 7.8 Hz, 1H, -NH(Ala1)), 6.99 (dd, *J* = 15.1, 10.6, 1H, -CH-3), 6.18 (dd, *J* = 15.2, 10.7 Hz, 1H, -CH-4), 6.08 (ddd, *J* = 15.2, 6.7, 6.7 Hz, 1H, -CH-5), 6.00 (d, *J* = 15.1 Hz, 1H, CH-2), 4.40 (quint, *J* = 7.2 Hz, 1H, α-CH-Ala1), 4.18 (quint, *J* = 7.3 Hz, 1H, α-CH-Ala2), 2.11-2.16 (m, 2H, -CH2-6), 1.53 (sept, *J* = 6.7 Hz, 1H, -CH-8), 1.30-1.24 (m, 5H, -CH3- Ala2,-CH2-7), 1.21 (d, *J* = 7.1 Hz, 3H, -CH3- Ala1), 0.87 (d, *J* = 6.5 Hz, 6H, -CH3-9) ppm.


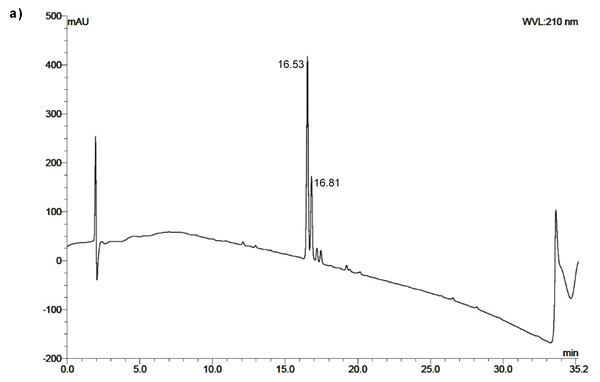


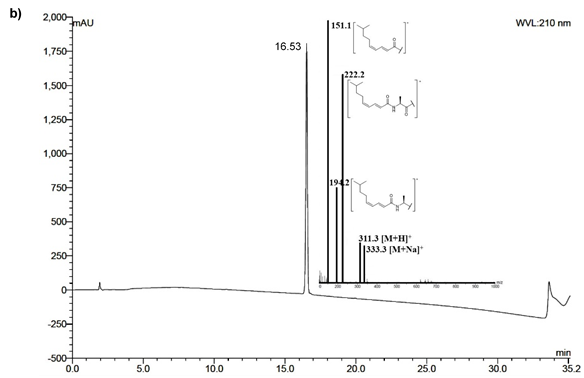


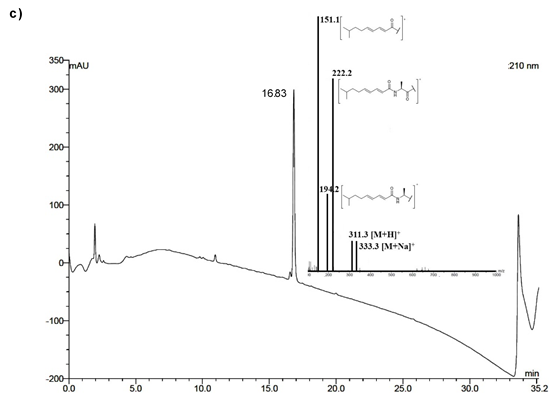


**Figure S9.** a) Analytical HPLC trace (210 nm) of crude **51**, **51a** (RT = 16.53 min, 66% peak area), **51b** (RT = 16.81 min, 27% peak area); b) analytical HPLC trace (210 nm) of purified **51a** with ESI-MS (RT = 16.53 min, *ca*. 99% purity); c) analytical HPLC trace (210 nm) of purified **51b** with ESI-MS (RT = 16.83 min, *ca*. 94% purity); Xterra MS C18 (5 μm, 4.6 × 250 mm), linear gradient of 5%-95% B over 30 min, *ca.* 3% B per minute, 1 mL min-1; **MS** (ESI+) (identical for both **51a** and **51b**) *m/z* 333.3 (calcd for [C16H26N2O4+Na]+, 333.2), 311.3 (calcd for [C16H26N2O4+H]+, 311.2), 222.2 (calcd for [C13H20NO2], 222.2), 194.2 (calcd for [C12H20NO], 194.2), 151.1 (calcd for [C10H15NO], 115.1).


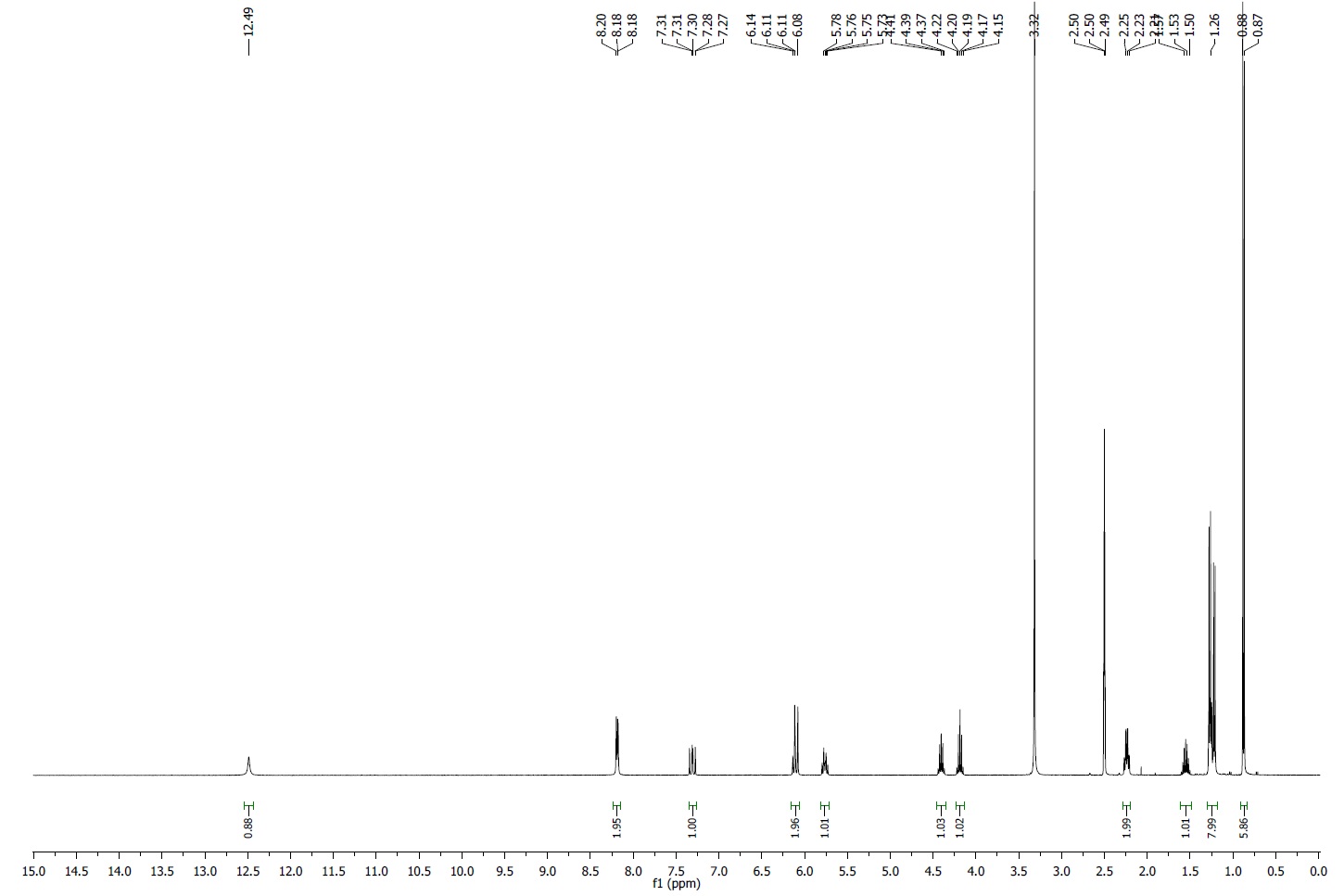


**Figure S10.** 1H NMR spectrum of **51a** (400 MHz, DMSO-*d6*).

**
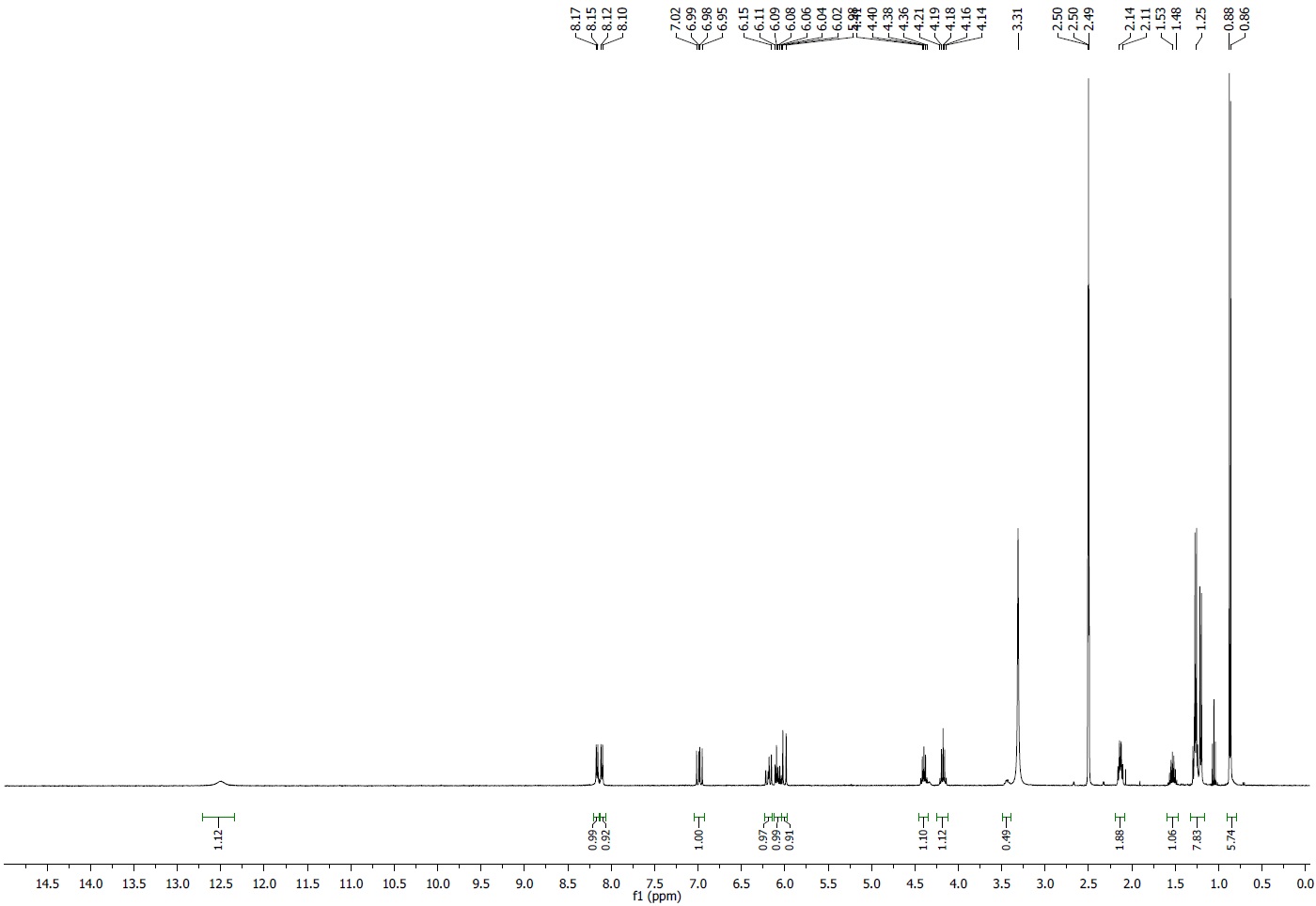
Figure S11.** 1H NMR spectrum of **51b** (400 MHz, DMSO-*d6*).


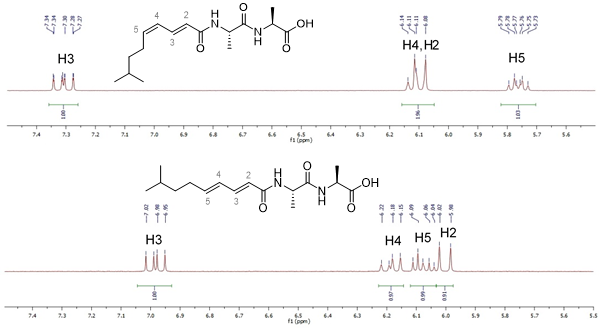


**Figure S12.** Overlay of the 1H NMR diene region of **51a** (top) and **51b** (bottom).

## Synthesis of (2*E*,4*Z*)-8-methylnona-1,2-dienoyl-Ala-Lys-Asp-OH (53) and testing conditions for TFA-mediated cleavage/side chain deprotection

To determine TFA cleavage conditions that would result in minimal isomerization of the (2*E*,4*Z*)-8-methylnona-1,2-dienoyl lipid moiety and complete deprotection of Boc/*t*Bu side chain protection, model lipidated tripeptide **53** was synthesized.

2-CTC resin (0.1 mmol, 0.77 mmol g-1) was loaded with Fmoc-Asp(O*t*Bu)-OH according to ***General Procedure 1***. The loading yield was calculated as 62% (0.062 mmol, 0.48 mmol g-1) using ***General Procedure 2***. The linear peptide was assembled using iterative Fmoc deprotections as described in ***General Procedure 3***, Fmoc-Lys(Boc)-OH and Fmoc-Ala-OH were coupled according to ***General Procedure 4***, and lipid **25** was coupled according to ***General Procedure 6***. A small portion of the resin was subjected to TFA-mediated cleavage/side chain deprotection (**Table S1**). Conditions in Entry 4 were used for side chain deprotection in the synthesis of **1a**.

| Entry | Cleavage conditions | Cleavage time | 53a/% | 53b/% | 53a(O*t*Bu)/% |
| --- | --- | --- | --- | --- | --- |
| 1 | TFA:TIPS:H2O (95:2.5:2.5, *v/v/v*) | 1 h | 74 | 26 | - |
| 2 | TFA:CH2Cl2 (20:80, *v/v*), neutralised with Et3N | 1 h | 86 | 3 | 11 |
| 3 | TFA:TIPS:H2O:CH2Cl2 (20:2.5:2.5:75, *v/v/v/v*), neutralised with Et3N | 1 h | 90 | 4 | 6 |
| 4 | TFA:TIPS:H2O:CH2Cl2 (50:2.5:2.5:45, *v/v/v/v*), neutralised with Et3N | 30 min | 96 | 2 | 2 |
| 5 | TFA:TIPS:H2O:CH2Cl2 (50:2.5:2.5:45, *v/v/v/v*), neutralised with Et3N | 30 min | 94 | 4 | 2 |

**Table S1.** Cleavage of model lipidated tripeptide **54**. Percentage of product formed was determined from relative peak area of analytical HPLCs.


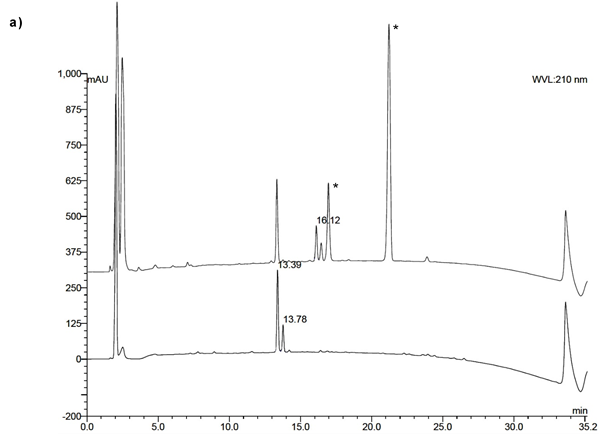


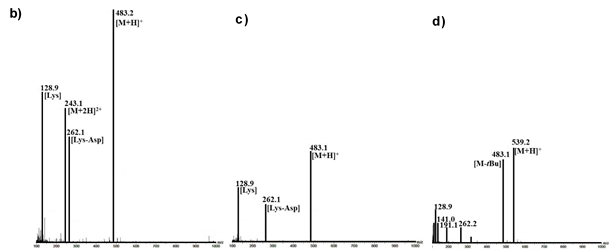


**Figure S13.** a) Analytical HPLC trace (210 nm) of Entry 1 (bottom) and Entry 2 (top) (**Table S1**); **53a** (RT = 13.39min), **53b** (RT = 13.78 min), **53a(O*t*Bu)** (RT = 16.12 min),* denotes peaks arising from Et3N; Xterra MS C18 (5 μm, 4.6 × 250 mm), linear gradient of 5%-95% B over 30 min, *ca.* 3% B per minute, 1 mL min-1; b) **53a** **MS** (ESI+) *m/z* 483.2 (calcd for [C23H38N4O7+H]+, 483.3), 262.1 (calcd for [C10H18N3O5], 243.1 (calcd for [C23H38N4O7+2H]2+, 242.2), 128.9 (calcd for [C6H12N2O], 128.1); c) **53b** **MS** (ESI+) *m/z* 483.1 (calcd for [C23H38N4O7+H]+, 483.3), 262.1 (calcd for [C10H18N3O5], 128.9 (calcd for [C6H12N2O], 128.1); b) **53a(O*t*Bu)** **MS** (ESI+) m/z 539.2 (calcd for [C27H46N4O7+H]+, 539.4), 483.1 (calcd for [C23H38N4O7+H]+, 483.3).

# Antibacterial susceptibility testing

## Minimum inhibitory concentration (MIC) assay of malacidin diastereomer 1a

MIC assays were performed in accordance with the CLSI recommended protocol17 with minor adjustments. Briefly, *Staphylococcus aureus* (ATCC 29213) was grown at 37 oC with shaking (200 rpm) in Mueller Hinton (MH) broth supplemented with either 1.5 mM (near the concentration commonly employed for daptomycin testing [1.25 mM]) or 15 mM CaCl2 (in accordance with the reported testing of malacidin by Hover et al.1) from an autoclaved stock solution of CaCl2. The respective Ca2+ adjusted media was used for all further compound and culture dilutions. A two-fold dilution series of the test compounds was prepared in triplicate in polypropylene 96-well plates. Cultures of bacteria grown for 8 h were diluted accordingly in fresh media before adding 50 μL of inoculum to each well of the MIC plate, to achieve a final volume of 100 μL with a uniform CFU/ml of ~5 x105 in each well. A growth control (untreated) and sterility control (non-inoculated) well were included for each test compound replicate. Plates were incubated at 37 °C with shaking for 18 h before determining the MIC by visual inspection. MIC values were determined as the lowest concentration at which no growth was observed consistently across all three biological replicates of the assay and within triplicates of each test compound.

| ***Staphylococcus aureus***  **ATCC 29213** | | |
| --- | --- | --- |
| **Compound** | **MIC (μg/mL)**  (in presence of 1.5 mM CaCl2) | **MIC (μg/mL)**  (in presence of 15 mM CaCl2) |
| **1a**  **Daptomycin** | >64  0.25 | >64  0.125 |

**Table S2.** MIC values of diastereomer **1a** at different CaCl2 concentrations.

## Minimum inhibitory concentration (MIC) assay of simplified malacidin analogues 31, 36-40

Antimicrobial susceptibility testing was performed in biological triplicate by determining the minimum inhibitory concentrations (MIC) of the six malacidin analogues and the control antibiotics, penicillin G and daptomycin. Malacidin analogues **31**, **36**, **39**, and **40** were prepared as 10 mg mL-1 stock solutions in filter sterilised MilliQ water. Compounds **37**, and **38** were prepared as 5 mg mL-1 stock solutions in filter sterilised MilliQ water. Malacidin analogues were tested at a maximum concentration of 128 µg mL-1. Bacterial inoculums of *Staphylococcus aureus* ATCC 6538 were prepared by diluting an overnight culture to a final optical density 600 nm (OD600) of 0.003 in cation adjusted Mueller Hinton broth (CAMH) to yield 5 × 105 CFU mL-1 (final) in all assays. MIC assays were performed in 96-well microtiter plates in a total volume of 100 µL, and compounds were serially diluted across the plate in 2-fold dilutions. The MIC was reported as the lowest concentration of the test compound for which no growth occurred after incubation at 37 °C for 18 to 24 h. For all MIC testing CAMHB was supplemented with calcium (50 mg L-1 final) as per the CLSI guidelines for daptomycin17

| ***Staphylococcus aureus***  **ATCC 6538** | |
| --- | --- |
| **Compound** | **MIC (μg/mL)a** |
| **31** | >128 |
| **36** | >128 |
| **37**  **38** | >128  >128 |
| **39**  **40** | >128  >128 |
| **Penicillin G**  **Daptomycin** | 0.03125  1 |

**Table S3.** MIC values of simplified analogues **31, 36-40** in presence of 50 mg L-1 of CaCl2.aTesting was additionally performed in the absence of CaCl2 and no activity for any of the malacidin analogues was observed against *S. aureus.*

# 1H and 13C NMR spectra of diastereomer 1a


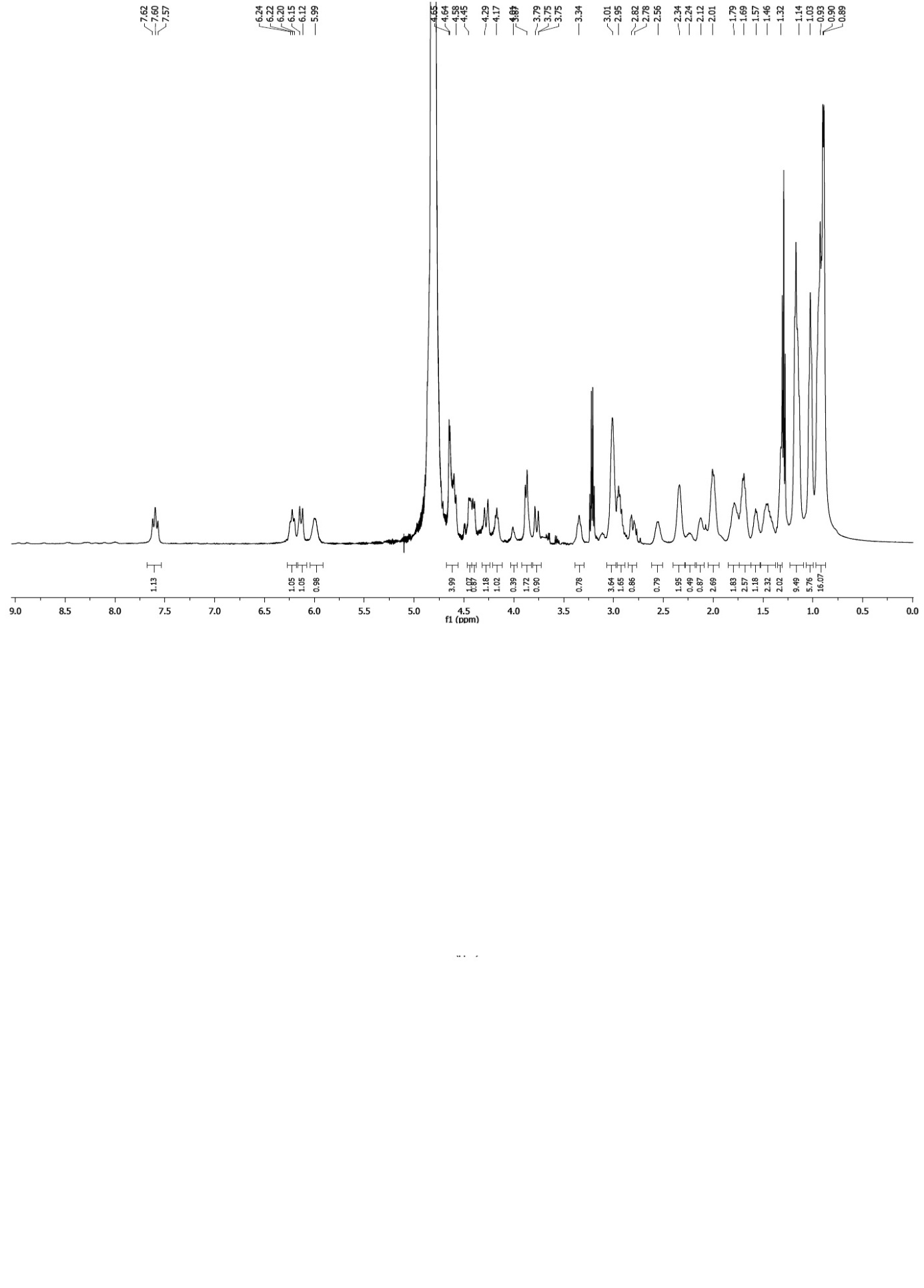


**Figure S14.** 1H NMR spectrum of **1a** (5.6 mM) recorded at 500 MHz, 298 K and referenced to CH3 of Et3N (1.88 mM, *δ*H1.292) in D2O.


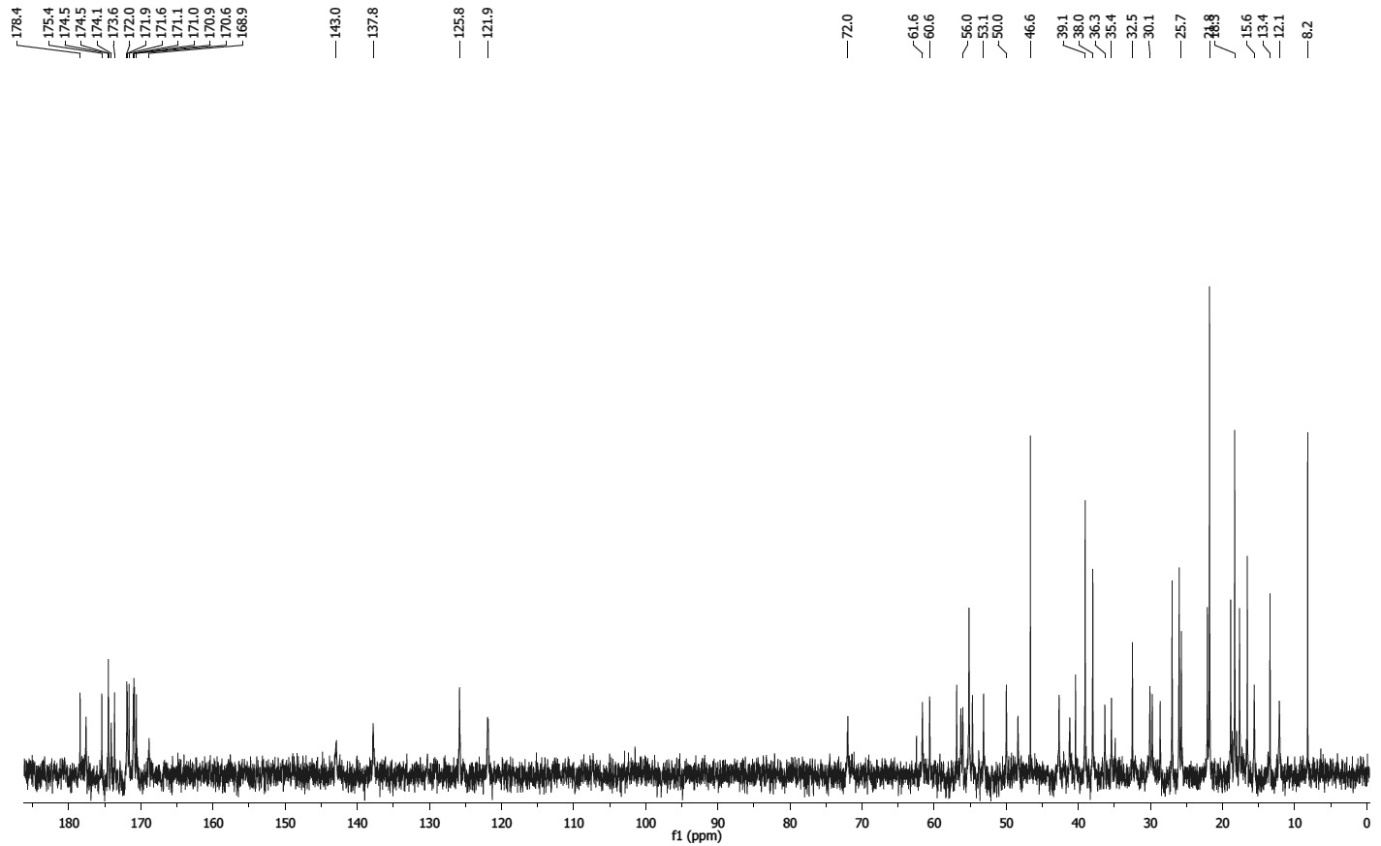


**Figure S15.** 13C NMR spectrum of **1a** (5.6 mM) recorded at 125 MHz, 298 K and referenced to CH3 of Et3N (1.88 mM, *δ*C8.189) in D2O.


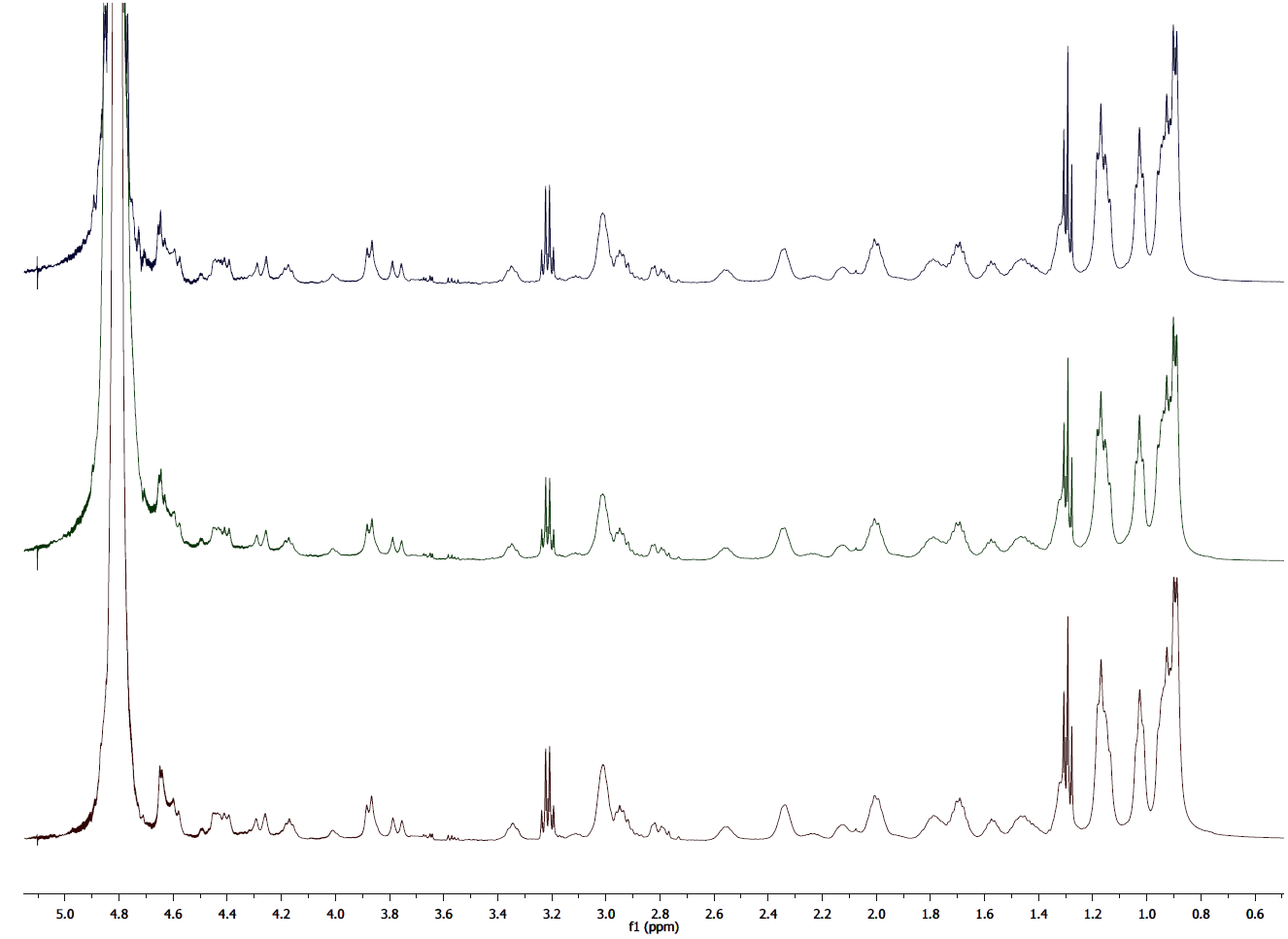
**Figure S16.** Overlay of 1H NMR spectra of synthetic **1a** (5.6 mM) recorded at 500 MHz, 298 K in D2O spiked with Et3N (1.88 mM) and CaCl2 of varying concentrations: 0 mM CaCl2 (bottom), 1.5 mM CaCl2 (middle), 15 mM CaCl2 (top).

# 1H and 13C NMR spectra of building blocks

1H NMR spectrum for **3** (400 MHz, CDCl3)

13C NMR spectrum for **3** (100 MHz, CDCl3)

1H NMR spectrum for **4** (400 MHz, CDCl3)

13C NMR spectrum for **4** (100 MHz, CDCl3)

1H NMR spectrum for **5** (400 MHz, D2O)

13C NMR spectrum for **5** (100 MHz, D2O)

1H NMR spectrum for **6** (400 MHz, D2O)

13C NMR spectrum for **6** (100 MHz, D2O)

1H NMR spectrum for **2** (400 MHz, CDCl3)

13C NMR spectrum for **2** (100 MHz, CDCl3)

1H NMR spectrum for **44** (400 MHz, CDCl3)

13C NMR spectrum for **44** (100 MHz, CDCl3)

1H NMR spectrum for **45** (400 MHz, CDCl3)

13C NMR spectrum for **45** (100 MHz, CDCl3)

1H NMR spectrum for **46** (400 MHz, D2O)

13C NMR spectrum for **46** (100 MHz, D2O)

1H NMR spectrum for **47** (400 MHz, D2O)

13C NMR spectrum for **47** (100 MHz, D2O)

1H NMR spectrum for **7** (400 MHz, CDCl3)

13C NMR spectrum for **7** (100 MHz, CDCl3)

1H NMR spectrum for **9** (400 MHz, CDCl3)

13C NMR spectrum for **9** (100 MHz, CDCl3)

1H NMR spectrum for **10** (400 MHz, CDCl3)

13C NMR spectrum for **10** (100 MHz, CDCl3)

1H NMR spectrum for **11** (400 MHz, CDCl3)

13C NMR spectrum for **11** (100 MHz, CDCl3)

1H NMR spectrum for **13** (400 MHz, CDCl3)

13C NMR spectrum for **13** (100 MHz, CDCl3)

1H NMR spectrum for **15** (400 MHz, CDCl3)

13C NMR spectrum for **15** (100 MHz, CDCl3)

1H NMR spectrum for **16** (400 MHz, CDCl3)

13C NMR spectrum for **16** (100 MHz, CDCl3)

1H NMR spectrum for **17** (400 MHz, CDCl3)

13C NMR spectrum for **17** (100 MHz, CDCl3)

1H NMR spectrum for **18** (400 MHz, CDCl3)

13C NMR spectrum for **18** (100 MHz, CDCl3)

1H NMR spectrum for **14** (400 MHz, CDCl3)

13C NMR spectrum for **14** (100 MHz, CDCl3)

1H NMR spectrum for **19** (400 MHz, CDCl3)

13C NMR spectrum for **19** (100 MHz, CDCl3)

1H NMR spectrum for **21** (400 MHz, CDCl3)

13C NMR spectrum for **21** (100 MHz, CDCl3)

1H NMR spectrum for **22** (400 MHz, CDCl3)

13C NMR spectrum for **22** (100 MHz, CDCl3)

1H NMR spectrum for **23** (400 MHz, CDCl3)

13C NMR spectrum for **23** (100 MHz, CDCl3)

1H NMR spectrum for **24** (400 MHz, CDCl3)

13C NMR spectrum for **24** (100 MHz, CDCl3)

1H NMR spectrum for **20** (400 MHz, CDCl3)

13C NMR spectrum for **20** (100 MHz, CDCl3)

1H NMR spectrum for **26** (400 MHz, CDCl3)

13C NMR spectrum for **26** (100 MHz, CDCl3)

1H NMR spectrum for **27** (400 MHz, CDCl3)

13C NMR spectrum for **27** (100 MHz, CDCl3)

1H NMR spectrum for **28** (400 MHz, CDCl3)

13C NMR spectrum for **28** (100 MHz, CDCl3)

1H NMR spectrum for **29** (400 MHz, CDCl3)

13C NMR spectrum for **29** (100 MHz, CDCl3)

1H NMR spectrum for **30** (400 MHz, CDCl3)

13C NMR spectrum for **30** (100 MHz, CDCl3)

1H NMR spectrum for **25** (400 MHz, CDCl3)

13C NMR spectrum for **25** (100 MHz, CDCl3)

# References

(1) Hover, B. M.; Kim, S.-H.; Katz, M.; Charlop-Powers, Z.; Owen, J. G.; Ternei, M. A.; Maniko, J.; Estrela, A. B.; Molina, H.; Park, S.; Perlin, D. S.; Brady, S. F. Culture-Independent Discovery of the Malacidins as Calcium-Dependent Antibiotics with Activity against Multidrug-Resistant Gram-Positive Pathogens. *Nat. Microbiol.* **2018**, *3* (4), 415–422. https://doi.org/10.1038/s41564-018-0110-1.

(2) Sun, Z.; Shang, Z.; Forelli, N.; Po, K. H. L.; Chen, S.; Brady, S. F.; Li, X. Total Synthesis of Malacidin A by β-Hydroxyaspartic Acid Ligation-Mediated Cyclization and Absolute Structure Establishment. *Angewandte Chemie - International Edition* **2020**, *59* (45), 19868–19872. https://doi.org/10.1002/anie.202009092.

(3) Hwang, T. L.; Shaka, A. J. Water Suppression That Works. Excitation Sculpting Using Arbitrary Wave-Forms and Pulsed-Field Gradients. *Journal of Magnetic Resonance, Series A* **1995**, *112* (2), 275–279. https://doi.org/10.1006/jmra.1995.1047.

(4) Xue, C. B.; He, X.; Roderick, J.; Corbett, R. L.; Decicco, C. P. Asymmetric Synthesis of Trans-2,3-Piperidinedicarboxylic Acid and Trans-3,4-Piperidinedicarboxylic Acid Derivatives. *Journal of Organic Chemistry* **2002**, *67* (3), 865–870. https://doi.org/10.1021/jo016086b.

(5) Schabbert, S.; Pierschbacher, M. D.; Mattern, R.; Goodman, M. Incorporation of (2S,3S) and (2S,3R) β-Methyl Aspartic Acid into RGD-Containing Peptides. *Bioorganic & Medicinal Chemistry* **2002**, *10*, 3331–3337. https://doi.org/10.1016/S0968-0896(02)00206-7.

(6) Horlacher, O. P.; Hartkoorn, R. C.; Cole, S. T.; Altmann, K. H. Synthesis and Antimycobacterial Activity of 2,1′-Dihydropyridomycins. *ACS Medicinal Chemistry Letters* **2013**, *4* (2), 264–268. https://doi.org/10.1021/ml300385q.

(7) Martín, M. J.; Rodríguez-Acebes, R.; García-Ramos, Y.; Martínez, V.; Murcia, C.; Digón, I.; Marco, I.; Pelay-Gimeno, M.; Fernández, R.; Reyes, F.; Francesch, A. M.; Munt, S.; Tulla-Puche, J.; Albericio, F.; Cuevas, C. Stellatolides, a New Cyclodepsipeptide Family from the Sponge Ecionemia Acervus: Isolation, Solid-Phase Total Synthesis, and Full Structural Assignment of Stellatolide A. *J. Am. Chem. Soc.* **2014**, *136* (18), 6754–6762. https://doi.org/10.1021/ja502744a.

(8) Armaly, A. M.; Bar, S.; Schindler, C. S. Acid Chlorides as Formal Carbon Dianion Linchpin Reagents in the Aluminum Chloride-Mediated Dieckmann Cyclization of Dicarboxylic Acids. *Organic Letters* **2017**, *19* (15), 3962–3965. https://doi.org/10.1021/acs.orglett.7b01623.

(9) Moreira, R.; Taylor, S. D. Asymmetric Synthesis of Fmoc-Protected β-Hydroxy and β-Methoxy Amino Acids via a Sharpless Aminohydroxylation Reaction Using FmocNHCl. *Organic Letters* **2018**, *20* (23), 7717–7720. https://doi.org/10.1021/acs.orglett.8b03458.

(10) Gwon, D.; Hwang, H.; Kim, H. K.; Marder, S. R.; Chang, S. Synthesis of 8-Aminoquinolines by Using Carbamate Reagents: Facile Installation and Deprotection of Practical Amidating Groups. *Chemistry - A European Journal* **2015**, *21* (48), 17200–17204. https://doi.org/10.1002/chem.201503511.

(11) Koskinen, A. M. P.; Helaja, J.; Kumpulainen, E. T. T.; Koivisto, J.; Mansikkamäki, H.; Rissanen, K. Locked Conformations for Proline Pyrrolidine Ring:  Synthesis and Conformational Analysis of Cis- and Trans-4-Tert-Butylprolines. *J. Org. Chem.* **2005**, *70* (16), 6447–6453. https://doi.org/10.1021/jo050838a.

(12) Chabaud, P.; Pèpe, G.; Courcambeck, J.; Camplo, M. Stereoselective Synthesis of (3S,4S)-Tert-Butyl-N-Boc-3-Ethyl-4-Hydroxy-l-Prolinate and (3S,4R)-Tert-Butyl-N-Boc-3-Ethyl-4-Hydroxy-l-Prolinate. *Tetrahedron* **2005**, *61* (15), 3725–3731. https://doi.org/10.1016/j.tet.2005.02.006.

(13) Durand, X.; Hudhomme, P.; Khan, J. A.; Young, D. W. Synthesis of (2S,4S)- and (2S,4R)-5,5′-Dihydroxy[5,5-2H2]Leucine by Two Independent Routes. *J. Chem. Soc., Perkin Trans. 1* **1996**, No. 11, 1131–1139. https://doi.org/10.1039/P19960001131.

(14) Nevalainen, M.; Kauppinen, P. M.; Koskinen, A. M. P. Synthesis of Fmoc-Protected Trans-4-Methylproline. *J. Org. Chem.* **2001**, *66* (6), 2061–2066. https://doi.org/10.1021/jo005726s.

(15) Goodreid, J. D.; Dos Santos Da Silveira, E.; Batey, R. A. A Lanthanide(III) Triflate Mediated Macrolactonization/Solid-Phase Synthesis Approach for Depsipeptide Synthesis. *Organic Letters* **2015**, *17* (9), 2182–2185. https://doi.org/10.1021/acs.orglett.5b00781.

(16) Chan, W. C.; White, P. D. *Fmoc Solid Phase Peptide Synthesis: A Practical Approach*; Oxford University Press, 2000.

(17) Clinical and Laboratory Standards Institute. M100-S16, Performance Standards for Antimicrobial Susceptibility Testing; 16th Informational Supplement. Clinical and Laboratory Standards Institute, Wayne, PA 2006.
